# Advancements in Understanding the Physicochemical Properties of Reticular Materials: An In Situ and Operando Spectroscopic Perspective

**DOI:** 10.1002/adma.202415135

**Published:** 2025-02-24

**Authors:** Bettina Baumgartner, Anna Wach, Xinwei Ye, Evelyn Ploetz, Bert M. Weckhuysen

**Affiliations:** ^1^ Van't Hoff Institute for Molecular Sciences University of Amsterdam Science Park 904 Amsterdam 1098 XH the Netherlands; ^2^ SOLARIS National Synchrotron Radiation Centre Jagiellonian University Krakow 30‐392 Poland; ^3^ Institute of Physical Chemistry Polish Academy of Sciences Warsaw 01‐224 Poland; ^4^ Debye Institute for Nanomaterials Science and Institute for Sustainable and Circular Chemistry Utrecht University Universiteitsweg 99 Utrecht 3584 CG The Netherlands; ^5^ Department of Chemistry and Center of NanoScience (CeNS) Ludwig‐Maximilians‐Universität München Butenandtstr. 5‐13 81377 Munich Germany

**Keywords:** catalysis, covalent‐organic frameworks, in situ and operando techniques, metal–organic frameworks, microscopy, spectroscopy

## Abstract

The application of in situ and operando spectroscopic techniques has significantly advanced the understanding of reticular materials, particularly metal–organic frameworks (MOFs) and covalent organic frameworks (COFs). These techniques offer real‐time insights into the dynamic structural, electronic, and chemical changes that occur within these materials during various processes, such as catalysis, sorption, and material synthesis. This review offers a comprehensive overview of key in situ and operando techniques used to investigate the formation, functionalization, and catalytic behavior of reticular materials. How these techniques have elucidated the roles of active sites, reaction intermediates, and structural transformations under reaction conditions, especially in single‐site catalysis, electrocatalysis, and photocatalysis, is highlighted. The review also discusses the challenges and opportunities that lie ahead in integrating advanced spectroscopic methods with reticular materials, aiming to foster further innovation in the design and application of these versatile materials.

## Graphical abstract

This review explores how in situ and operando spectroscopic techniques reveal the real‐time behavior of reticular materials, including MOFs and COFs. These methods track material formation and functionalization, structural changes, defect formation, dynamic responses to external triggers, and catalytic processes. Key findings and future opportunities are highlighted, offering pathways to enhance the design and functionality of these materials.

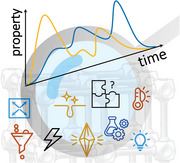

## 1 The Promise of In Situ and Operando Spectroscopy and the Scope of the Review

Reticular materials, including metal–organic frameworks (MOFs), zeolite imidazolate frameworks (ZIFs) as a subclass of MOFs, and covalent organic frameworks (COFs) are defined by their network‐like structures. These structures consist of nodes, which can be metal ions in MOFs and ZIFs or organic molecules in COFs, connected by organic linkers. Their porous architecture offers remarkable tunability in both structure and composition, allowing for precise modification of properties. These abilities make reticular materials suitable for applications ranging from energy storage, gas storage and separation,^[^ ^1^ ^]^ catalysis and environmental remediation,^[^ ^2^, ^3^, ^4^, ^5^ ^]^ drug delivery,^[^ ^6^ ^]^ sensing,^[^ ^7^, ^8^ ^]^ optoelectronics, and photovoltaics to many more, as recently reviewed elsewhere.^[^ ^3^, ^4^, ^6^, ^8^, ^9^, ^10^, ^11^, ^12^, ^13^, ^14^, ^15^, ^16^, ^17^, ^18^, ^19^, ^20^, ^21^, ^22^, ^23^, ^24^, ^25^ ^]^ Moreover, these materials can be engineered across various length scales, from nanoparticles to films, crystals and monolithic structures, further broadening their application potential.

**Figure 1:**
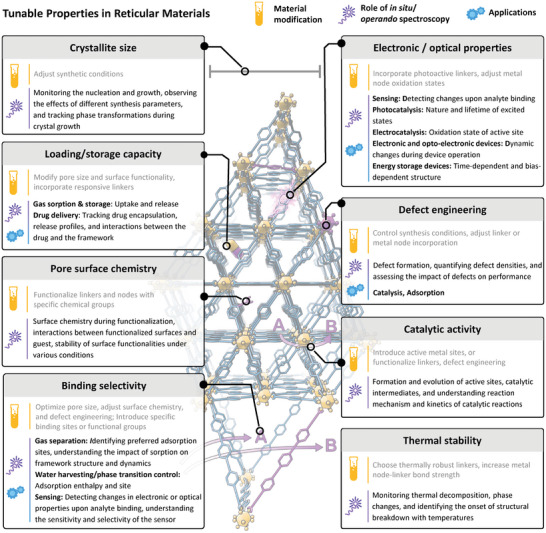
The role of in situ*/*operando spectroscopy to study the tunable properties in reticular materials such as crystallite size, loading/storage capacity, pore surface chemistry, and binding selectivity and how they can be adjusted through synthetic control, functionalization, and defect engineering.

To fully exploit their potential, it is essential to understand the dynamic behavior of these materials under working conditions. Traditionally, ex situ spectroscopic techniques have been used to confirm their composition and structure post‐synthesis or post‐reaction, offering a static snapshot of their properties. However, these methods fail to capture real‐time changes and heterogeneous distributions that occur during synthesis and operation. In contrast, in situ and operando spectroscopic techniques allow direct observation of these materials as they undergo physical and chemical changes, providing crucial insights into their behavior under operational conditions. These techniques have grown remarkably powerful in recent years, allowing researchers to monitor, understand and afterwards fine‐tune the material's performance. **Figure** **1** provides an overview of the tunable properties of reticular materials, how these properties can be modified to suit different applications and how they are studied using in situ and operando spectroscopy.

As we explore these techniques further, it is important to clarify the differences between “in situ” and “operando”:

In situ spectroscopy, meaning “in place” in Latin, involves observing material properties or chemical reaction under controlled environments approximating working conditions. While the system may not perfectly mimic its real‐world environment, in situ techniques allow real‐time monitoring of physical or chemical changes without altering the material due to the measurement itself. In situ spectroscopy provides valuable information about formation of initial pre‐nucleation species, material assembly, crystallization, and phase transitions without disrupting the studied process in the material. These insights are essential for understanding how reticular materials react upon different triggers or under catalytic conditions.

Operando spectroscopy goes a step further by studying a material in its active state, performing its function under specific operational conditions while simultaneously monitoring its activity.^[^ ^26^, ^27^ ^]^ This method allows researchers to directly correlate spectroscopic data with the functionality of the material, e.g., catalytic activity, stability, charging of battery materials, etc. To achieve this, specialized reactors are often designed to meet the requirements for both the operational process—such as a catalytic reaction—and the in situ spectroscopic measurements.^[^ ^28^, ^29^, ^30^ ^]^

When not integrated with microscopy approaches, in situ and operando techniques are employed to monitor the temporal evolution of chemical reactions or observe physical responses within reticular systems. For this, they are either combined with pump‐probe spectroscopy schemes for following molecular processes on the femto‐ to picosecond time scale via ultra‐fast laser spectroscopy (which we will not touch upon in this review),^[^ ^31^, ^32^, ^33^, ^34^, ^35^, ^36^, ^37^ ^]^ or implemented as time‐resolved measurements on the nano‐, microsecond up to hour time scale to monitor dynamic processes like adsorption, diffusion or catalytic activity. Finally, the ability to resolve these processes in time is essential for understanding reaction kinetics, especially when influenced by external triggers like light, electric or magnetic fields, or chemical reagents. Here, time‐resolved spectroscopy allows us to capture how systems evolve in response to these triggers, providing insights that are central to the study of dynamic processes.

While investigating adsorption sites and pore sizes in reticular materials using probe molecules is a common approach—often employing in situ techniques to measure framework interactions—these methods are generally passive, probing only the static properties of the material in absence of an external stimulus or chemical conversions.^[^ ^38^, ^39^ ^]^ Although these studies are valuable for understanding the material's structure and adsorption sites, they fall outside the scope of this review. Our focus lies on the dynamic properties of materials and process‐oriented aspects, specifically in situ and operando measurements that study framework dynamics, interactions with the guest molecules or other related processes.

In this review, we aim to guide readers through the applications of in situ and operando spectroscopic and microscopic techniques, with a focus on reticular materials. We will explore how these tools can shed light on dynamic processes such as material formation, functionalization, structural changes under varying conditions, defect formation, and their roles in catalytic reactions like thermocatalysis, photocatalysis, and electrocatalysis. Rather than categorizing by applied spectroscopic technique, we explore them through the perspective of key dynamic processes. Given the extensive literature on these topics, our goal is to provide a structured and accessible overview for both newcomers and experienced researchers. We aim to offer a practical reference guide and provide relevant, more in‐depth literature via references throughout the review. The review is divided into three sections: the first chapter introduces the fundamentals of spectroscopy and microscopy for newcomers; the second chapter discusses advanced, state‐of‐the‐art techniques and their applications in the field of reticular material chemistry; and the third chapter explores emerging challenges and potential advancements.

## 2 Methodological Approaches for In Situ and Operando Studies

**Figure 2:**
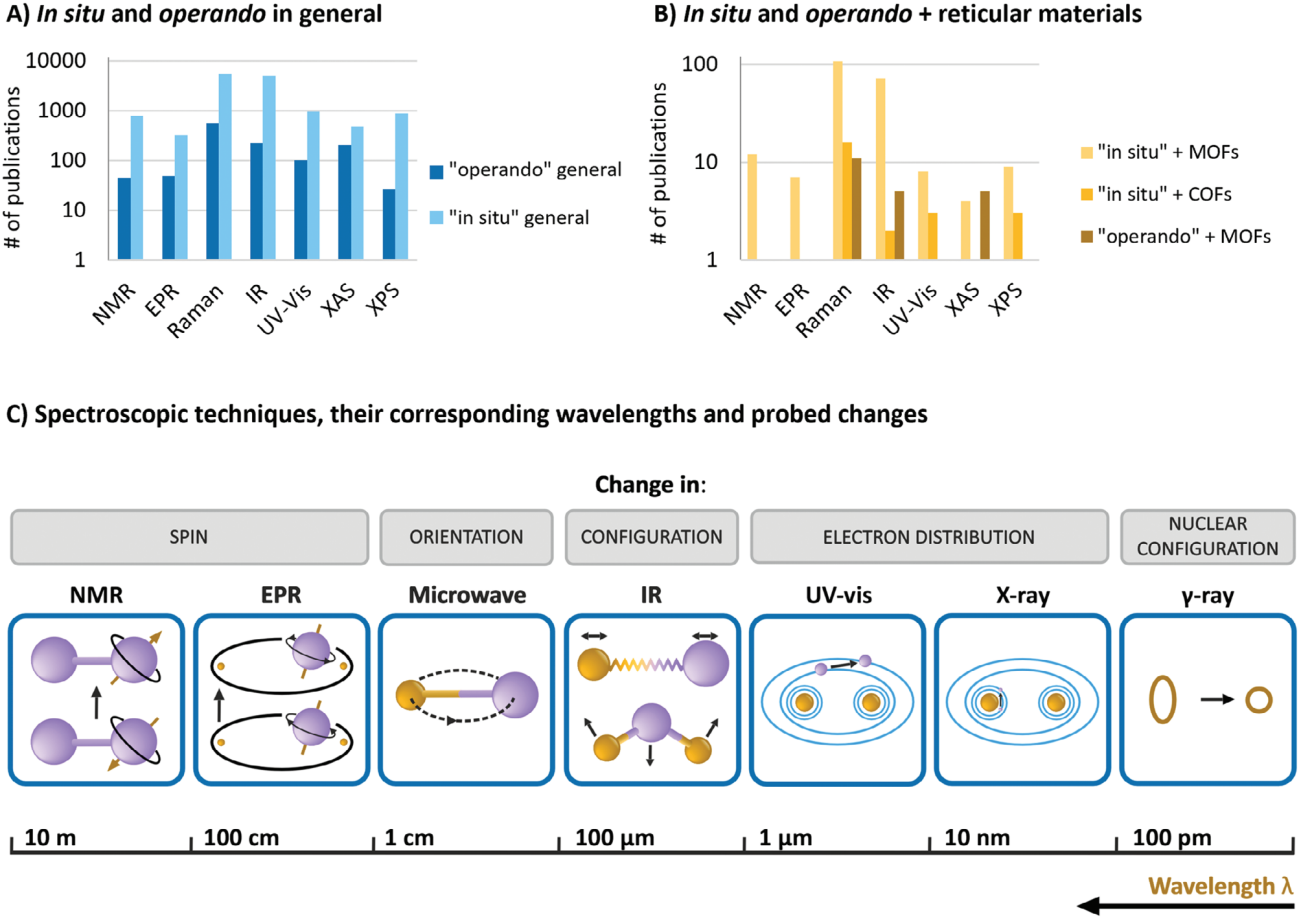
Concept of operando methodologies and their application. A,B) Evolution of publications in the field of in situ and operando spectroscopy sorted according to the employed technique for all applications (A) in general (B) and for reticular materials. Data was obtained by searching for “operando”, “technique”, “MOF” or “COF”, “research article” in Web of Science. See Supporting Information for exact numbers and further info. C) Overview of light‐matter interactions underlying spectroscopic techniques for probing changes in reticular materials, detailing the relevant wavelength, frequency, energy, and wavenumber regions, respectively. X‐ray radiation (λ_0_ =  10 – 0.01 nm) induces changes in the states of core electrons, as observed in techniques such as Auger‐electron spectroscopy and X‐ray spectroscopy. Ultraviolet and visible radiation (λ_0_ =  10–1000 nm) allows for probing electronic transitions of valence electrons, as probed in Fluorescence and UV–vis absorption spectroscopy, as well as vibrational transition via Raman scattering spectroscopy. Complementary, infrared radiation (λ_0_ =  0.7–100 µm) can induce vibrational transitions of the molecule, that can be probed by IR absorption spectroscopy. Terahertz (THz) radiation (λ_0_ =  0.1–1 mm) probes rotational movements of the molecule in the gas phase or lattice vibrations in crystals, as probed in rotational spectroscopy. Microwaves (λ_0_ =  1–100 cm) excite electron spins, as used in electron paramagnetic resonance (EPR). Radio waves (λ_0_ =  1–100 m) induce changes in spins of atomic nuclei, as used in nuclear magnetic resonance (NMR) spectroscopy.

We begin with a brief tutorial on the spectroscopic techniques commonly used in in situ and operando experiments, tailored for newcomers as well as for experienced spectroscopists looking to expand beyond their familiar wavelength range. In our literature review, we identified the most frequently used spectroscopic techniques both in the field of in situ/operando spectroscopy and reticular chemistry, as summarized in **Figure** **2**A,B. These techniques include, in order of increasing wavelength: X‐ray based techniques (XAS, XES, XPS), UV–vis absorption and fluorescence spectroscopy, vibrational spectroscopy methods based on infrared (IR) absorption spectroscopy and Raman scattering, electron paramagnetic resonance (EPR) spectroscopy, and NMR spectroscopy. As shown in Figure 2, vibrational spectroscopies are the most widely used techniques for operando and in situ studies but also for investigating processes in MOFs and COFs. Their prominence can be attributed to several factors: their widespread availability compared to advanced techniques like XAS and XPS, their ease of integration into experimental setups (e.g., Raman spectroscopy with optical fiber probes), and their ability to provide highly detailed molecular‐level insights through the vibrational fingerprint region, which often offers more specific structural information than UV–vis spectroscopy.

Although less commonly employed, we further provide an introduction to Mössbauer spectroscopy, THz spectroscopy and impedance spectroscopy. For each technique, we offer a brief introduction to their underlying physical principles and summarize the chemical information that can be obtained, the experimental configurations, advantages and limitations in **Table** **1**. Additionally, we have included references to more in‐depth reviews and textbooks for each technique.

**Table 1:** Overview in situ and operando spectroscopies used to study reticular materials.

Technique	Wavelength	Available information	Experimental configuration	Experimental limitations	t_R_	In situ application	Advantages	Useful textbooks and reviews
Mössbauer spectroscopy	< 10 pm > 100 keV (γ‐radiation)	Probing small changes in chemical environment Spin states with high sensitivity	Transmission backscattering	limited number of nuclei in practical application, such as^[^ ^57^ ^]^ Fe,^[^ ^116^ ^]^ Sn	s – min	heterogeneous catalysis	Highly specific to certain isotopes High sensitivity to local environments around Mössbauer‐active nuclei	general^[^ ^66^, ^67^ ^]^ In situ*/*operando methods for heterogeneous catalysis^[^ ^68^ ^]^
X‐ray Absorption spectroscopy (XAS)	10‐0.012 nm (0.1‐ 100 keV)	Electronic and structural properties of matter X‐ray absorption near edge structure (XANES): oxidation state and coordination chemistry of the absorbing atom; unoccupied electronic states Edge X‐ray absorption fine structure (EXAFS): atom local environment, yielding the number, type and distances of neighboring atoms	Transmission Fluorescence Total electron Yield	Provides average properties of a sample (XAS is a bulk technique) Requires a high brilliance and energy‐tunable X‐ray source providing sufficient photon flux (synchrotron sources)	ms‐ min	catalysis, photocatalysis, electrocatalysis, electrochemistry, materials formation and characterization	Element‐specific method well‐suited to probe the active sites in multielement systems can be applied to the investigation of a broad range of materials (ordered, disordered, nanostructured, liquids) and under working conditions	General^[^ ^69^, ^70^ ^]^ heterogeneous catalysis^[^ ^71^, ^72^ ^]^ electrocatalysis^[^ ^73^ ^]^ MOF^[^ ^74^ ^]^
X‐ray Photoelectron spectroscopy (XPS) Ambient Pressure XPS (AP‐XPS, NAP‐XPS, AP‐HAXPES)	0.15‐25 nm (50‐8000 eV)	Surface and interface (solid‐gas and solid‐liquid) science element‐specific chemical composition local bonding and structure site‐specific dynamics in solid, liquid, and gas phases and the interfaces formed between these phases	Photoemission	Surface‐sensitive technique: information depth of some tens of nm; Sensitive to all elements except for H and He; Difficulties in measuring insulating samples Requires UHV or near‐UHV conditions	s‐min	Catalysis electrochemistry adsorbate‐adsorbent interactions	Chemical state measurements on surfaces; Providing valuable in situ and operando information at solid‐gas, liquid‐gas and solid‐liquid interfaces; Huge variety of addressable sample systems including powders, liquids, gases, biological molecules; Investigation at elevated pressures under well controlled conditions (temperature, pressure, type of gas/liquid)	General^[^ ^75^, ^76^ ^]^
Ultraviolet/Visible (UV/Vis) absorption spectroscopy	200‐700 nm; (up to 1500 nm) (0.8 – 6 eV)	Electron and charge transfer transitions of transition metal ions and conjugated system Electronic transition in conjugated systems Excitation of metal‐charge‐transfer states Monitoring of oxidation/reduction reactions Information for both organic and inorganic compounds	Transmission for liquid and crystal, diffuse reflections for powder fiber‐optic DRS	Not well‐resolved bands Limited sensitivity highly absorbing systems like MOFs and COFs require dilution or thin film samples	ms ‐min	High temperature (<∼600 °C) and high‐pressure reaction can be measured by fiber‐optical DRS Probing excited‐state dynamics in catalytic processes	widely applicable in catalysis suitable for liquid and solid phase	General and catalysis^[^ ^42^, ^77^ ^]^ Fluorescence sensing^[^ ^7^, ^8^, ^17^ ^]^
Raman scattering	200‐1500 nm (0.8 – 6 eV)	Characterization in the low spectral range structure of metal oxide species lattice vibrations Host‐guest interactions Framework motion	Confocal setup Surface, Powder, Crystal, Solution Detection under 0°, 90° and 180°	Fluorescence background in spontaneous Raman scattering Sample heating and damage	ms – min	Catalysis, Material characterization	high spatial resolution, label‐free chemically sensitive good for monitoring molecular non‐centrosymmetric species	General in MOFs^[^ ^78^, ^79^ ^]^ Technical implementations^[^ ^80^, ^81^ ^]^ Band assignment^[^ ^39^ ^]^ reviews MOFs^[^ ^79^, ^82^, ^83^, ^84^, ^85^ ^]^
Infrared (IR) absorption spectroscopy	Mid‐IR: 2.5 – 10 µm (0.1 – 0.5 eV)	Molecular vibrations Identification/structure of (adsorbed) species, adsorbate–adsorbent interaction (binding modes, symmetry changes)	Transmission Attenuated total reflectance (ATR), Diffuse reflectance infrared Fourier transform spectroscopy (DRIFTS), Infrared reflection absorption spectroscopy (IRRAS or RAIRS) Step‐scan, rapid‐scan	Sensitivity Complicated band assignment for complex surface species	ns – min	Surface catalytic reactions Adsorbate studies Monitoring of chemical processes	widely applicable for functional group identification High versatility for operando studies	General^[^ ^50^ ^]^ Band assignment^[^ ^39^, ^81^ ^]^
Terahertz (THz) spectroscopy	0.1‐ 1 mm (2–30 meV)	information about the dynamic lattice host‐guest interactions phonon modes	Transmission, ATR	Low energy resolution	ms – min		Probes low‐frequency modes, valuable for studying dynamic processes like lattice vibrations	MOFs;^[^ ^54^ ^]^ time‐resolved THz^[^ ^53^ ^]^
Nuclear Magnetic Resonance (NMR) spectroscopy	1 m – 100 m (0.1 neV – 1200 neV) Radio waves	Chemical nature of framework/surface sites, adsorbates adsorbate–adsorbent interaction	Solid‐state, liquid‐state, MAS, HRMAS	High demand for preparation of sample, require special design for MAS rotor	ms – min	Gas adsorption and diffusion into reticular materials Solid‐state structure dynamics	Provides detailed molecular information Useful for solid‐state dynamics	General for MOFs,^[^ ^86^, ^87^ ^]^ general for nanoporous solids^[^ ^57^, ^88^ ^]^ MOF functionalization^[^ ^89^, ^90^ ^]^ Heterogenous catalysis^[^ ^91^ ^]^
Electron Paramagnetic Resonance (EPR) spectroscopy	1 cm – 100 cm (0.1 neV – 1200 neV) Microwaves	Structural and electronic properties of transition metal ions Coordination valence state	Commonly performed in solution state Solid‐state possible with cryoprobes	Requires paramagnetic species Limited to unpaired electrons Specialized interpretation	ms ‐ min	Catalysis, studying free radicals Detection of short‐lived reactive species	Highly sensitive to unpaired electrons, valuable for studying free radicals and metal complexes	general^[^ ^92^ ^]^ catalysis^[^ ^93^ ^]^

Abbreviations: t_R_, time resolution; s, second; min, minute.

Lastly, we will briefly explore the spatial resolution of these techniques and provide an overview of spectroscopy‐based microscopy techniques used in in situ and operando studies. These advanced methods combine the strengths of spectroscopy with the spatial resolution of microscopy, allowing for detailed analysis of materials at the micro^−^ and nanoscale. Such techniques are essential for examining the spatial distribution of elements and compounds within complex systems, providing insights into the structural and functional dynamics of materials under real‐world conditions. A summary of these techniques, along with their properties and relevant literature references, can be found in supporting Table S1 (Supporting Information).

### 2.1 Introduction into Spectroscopies across the Electromagnetic Spectrum

Spectroscopy, defined as the study of the interaction between electromagnetic radiation and matter as a function of wavelength or frequency, encompasses a wide range of techniques that probe atomic and molecular species. We sorted the spectroscopic techniques from shorter to longer wavelengths λ_0_ ranges and describe their interaction with species at the atomic and molecular level and involved phenomena (Figure 2C). For a more comprehensive understanding of each technique and its applications in the field of reticular materials, readers are encouraged to explore the detailed references provided in Table 1.

Mössbauer spectroscopy investigates the structural and electronic properties of atomic nuclei in solids by probing energy level changes using gamma radiation (λ_0_ = 1–100 pm) through the Mössbauer effect, during which gamma radiation is absorbed and emitted without any energy loss due to recoil. Mössbauer spectroscopy is most commonly used for elements that have an isotope capable of undergoing the Mössbauer effect. The most relevant elements used in MOFs include Fe, Co, Sn, Ru, and Ir, which are particularly important for studying redox processes, catalytic activity, and the detailed structural and electronic characteristics of these materials. Mössbauer spectroscopy provides detailed insights into the local environment and oxidation states of iron (and other Mössbauer active elements) within metal–organic frameworks (MOFs), such as MOF‐74(Fe). This technique can precisely differentiate between Fe(II) and Fe(III) oxidation states through isomer shifts and quadrupole splitting, and it provides information on the local magnetic environment via magnetic hyperfine splitting, which is crucial for understanding the electronic configuration and symmetry of iron sites. While XAS is effective for identifying oxidation states and coordination environments, Mössbauer spectroscopy adds a layer of precision in quantifying the magnetic properties and electronic environments of iron‐containing sites within MOFs and COFs.

X‐ray absorption spectroscopy (XAS) investigates the structural and electronic configuration of metal centers by exciting core‐level electrons using X‐ray photons, typically provided at synchrotron facilities in the energy range up to 100 keV. The technique provides two key pieces of information: X‐ray absorption near edge structure (XANES) reveals electronic structure and local geometry, while extended X‐ray absorption fine structure (EXAFS) delivers detailed structural data such as bond lengths, coordination numbers, and types of neighboring atoms. XAS is element‐specific, making it ideal for isolating active sites in multielement systems. For instance, during electrocatalytic CO_2_ conversion in HKUST‐1, in situ XAS revealed the coordination environment of the Cu clusters and that Cu(II) sites were reduced to metallic copper clusters.^[^ ^40^ ^]^ Unlike techniques requiring long‐range order, XAS can probe local atomic environments, and its high penetration depth of hard X‐rays (>4 keV) allows for flexible in situ and operando studies during material formation and catalysis.

X‐ray photoelectron spectroscopy (XPS) analyzes the electronic structure and chemical composition of solid surfaces by measuring the kinetic energy of photoelectrons ejected from a sample upon irradiation with X‐rays. Conventional XPS is typically performed under ultra‐high vacuum conditions, which limits its use for in situ or operando studies. However, advancements in XPS technology have led to the development of ambient pressure XPS (AP‐XPS or NAP‐XPS), which enables surface characterization in gaseous or liquid environments at pressures up to the mbar range. For instance, operando NAP‐XPS found that the metallic Pt phase in UiO‐67, rather than the oxidic phase, is the active site during CO oxidation between 100 and 300 °C.^[^ ^41^ ^]^ In contrast to XAS that provides bulk information, XPS is a surface sensitive technique (1–10 nm) and therefore particularly suitable for thin film analysis or photochemistry.

UV–vis absorption spectroscopy historically covered the ultraviolet and visible, regions, corresponding to wavelengths between 200 and 750 nm.^[^ ^42^ ^]^ However, it is also commonly used to probe transitions in the near‐infrared (NIR) region, extending up to 1500 nm. This extended range includes the energies necessary for electronic excitations in molecules and materials, e.g. used for energy harvesting. Notable electronic transitions include n → π* and π → π* transitions in organic molecules, d‐d transitions, ligand‐to‐metal charge transfer (LMCT), and metal‐to‐ligand charge transfer (MLCT) transitions in transition metal complexes. Additionally, UV–vis absorption spectroscopy can also be used to study transitions in the electronic shells of rare earth metals or the band gaps of semiconductors. Additionally, the technique is effective in studying structural defects in materials by observing changes in electronic transition energies. For example, UV–vis absorption spectroscopy is invaluable in spectroelectrochemistry for studying coordination environments and redox processes,^[^ ^43^ ^]^ as well as for monitoring structural changes such as *trans*‐to‐*cis* isomerizations in photoresponsive materials.^[^ ^44^ ^]^

Luminescence, including fluorescence and phosphorescence, involves light emission following electronic excitations and is widely used in spectroscopy and microscopy to study chemical reactions and material interactions. The key characteristics of fluorescence include its high sensitivity, selectivity, and rapid response, which make it particularly useful in studying dynamic processes within reticular materials. In the context of reticular materials, fluorescence spectroscopy can be employed to track the diffusion of guest molecules,^[^ ^45^ ^]^ monitor the loading and release of drugs in pharmaceutical applications,^[^ ^6^, ^22^, ^46^, ^47^ ^]^ and observe the environmental responses of the framework.^[^ ^48^ ^]^ It provides detailed insights into the interactions between guest molecules and the host framework by analyzing changes in fluorescence intensity, wavelength, and lifetime. Phosphorescence, unlike fluorescence, involves a slower light emission due to intersystem crossing to a triplet state. It offers additional information about long‐lived excited states, which are particularly interesting for photochemical applications.

Infrared (IR) absorption and Raman spectroscopy probe the vibrational properties of molecules and provide complementary insights into molecular structures and compositions.^[^ ^49^, ^50^, ^51^, ^52^ ^]^ Both are widely used for monitoring chemical processes during synthesis, functionalization and catalysis and instrumental in analyzing structural dynamics, reaction intermediates and guest‐host interactions with MOF and COFs. IR spectroscopy measures the absorption of infrared radiation, revealing information about different vibrational modes associated with specific bond vibrations and functional groups such as metal‐ligand interactions in MOFs or covalent linkages in COFs. Raman spectroscopy complements this by using inelastic scattering of light to probe vibrational, rotational, and other low‐frequency modes. Raman is particularly advantageous for studying non‐polar molecules and symmetric vibrations, which may be weak or inactive in IR spectroscopy.

Terahertz (THz) spectroscopy, also referred to as far‐infrared (FIR) spectroscopy, probes long‐range, low‐energy dynamic modes in the energy range of 2–30 meV.^[^ ^53^, ^54^ ^]^ Unlike mid‐IR spectroscopy, which focuses on local bond vibrations (bond lengths or angles), THz spectroscopy captures the dynamic structure and bulk motions within materials. THz time‐domain spectroscopy (THz‐TDS) is particularly sensitive to structural changes caused by chirality and is more effective at measuring bulk motions as opposed to localized vibrations. This sensitivity to low‐energy modes makes THz spectroscopy ideal for studying the overall dynamic behavior of MOFs, including how they respond to external stimuli and their assembly processes. Researchers have utilized THz spectroscopy to directly measure the conductivity and photoconductivity of MOFs for photovoltaic applications, providing valuable insights into their potential for solar energy conversion and other advanced technological applications.^[^ ^54^ ^]^

Electron paramagnetic resonance (EPR) spectroscopy, also known as Electron Spin Resonance (ESR) spectroscopy, is a technique to study materials with unpaired electrons using microwaves to excite electron spins in a magnetic field. The resulting EPR spectrum provides information on the so‐called g‐factor, hyperfine splitting, and zero‐field splitting, which provides insights into the local electronic environment, the nature of the chemical bonds, and the interactions between electron spins and nearby nuclear spins. EPR spectroscopy is particularly useful in studying transition metal complexes, free radicals, and defects in reticular materials. For instance, phase transitions at the Cu‐paddle wheel unit of the breathing MOF DUT‐49(Cu) were monitored during adsorption experiments,^[^ ^55^ ^]^ and the formation of radicals during illumination of photoactive COFs has been tracked with in situ EPR spectroscopy.^[^ ^56^ ^]^

Nuclear magnetic resonance (NMR) spectroscopy probes the local magnetic environments of atomic nuclei, providing detailed information about the chemical structure, dynamics, and interactions of molecules.^[^ ^57^ ^]^ In liquid‐state NMR, well‐resolved spectra of small molecules in isotropic solvents allow the characterization of organic linkers during MOF formation and in situ reaction monitoring. For example, ^2^⁷Al NMR has been valuable in identifying pre‐nucleation species during the formation of Al‐based MOFs like MIL‐100(Al).^[^ ^58^ ^]^ In contrast, solid‐state NMR, using techniques like magic angle spinning (MAS‐SS‐NMR), enabled structural analysis of the flexible MOF DUT‐8(Ni) of the coordination of guest molecules at the nickel paddle‐wheel during nitrogen sorption.^[^ ^59^ ^]^

Electrochemical Impedance (EIS) spectroscopy is a technique used to characterize the electrochemical properties of materials, such as battery materials, fuel cells, or electrochemical sensors, by measuring the impedance across a range of frequencies.^[^ ^60^, ^61^ ^]^ It involves applying a small alternating current (AC) voltage to an electrochemical cell and measuring the resulting current to probe processes occurring at different time scales^.^ The impedance, a complex number with real (resistance) and imaginary (reactance) components, provides insights into energy dissipation and storage within the system. The data obtained from EIS can be modeled using equivalent electrical circuits. These models help in understanding the kinetics and mechanisms of the electrochemical reactions and transport processes within the cell and allow for studying, e.g., proton or ion conductivity in reticular materials.^[^ ^16^, ^19^, ^25^, ^62^ ^]^

Mass spectrometry (MS) is a powerful analytical technique used to determine the mass‐to‐charge ratio (*m*/*z*) of ions, providing precise molecular weight, structural information, and composition of compounds. Unlike spectroscopies mentioned above that are based on energy‐resolved interaction, MS relies on the ionization of molecules followed by their separation and detection in a mass analyzer. MS can analyze both the individual components and the complex interactions within the material, offering valuable insights into the compositions of MOFs and COFs. This method complements traditional imaging techniques, such as transmission electron microscopy (TEM), by offering fast, quantitative mass data. Techniques such as secondary ion mass spectrometry (SIMS) and time‐of‐flight SIMS (ToF‐SIMS) have been implemented to study the surface composition and fragmentation patterns of MOFs during formation and growth^[^ ^63^ ^]^ or sorption depth profiles.^[^ ^64^ ^]^ Recently, charge detection quadrupole ion trap mass spectrometry (CD‐QIT MS) has emerged as an advanced method for mass measurements of ZIF‐8 and enzyme‐encapsulated ZIF‐8 (GOx@ZIF‐8),^[^ ^65^ ^]^ providing valuable insights into particle size, surface modifications, and the encapsulation content of guest molecules.

### 2.2 Introduction to Spatially High‐Resolution Techniques

Many of the above‐mentioned spectroscopic methods (Table 1) have been tailored to achieve the desired temporal resolution for monitoring chemical reactions in situ and operando. Besides temporal evolution, their spectroscopic signatures serve as image contrast in microscopy and nanoscopy, each offering different spatial resolutions (Table S1, Supporting Information). Microscopy refers to the suite of techniques that allow for the visualization of structures that are too small to be seen with the naked eye. Nanoscopy, a sub‐field of microscopy, refers to imaging techniques with nanometer spatial resolution. The following section gives a short overview of available techniques for probing reactions spatially resolved on the macroscopic, microscopic, and atomic levels.

#### 2.2.1 Optical Microscopy

One of the key advantages of light microscopy is its non‐invasive nature, which allows researchers to study even live samples and dynamic processes without causing damage or altering the investigated material. Traditional light microscopy, with its resolution limited by the wavelength of light, involves three types of microscopy schemes: confocal, widefield, and light‐sheet systems. Confocal microscopy^,^ as frequently implemented for fluorescence‐, IR‐ and Raman‐based systems, improves optical resolution and contrast by using a spatial pinhole to block out‐of‐focus light. In the visible, this approach achieves spatial resolutions down to ≈200–250 nm depending on the employed excitation wavelength and time resolution of ns^.^ In combination with time‐correlated single‐photon counting (TCSPC) technology^,^ fluorescence microscopy can monitor, e.g., the lifetime of ligand‐to‐metal charge transfer (LMCT^)^ reactions in MOFs or (photo)catalytical reactions.^[^ ^15^ ^]^ Wide‐field microscopes are at the heart of super‐resolution techniques such as single‐molecule localization microscopy (SMLM) or interferometric scattering (iSCAT) microscopy, which allows for monitoring, e.g., the synthesis of COFs in situ.^[^ ^94^ ^]^ They provide faster imaging by illuminating the entire sample at once, making it suitable for monitoring dynamic processes with 2D read‐out at a cost of lower lateral and temporal resolution (>≈250 nm; >ms). Additionally, wide field techniques also include dark‐field microscopy, which is particularly useful for enhancing contrast in unstained samples for detection nanoparticles and small features by illuminating the sample with oblique light. Lattice light‐sheet microscopes exhibit high speed and low phototoxicity, allowing for the 3D imaging, e.g., of MOF nanoparticles and their interaction in live cells and tissues with subcellular resolution^.^

#### 2.2.2 Super‐Resolved Light Microscopy

Overcoming the diffraction limit in light microscopy to access structures <200 nm has been a significant challenge in the quest to visualize materials at the nanoscale in a noninvasive manner. Several advanced strategies have been developed to surpass this barrier and are outlined below^.^ More in‐depth references to excellent overview articles describing high^−^resolution methods in detail are included in Table S1.

The first strategy is based on temporally controlling the fluorescent emission of neighboring chromophores to localize single emitters. Single‐Molecule Localization Microscopy (SMLM), including techniques like Stochastic Optical Reconstruction Microscopy (STORM) and Photoactivated Localization Microscopy (PALM), achieve resolutions as fine as 10–20 nm by precisely localizing individual fluorescent molecules within a sample. A particularly powerful approach for super‐resolution imaging, Nanometer Accuracy by Stochastic Catalytic Reactions (NASCA) Microscopy, uses fluorogenic catalytic reactions in porous systems to map catalytic sites with nanometer precision. This approach has been used to study ZIF‐8 and Ba_2_(btc)(NO₃)MOFs (btc = benzene‐1,3,5‐tricarboxylate), revealing catalytic site distribution and diffusion pathways within nanopores.^[^ ^95^, ^96^ ^]^

**Figure 3:**
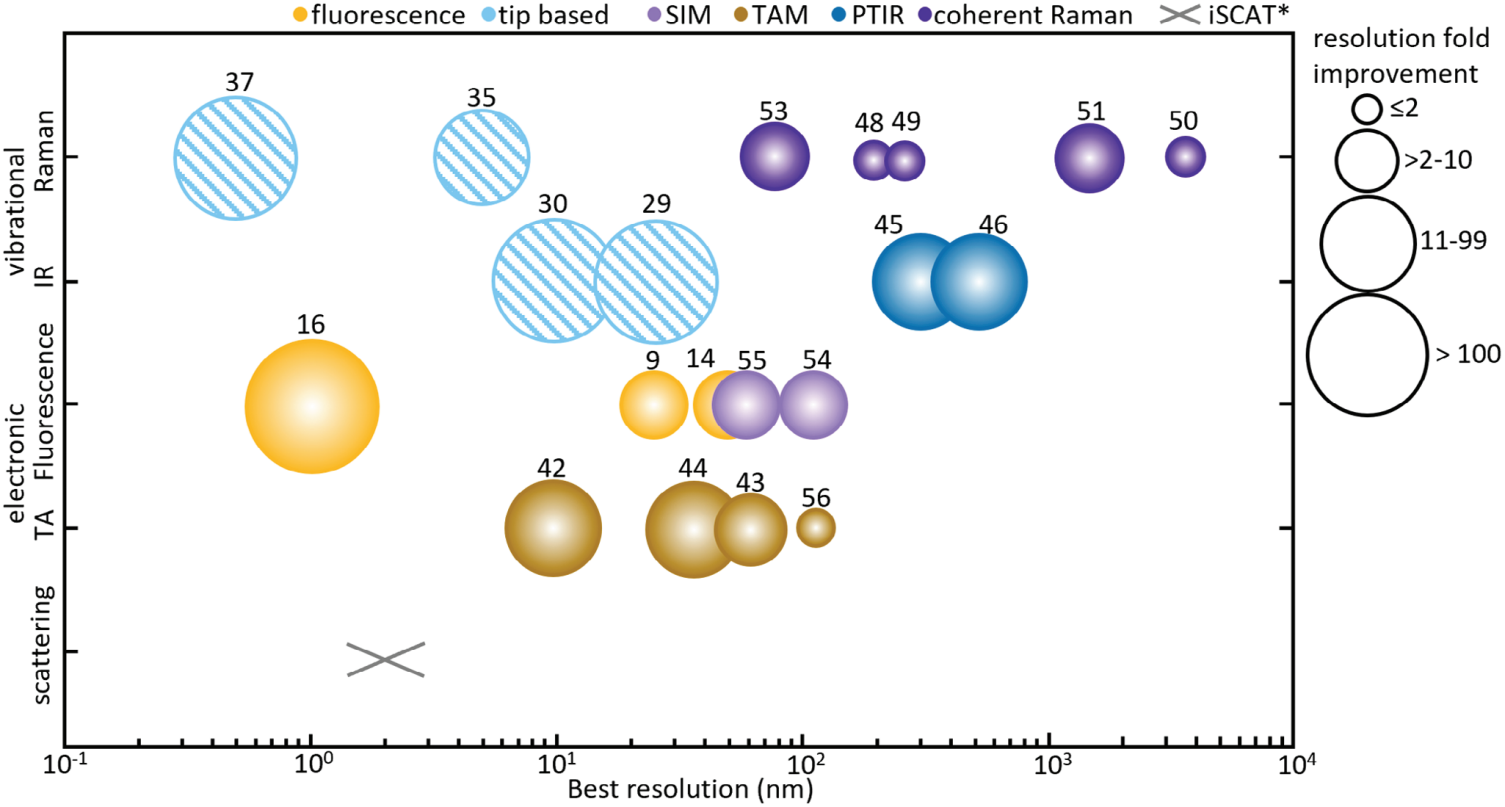
Label‐free optical super‐resolution imaging techniques. Spatial resolution below the diffraction‐barrier can be achieved for all optical spectroscopy techniques. In the absence of plasmonic enhancement, the highest resolution can be achieved for methods probing electronic transitions rather than vibrational ones. The numbers shown in the plot correspond to the reported values of spatial resolution achieved by the various techniques. Reproduced with permission.^[^ ^102^ ^]^ Copyright 2020, Annual Reviews. *Spatial resolution for iSCAT taken from ref. [103].

Another approach circumvents the diffraction barrier by using nonlinear excitation schemes or shaping the excitation profiles. Transient Absorption Microscopy (TAM) and the fluorescence‐based technique Stimulated Emission Depletion (STED) Microscopy achieve 40–60 nm and 20–30 nm resolution, respectively.^[^ ^97^, ^98^ ^]^ While STED is frequently applied in biological research, its ability to resolve fine structural details in 3D for imaging reticular materials remains largely untapped. It could be particularly useful for investigating the distribution of nanoparticles in cells‐MOF/COF composite materials. Similarly, Structured Illumination Microscopy (SIM) enhances resolution by using patterned light illumination and computational reconstruction. SIM has already been used to track molecules and active sites in zeolites,^[^ ^99^ ^]^ and UiO‐66 nanoparticle uptake into MCF‐7 cells.^[^ ^100^ ^]^ Both STED‐ and SIM‐type implementations have been implemented for super‐resolved Raman‐based imaging (**Figure** **3**).^[^ ^101^ ^]^

In addition to fluorescence‐based super‐resolution techniques, several nano‐imaging methods leverage the nanoscale tip present in Atomic‐force Microscopy (AFM) and Scanning Tunneling Microscopy (STM) to drastically improve the resolution of conventional optical microscopy or spectroscopy techniques (Figure 3). These techniques provide atomic‐scale topographical and electronic property maps. AFM has been used, for instance, to study the surface roughness and mechanical properties of COF thin films,^[^ ^104^ ^]^ while STM has enabled visualization of the electronic states and structural details of individual honeycomb MOF layers at the nanoscale.^[^ ^105^ ^]^

These label‐free super‐resolution techniques are mostly based on Transient Absorption (TA), IR absorption, and Raman scattering techniques.^[^ ^102^ ^]^ Specifically, IR absorption has been used for reticular materials. It can be implemented, e.g., as IR‐scattering‐type Scanning Near‐Field Optical Microscopy (IR s‐SNOM) and Atomic‐Force Microscope‐based IR spectroscopy (AFM‐IR, which is Photothermal Induced Resonance (PTIR) in the IR spectral range; also known as nano‐IR spectroscopy) to provide chemical information with nano‐scale spatial resolution (Figure 3).^[^ ^106^, ^107^ ^]^ While facet‐dependent adsorption studies on ZIF‐8 and MOF thin film formation have been reported for nano‐IR spectroscopy,^[^ ^108^, ^109^ ^]^ its application for operando applications in the field of reticular chemistry is still pending.^[^ ^110^ ^]^ Both methods are optimal for monitoring the chemical composition and topology of surface features, films, as well as catalytic reactions. However, they have difficulties in resolving anything below the surface.

#### 2.2.3 Electron Microscopy Techniques

Electron microscopy offers significantly higher spatial resolution compared to optical microscopy, making it essential for atomic‐level imaging. Scanning Electron Microscopy (SEM) provides detailed surface images with spatial resolutions down to a few nanometers, while Transmission Electron Microscopy (TEM) reveals atomic structures and crystalline defects. Scanning Transmission Electron Microscopy (STEM) combines SEM and TEM for high‐resolution imaging and compositional analysis. Integrated techniques like Electron Energy Loss spectroscopy (EELS), when combined with STEM, offer high‐resolution chemical and electronic information. For example, in UiO‐66(Zr), EELS was used to analyze the Zr M‐edge, revealing the oxidation state and coordination environment of zirconium, while the oxygen K‐edge distinguished between different oxygen species. This provided critical insights into the defects, stability and their catalytic potential at the nanoscale. By combining liquid‐phase TEM, Cryo‐EM, high‐angular dark‐field STEM and electron ptychography it was even possible to follow the growth process of SURMOFs in solution at cluster scale.^[^ ^111^ ^]^

#### 2.2.4 X‐Ray‐Based Microscopy Techniques

X‐ray microscopes, i.e., the combination of XAS with microscopy, including Scanning Transmission X‐ray Microscopes (STXM) and Transmission X‐ray Microscopes (TXM),^[^ ^112^ ^]^ can achieve spatial resolutions down to 20–30 nm, enabling detailed chemical mapping and high‐resolution imaging of materials. These techniques are particularly powerful for studying heterogeneous materials and complex systems at the nanoscale. Advanced STXM techniques, such as ptychography and STXM‐Total Electron Yield (STXM‐TEY) imaging have the potential for operando analysis of materials for energy storage and catalysis.^[^ ^113^ ^]^ X‐ray Photoelectron Spectroscopy (XPS) and Near‐Ambient Pressure XPS (NAP‐XPS) provide spatial resolution in the range of tens of µm, typically around 10–50 µm, limited by the spot size of the X‐ray beam. When combined with scanning techniques, XPS allows for spatially resolved surface chemical analysis. NAP‐XPS extends this capability to near‐ambient pressure conditions, suitable for studying surfaces under realistic environmental conditions with micrometer‐scale resolution, allowing for investigating dynamic processes such as gas sorption, catalysis, and surface reactions in situ. Hard X‐ray Photoelectron Spectroscopy (HAXPES), an extension of conventional XPS, uses a higher spatial resolution of a few micrometers. In situ X‐ray Absorption Fine Structure Spectroscopy combined with Computed Tomography (XAFS‐CT) recently allowed for mapping water‐coordinated Co sites in MOF‐74(Co) crystals with 1 µm spatial resolution under several water vapor pressures.^[^ ^114^, ^115^ ^]^ Near‐Edge XAFS (NEXAFS) spectroscopy provides detailed information about the electronic structure and bonding environment with spatial resolution typically in the range of tens of nanometers when combined with STXM.

#### 2.2.5 Probing Spin States with High Spatial Resolution

While conventional Electron Paramagnetic Resonance (EPR) and Nuclear Magnetic Resonance (NMR) spectroscopy offer limited spatial resolution, advances in EPR imaging^[^ ^116^ ^]^ and micro‐imaging NMR^[^ ^117^ ^]^ have pushed these techniques into the micrometer and nanometer scales, allowing for detailed studies of magnetic and chemical environments. Terahertz (THz) spectroscopy provides spatial resolutions on the order of hundreds of µm due to the longer wavelengths of THz radiation.^[^ ^95^ ^]^ Techniques like near‐field THz microscopy can enhance spatial resolution to the micrometer scale, enabling detailed studies of material properties at THz frequencies.^[^ ^118^ ^]^ Mössbauer spectroscopy typically operates with spatial resolutions on the millimeter scale due to the nature of the gamma‐ray beam. However, synchrotron‐based Mössbauer spectroscopy can achieve better spatial resolution, down to tens of µm, for localized studies of electronic and magnetic properties.^[^ ^119^ ^]^

## 3 State‐of‐the‐Art Operando and In Situ Spectroscopy for Reticular Materials

**Figure 4:**
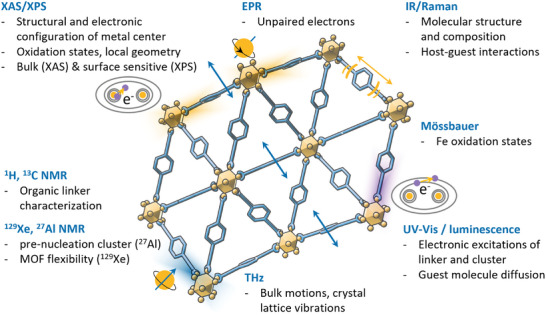
In situ/operando spectroscopic techniques used to study reticular materials.

This chapter discusses how in situ and operando spectroscopy techniques are employed to investigate the formation and functionalization of reticular materials. We will explore how these methods reveal structural dynamics induced by external stimuli such as pressure, temperature, light, and the introduction of guest molecules. A critical aspect of reticular materials is the formation of defects, which can influence their catalytic activity, stability and overall performance. We close this chapter by examining the behavior of reticular materials in catalytic processes, like single‐site catalysis, photocatalysis, and electrocatalysis, highlighting how in situ and operando spectroscopy contribute to our understanding of these complex mechanisms (**Figure** **4**).

### 3.1 Formation of Reticular Materials

**Figure 5:**
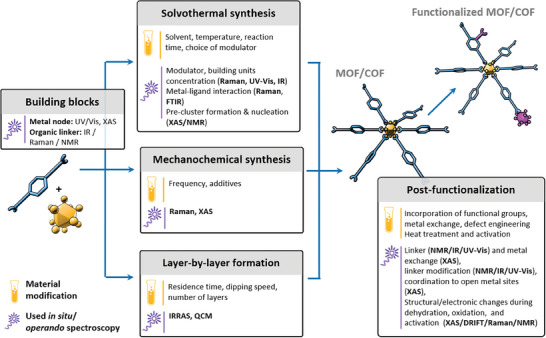
Different synthetic methods and post‐functionalization of reticular materials with corresponding spectroscopic techniques used to monitor various stages.

The synthesis of reticular materials is a complex process that involves multiple reaction partners—such as linkers, metal‐containing salts, solvents, and modulators—under varying parameters like concentration, temperature, and time (**Figure** **5**). The outcomes of these syntheses are often challenging to predict, leading to the need for large‐scale screening studies. While these studies have uncovered new reticular materials and identified key synthesis parameters, they are typically limited to ex situ analysis. This limitation makes it difficult to study intermediates, and understand the formation and crystallization mechanisms, making it challenging to derive generalizable conclusions. In situ studies have become increasingly important in this area,^[^ ^120^, ^121^ ^]^ as they offer real‐time insights into the nucleation and growth processes of reticular materials. These studies have revealed the roles of mediators and different intermediate pre‐nucleation building unit (PNBU) species,^[^ ^58^, ^86^, ^90^, ^122^, ^123^ ^]^ providing a deeper understanding of the synthesis process.

Over the past decades, significant advancements have been made in understanding the nucleation process of MOFs, driven by the application of particle‐sensitive characterization tools.^[^ ^121^ ^]^ Techniques such as in situ small‐angle and Wide‐Angle X‐ray Scattering (SAXS‐WAXS), light scattering, and Transmission Electron Microscopy (TEM) have enriched our understanding of MOF particle nucleation.^[^ ^121^, ^124^, ^125^, ^126^ ^]^ These methods have provided valuable insights into the early stages of particle formation, although the underlying molecular mechanisms governing the assembly of seeds and pre‐nuclei and the subsequent formation of MOF particles remain largely unknown. X‐ray scattering techniques, which are sensitive to both crystalline and, in some cases, amorphous phases, have been extensively used for monitoring these processes.^[^ ^121^ ^]^ However, due to the complexity and variability in probed sizes, no single technique can fully capture the evolution of all species involved across the diverse timescales ranging from seconds to days during the synthesis. As a result, a combination of in situ methods is often required to build a complete picture of the crystallization process, spanning multiple length scales and capturing the dynamics of crystal growth.

In addition to the assembly and disassembly process, post‐synthetic treatments and functionalization are critical in fine‐tuning the properties of reticular materials for specific applications. These processes, which include the incorporation of functional groups, metal exchange, and defect engineering, can significantly alter the material's properties. In situ characterization is equally important in these stages, as it provides insights into the chemical environment and structural changes during post‐synthetic modifications.

The following examples illustrate the significance of understanding both the local environment of species at different synthesis stages and the crystal growth over various length scales.

#### 3.1.1 Nucleation and Growth

When reviewing the literature, we found that in situ studies on the nucleation and growth of reticular materials can be broadly divided into two categories: I) Studies that probe changes in the metal node during material formation, particularly in MOFs. These studies predominantly use X‐ray‐based techniques, such as XAS, to monitor the structural and electronic changes in the metal nodes as the material forms. A significant advantage of XAS is that it does not require long‐range order, unlike conventional diffraction techniques. This makes XAS particularly valuable for studying metal nodes during the early stages of nucleation and growth, when the material may not yet have developed long‐range crystalline order. II) Studies focusing on changes in the linker or mediator species. Here, vibrational spectroscopy is especially powerful for tracking the evolution of these species during the synthesis process. In the following, we will explore key studies that track the progression of prenucleation and cluster formation to the crystallization of reticular materials, highlighting how in situ techniques have deepened our understand of these critical processes.

##### Prenucleation and Cluster Formation

The formation of MIL‐53(Al) was studied using simultaneous FTIR and Raman spectroscopy, coupled with turbidity measurements.^[^ ^122^ ^]^ These techniques revealed the presence of pre‐nucleation building units (PNBUs), consisting of one aluminum atom coordinated with a single linker molecule. The study further tracked the assembly of these PNBUs ([Al(H_2_O)_4_–Hbdc]^2^, bdc = 1,4‐benzene dicarboxylate) into MIL‐53 nuclei and identified the decomposition of DMF, which played a role in the MOF's final crystallization. The same combination of vibrational spectroscopies was used to investigate the formation of MIL‐68(Al) and MIL‐53(Al) and the role of formic acid as a modulator.^[^ ^127^ ^]^ The presence of formic acid was found to significantly alter the kinetics of MOF formation, reducing the number of pre‐nucleation clusters and favoring the formation of specific isomers. In situ^[^^2^⁷^]^ Al NMR was extensively used to study the synthesis of Al‐based MOFs, such as MIL‐96, MIL‐100, and MIL‐110.^[^ ^58^ ^]^ A study by Haouas et al. identified multiple Al‐based complexes in the solution during the hydrothermal synthesis. The research identified the hexa‐aquo Al(H_2_O)_6_^3+^ complex as the predominant species throughout the synthesis. Other species, such as the 1:1 Al‐btc complex (btc = benzene‐1,3,5‐tricarboxylate), and the Al_2_ dimers bridged by btc ligands, were observed at different stages of the reaction.^[^ ^58^ ^]^

Low‐frequency Raman spectroscopy was used to track MOF formation in water, specifically observing the consumption of the modulator nitric acid and of the ligand during crystallization of MIL‐140A(Ce) and UiO‐66(Ce).^[^ ^128^ ^]^ The ability to measure these changes non‐invasively through a glass vial adds a practical aspect to the monitoring of MOF synthesis in different environments. Resonance Raman (RR) spectroscopy was employed to monitor the interactions between cobalt(II) nitrate and 4,4′‐bipyridine (bpy) during the formation of a Co‐bpy‐based MOF in solution. The coordination of bpy with Co(II) ions was observed to shift the vibrational bands of the ligand, providing real‐time insight into metal‐ligand interactions as the MOF crystallized.^[^ ^123^ ^]^ During the formation of HKUST‐1 from a ZnO precursor layer, in situ ATR‐FTIR spectroscopy detected the initial presence of nitrate bands upon the introduction of Cu(NO₃)_2_. As the btc linker was subsequently flushed into the system, the appearance of carboxylate groups and a C═C band was noted, alongside the diminishing nitrate signals. This progression indicates the key steps of anion exchange and the successful incorporation of the linker into the forming MOF structure.^[^ ^129^ ^]^

##### Amorphous intermediates and crystallization

MOF formation often involves the presence of amorphous intermediates that subsequently dissolve and crystallize into the final structure. This process was observed for Fe‐btc,^[^ ^130^ ^]^ and MIL‐89(Fe),^[^ ^131^ ^]^ although ex situ, where amorphous phases were key transitional states before crystallization. Here, in situ studies could significantly advance our understanding by providing real‐time insights into these critical stages. Sushkevich and co‐workers, for instance, found that nucleation and growth can occur at significantly different rates, with nucleation often being faster than growth, as seen in UiO‐66 synthesis.^[^ ^132^ ^]^ Through in situ XAS, they could determine the structure of the Zr atoms in the molecular precursor and the secondary‐building units of UiO‐66, while MAS‐SS‐NMR revealed the transformation of the terephthalate linker and monitored the evolution of water and HCl signals. This combination of techniques unveiled the hydrolysis of zirconium chloroterephthalates as the key mechanism behind nucleation, offering crucial insights into controlling crystal size and material quality.

In another study, the synthesis of MOF‐74(Zn) was explored using a combination of in situ ATR‐IR spectroscopy and high‐energy XRD, which revealed that the kinetics of nucleation and growth during MOF synthesis strongly depend on the nature of the zinc salt and reaction temperature.^[^ ^133^ ^]^ These insights allowed researchers to optimize the synthesis process, resulting in faster synthesis and improved textural properties.

NMR spectroscopy has also provided valuable insights into the formation mechanisms of MOFs, particularly regarding the behavior and interactions of linkers and metal centers during synthesis and the transition from dissolved species to crystalline structures.^[^ ^86^ ^]^ In the synthesis of the framework MFM‐500(Ni), in situ ¹H NMR was employed to track the time evolution of the reaction and crystallization process. The study provided both qualitative information on solution‐phase processes and quantitative data on the kinetics of crystallization, including the determination of activation energies for nucleation and growth. Complementary ex situ SAXS and in situ PXRD further confirmed that the formation of cylindrical linker‐based aggregates precedes the appearance of the crystalline MFM‐500(Ni) phase.^[^ ^134^ ^]^

In situ Raman spectroscopy combined with XRD was employed to monitor the mechanochemical synthesis of ZIF‐8.^[^ ^135^ ^]^ Raman spectroscopy indicated the formation of non‐crystalline intermediate co‐crystals, while XRD tracked the gradual transition from reactants to MOF.

##### Valence State of Metal Node During Framework Formation

In situ techniques have proven critical for understanding the valence state dynamics of metal nodes during the formation of MOFs. For example, the synthesis of a Mn‐based MIL‐100 analog demonstrated that in situ oxidation of Mn(II) to Mn(III) was crucial for the formation of the framework, with the crystallization process occurring rapidly and involving multiple stages.^[^ ^136^ ^]^ Beyond NMR and X‐ray‐based techniques, in situ UV–vis spectroscopy has provided valuable information about valence states and incorporated metal ions. For instance, Lee et al. employed a combination of UV–vis absorption spectroscopy and inductively coupled plasma atomic emission spectroscopy (ICP‐AES) to quantitatively analyze the metal exchange rates during the formation of zinc–copper (Zntpo–CuHtpoO) and terbium–copper (Tbtpo–CuHtpo) MOFs (H_3_tpo = tris‐(4‐carboxylphenyl)phosphineoxide), which originated from a common Cu‐based MOF precursor (CuHtpo).^[^ ^137^ ^]^ Similarly, Zhang et al. combined synchrotron XAS with XRD to study the transformation of (Zn,Co) hydroxy double salts into mixed‐metal ZIF‐8. Their time‐resolved Zn K‐edge XANES spectra revealed changes in the coordination environment but not in the oxidation state, implying that there is no redox chemistry involved in ZIF‐8 formation.^[^ ^126^ ^]^ Beyer and co‐workers used in situ XANES with dispersive XAS setup to study the coordination environment in the early stages of ZIF‐8 crystallization in liquid phase.^[^ ^138^ ^]^ Linear combination analysis identified tetrakis(1‐methylimidazole)zinc(II) as a key intermediate.

For MOFs with metal ions like lanthanides, luminescence measurements offer an additional handle to study the formation process. Wen et al. monitored the plasma‐assisted formation of lanthanide metal–organic frameworks by recording their time‐dependent fluorescence emission.^[^ ^139^ ^]^ In another example, in situ fluorescence measurements were used to track the coordination of lanthanide ions during post‐treatment of 3D‐printed Ln^−^MOFs in response to small molecules, providing valuable insights into framework assembly under external control.^[^ ^140^ ^]^ In efforts to influence the crystal shape, HKUST‐1 was crystallized within parallel arrays of µm‐sized confined channels, forming highly oriented, high aspect ratio crystals. Characterization through porosity, elemental mapping, and fluorescence microscopy revealed well‐defined, large crystal domains, ideal for studying nucleation, growth, and diffusion kinetics.^[^ ^141^ ^]^

##### Polymorphism and Phase‐Purity

Polymorphism and phase purity are critical in reticular materials as different polymorphs can lead to distinct properties. Advanced spatially resolved techniques like fluorescence lifetime imaging (FLIM) combined with confocal fluorescence microscopy (CFM) have proven effective in identifying and quantifying phase heterogeneity in MOFs. For example, in NU‐1000 and NU‐901, these methods detected variations in fluorescence linked to local structural differences, providing a non‐destructive way to assess phase purity.^[^ ^142^ ^]^ Electron Nanobeam Diffraction and Pair Distribution Function (ePDF) analyses have further advanced our understanding of MOFs like Fe‐btc, revealing the coexistence of crystalline and amorphous regions within the material. These techniques have shown that, while certain coordination geometries are preserved, the crystalline phases exhibit significant disorder and reduced unit cell sizes, refining our structural models and identifying functional components.^[^ ^143^ ^]^

Besides diffraction and spatially‐resolved techniques, traditional spectroscopic methods, such as IR and Raman spectroscopy, are also valuable for monitoring polymorphism and phase purity, as shown ex situ so far.^[^ ^79^, ^143^, ^144^ ^]^

##### Role of the Solvent

The solvent environment and modulators play critical roles in directing the formation pathways of MOFs. For instance, acetone was found to influence the oxidation state of iron in the formation of MIL‐45(Fe) versus MIL‐100(Fe), and consecutively the crystal topology and growth kinetics, highlighting how solvents can act as structure‐directing agents.^[^ ^145^ ^]^ Similarly, the use of diethylamine in ZIF‐7 synthesis demonstrated how modulators can indirectly affect the crystallization process by neutralizing byproducts rather than directly coordinating with metal ions.^[^ ^146^ ^]^ Here, in situ techniques allow for real‐time monitoring of the early stages of MOF assembly while probing the impact of solvent interactions. A recent work combining time‐ and laterally‐resolved XAFS analysis is a great example of how advanced in situ experimental setups have enabled more precise and time‐efficient monitoring of fast reaction processes in reticular materials. These advancements allowed researchers to investigate the early stages of ZIF‐8 nucleation and the role of intermediate tetrakis(1‐methylimidazole)Zn(II) species.^[^ ^147^ ^]^ In situ NMR studies, such as those performed by Goesten et al., have demonstrated the role of solvents like DMF in promoting the formation of MOFs such as NH_2_‐MIL‐101(Al). Using a combination of ^1^H and ^27^Al NMR, the researchers were able to monitor the evolution of the synthesis process in real‐time, revealing how DMF facilitates the coordination of the metal center with the organic linker.^[^ ^148^ ^]^

##### MOF Thin Films

Thin film MOFs, including Surface‐mounted Metal‐Organic Frameworks (SURMOFs), present unique challenges in characterization due to their low thickness and relatively low sample volume, which demands for more specialized in situ techniques. In situ atomic force microscopy (AFM) combined with infrared nano‐spectroscopy was employed to study the formation of SURMOFs like HKUST‐1. The study revealed the growth of larger MOF islands surrounded by a thin “carpet” layer, both of which were influenced by the synthesis temperature.^[^ ^109^ ^]^ Additionally, in situ IRRAS (Infrared Reflection Absorption Spectroscopy) has been instrumental in studying layer‐by‐layer synthesis of the SURMOF [Cu_2_(F_4_bdc)_2_(dabco)] (F_4_bdc = tetrafluorobenzene‐1,4‐dicarboxylate and dabco = 1,4‐diazabicyclo‐[2.2.2]octane), providing structural information about the orientation of carboxylate bands in copper paddlewheel structures.^[^ ^149^ ^]^ This technique was particularly useful for understanding the sequential assembly and structural evolution of MOFs in a controlled synthesis environment. Similarly, the polarization of light can be used to study the orientation of MOF thin films deposited on ATR crystals in conventional FTIR spectroscopy. Thereby, the orientation of MOF linkers as well as the alignment of adsorbing guest has been studied.^[^ ^150^, ^151^ ^]^

##### Polymerization of COFs

Unlike MOFs, COF formation often involves rapid polymerizations, dynamic rearrangements, and stacking processes that occur on very short timescales, making in situ monitoring and capturing intermediate species more difficult. Dichtel et al. were the first to monitor COF formation using turbidity measurements, tracking the process as dissolved monomers precipitated and began scattering light at a wavelength of 600 nm. They monitored the turbidity during the synthesis of COF‐5, a prototypical 2D hexagonal framework. They found that the polymerization process includes at least one irreversible step and that the crystallite size can be controlled by adjusting the water content. This study provided the first quantitative measurement of COF‐5 formation rates and demonstrated that reaction conditions significantly influence the crystallinity and growth rate of COFs.^[^ ^152^ ^]^ Even though no spectroscopic techniques were employed to capture intermediate species, yet for completeness, we want to mention that time‐dependent PXRD studies could show that 2D imine‐linked COFs initially form as crystalline sheets. In the next step, they undergo reorganization to form more ordered stacked structures. This stacking process, which occurs over several hours, improves the stability of the COFs and reveals a revised mechanistic understanding of their formation.^[^ ^153^ ^]^

Recent studies using interferometric scattering microscopy (iSCAT) have provided further insights into COF polymerization and framework formation. These studies observed liquid–liquid phase separation, suggesting the presence of structured solvents in the form of surfactant‐free (micro)emulsions during conventional COF synthesis. This finding reveals that solvents play a role beyond merely dissolving reactants.^[^ ^94^ ^]^

The role of additives was also studied during the mechanochemical synthesis of COFs using time‐resolved synchrotron PXRD and Raman spectroscopy. Emmerling et al. revealed the significant impact of liquid additives and catalysts on the reaction kinetics and crystallinity of the final product. Specifically, these studies highlighted the presence of crystalline intermediates, providing the first direct structural evidence of templating effects by small solvent molecules. The initial step of imine condensation, identified by the appearance of a strong Raman band at 1623 cm^−1^, underscores the critical role of these additives in guiding COF assembly.^[^ ^154^ ^]^

The synthesis of reticular materials is inherently complex, involving multiple reaction steps and intermediates that are difficult to capture with ex situ methods alone. In situ studies have shed light on crucial aspects of nucleation and crystallization, revealing the roles of pre‐nucleation clusters and amorphous intermediates that lead to the final crystalline structures. Recent technical advancements of methods like XAS, NMR, and vibrational spectroscopy now allow us for the first time to track metal‐ligand coordination, intermediate species, and structural transitions in real‐time. Despite these advancements, challenges remain in fully understanding the molecular mechanisms of framework assembly, particularly in the short‐lived early stages of synthesis.

#### 3.1.2 Functionalization and Post‐Synthetic Treatments

Activation, functionalization, and post‐synthetic modifications are essential strategies for enhancing the properties of reticular materials by altering metal nodes, modifying linkers, or introducing new functional groups. Here, in situ approaches have transformed our understanding of these newly generated materials. For example, the coordination environment of metal nodes during dehydration or the real‐time monitoring of nanoparticle formation within MOFs has provided insights critical for tuning their properties. Key techniques used in studying functionalization and post‐synthetic treatments include XAS, NMR, and vibrational spectroscopy. XAS and NMR spectroscopy provide insights into the structural and electronic changes during activation, while Raman and FTIR spectroscopy track the introduction of functional groups or metal ions and confirm successful functionalization. Certainly, other techniques also come into play for characterizing the modified materials, for instance, fluorescence‐based methods in case the introduced modifications are luminescent or lead to changes in fluorescent material properties. Equally, UV‐Vis and electrochemical analysis are employed to assess changes in optical and redox properties post‐functionalization. The following section discusses the role of in situ techniques in studying heat treatments and activation processes, where real‐time monitoring pushes the boundaries of material design and synthesis.

##### Heat Treatments and Activation

Heat treatments and activation processes have been extensively studied using a combination of in situ XAS, DRIFT spectroscopy, and Raman spectroscopy, to gain insights into the structural and electronic changes that occur during dehydration, oxidation, and activation of MOFs. In situ DRIFT spectroscopy and thermogravimetric‐differential scanning calorimetry (TG–DSC) were used to study the dehydration and rehydration processes of UiO‐66 and UiO‐67. The findings revealed that while UiO‐67 displayed the expected symmetric ν(OH) band, multiple bands emerged for UiO‐66 before dehydration, indicating a more complex dehydration process. Both materials showed that dehydration was fully reversible upon rehydration.^[^ ^155^ ^]^ In situ Fe K‐edge XAS was employed to study the oxidation state and coordination of Fe in the Fe₃‐µ₃‐oxo nodes of PCN‐250 after thermal activation and reaction with N_2_O and CH_4_. The study revealed changes in the Fe coordination and oxidation state, forming Fe sites that resemble those in α‐ketoglutarate‐dependent dioxygenases.^[^ ^156^ ^]^

The oxidation state and composition of Cu clusters deposited in NU‐1000 were monitored using in situ XAS during thermal treatment. The study showed that Cu atoms existed as a combination of CuO and Cu(OH)_2_ at room temperature, with OH and O groups forming bridges with Zr_6_ nodes in the MOF. Under reducing environment, these bonds broke. Consecutively, Cu atoms could aggregate into larger nanoparticles, demonstrating the dynamic nature of metal clusters within MOFs.^[^ ^157^ ^]^ In situ XAS at the Cu K‐edge was used to study the dehydration of HKUST‐1, revealing that the removal of coordinated water molecules from Cu(II) sites did not alter the oxidation state of copper. Yet, the crystalline structure of the material was preserved, and the cell volume decreased due to the shrinking of the [Cu_2_C_4_O_8_] cage.^[^ ^158^ ^]^ XAS and Raman spectroscopy were used in combination to study local structural changes in a flexible MOF composed of Co(II) ions connected through oxygen‐bridged bis(benzoic acid) linkers. The study revealed that activation of the MOF, through the removal of coordinated water molecules, led to significant changes in the local coordination geometry of the Co sites. XANES provided insights into these changes, while EXAFS analysis confirmed the expected reduction in coordination number.^[^ ^159^ ^]^ Furthermore, studies on coordination exchange processes, like those observed in MOF‐74(Ni) using in situ ¹H NMR and Raman spectroscopy, have demonstrated novel methods for activating thermally unstable MOFs without causing structural damage. These findings provide new strategies for both the functionalization and stabilization of these frameworks.^[^ ^160^ ^]^

##### Ligand Exchange and Ligand Functionalization

Techniques such as in situ NMR spectroscopy have been essential in monitoring solvent‐assisted ligand exchange (SALE) and post‐synthetic modifications in real time. For example, in the functionalization of UiO‐67(Zr) and DUT‐5(Al), in situ NMR spectroscopy allowed the observation of both the entering and leaving ligands during SALE, providing valuable insights into the reaction dynamics without the need to digest the product for analysis.^[^ ^89^ ^]^ Other studies have used complementary spectroscopic techniques, such as FTIR, UV–vis, and XANES, to follow the formation of (arene)Cr(CO)_3_ species within MOFs like UiO‐66(Cr). These techniques, combined with DFT calculations, allowed for detailed tracking of the vibrational and electronic changes during the formation of these complexes. This provided structural evidence of functionalization and offered a pathway for systematic investigations of functionalized porous matrices.^[^ ^161^ ^]^ Moreover, innovative techniques like in situ transmission IR spectroscopy in alkane solvents have enabled the study of UiO‐66 MOF‐immobilized transition‐metal carbonyl complexes in solution. This approach not only mimics the behavior of homogeneous systems but offers new possibilities for mechanistic studies of single‐molecule heterogeneous catalysts. It represents a significant advancement in the functionalization of MOFs for catalytic applications.^[^ ^162^ ^]^ Fluorescence intensity and lifetime imaging microscopy during post^−^synthetic exchange in UiO‐67 have revealed that bulky linkers penetrate narrow‐window crystals by a process called through‐backbone diffusion.^[^ ^163^ ^]^ This process creates a gradient of missing‐linker defects from the surface to the interior of the crystal. This finding expands the range of MOFs that can accommodate large guest molecules, a feature previously thought to be possible only in larger‐pore structures.

##### Formation of Nanoparticles in MOFs

Methods like FTIR, UV–vis, and XANES have been used to track the formation of metal complexes within MOFs. In situ EXAFS and FTIR spectroscopy have also been employed to study the formation and growth of nanoparticles within MOFs, revealing how post‐synthetic modifications can control nanoparticle size and distribution. In situ EXAFS has been utilized to characterize the formation of palladium nanoparticles (NPs) within UiO‐67 MOFs by monitoring the transition of Pd ions from linkers into small NPs and their subsequent agglomeration. This work highlighted how post‐synthetic modifications, such as the substitution of bdc linkers with amino‐functionalized linkers, can control NP size and distribution, with smaller NPs confined within the MOF pores due to stronger interactions with NH_2_ groups.^[^ ^164^ ^]^ In a related study, the functionalization of UiO‐66 with Pd nanoparticles was achieved by substituting bdc linkers with amino‐functionalized benzoic acid.^[^ ^165^ ^]^ In this process, Pd NPs formed within the defective pores of the MOF were significantly smaller (<3.3 nm) compared to those formed on the surface of the nonmodified MOF. The growth of these NPs was monitored in situ using FTIR spectroscopy, which revealed interactions between Pd‐containing anions and NH_2_ groups. The study found that the rate of Pd NPs nucleation and growth was three times slower for the amino‐modified MOF compared to the nonmodified version. Øien et al. studied UiO‐67 functionalized with platinum bipyridine coordination complexes using in situ XANES and EXAFS. The research monitored the structural and oxidation state changes of Pt under different conditions, including reduction, oxidation, and ligand exchange. This provided real‐time insights into the nature of the resulting platinum complexes and their stability within the MOF framework.^[^ ^166^ ^]^

##### Pyrolysis

Supported metal‐on‐carbon materials (metal = Fe, Ni, Cu, etc.) can be obtained from the pyrolysis of MOFs, which extends the field of applications of MOFs by converting them into new materials.^[^ ^167^, ^168^, ^169^, ^170^, ^171^, ^172^, ^173^ ^]^ Although pyrolysis alters the reticular structure of MOFs to the extent that no porous materials remain, the parent framework still significantly influences the properties of the resulting materials, demonstrating the relevance of reticular chemistry in these transformations. In situ Mössbauer spectroscopy, combined with XAS, was used to study the carbonization process of Fe‐based MOF precursors, showing the transformation of Fe species during pyrolysis. Initially, the MOF precursor exhibited 100% high‐spin Fe^3+^ in an octahedral environment. After carbonization, the Fe species were converted into a mixture of iron carbide (FeC_x_) and Wüstite (FeO). Further reduction of these catalysts with hydrogen increased the formation of metallic iron (Fe⁰), which, under Fischer‐Tropsch synthesis (FTS) conditions, was observed to transform into a carbidic phase, indicating dynamic changes in Fe coordination and oxidation states during the catalytic process.^[^ ^174^ ^]^ Another study examined the structural and electronic changes of Fe during the pyrolysis of the Fe‐btc MOF to create FTS catalysts. The decomposition of Fe‐btc, primarily through decarboxylation, resulted in a carbon matrix around Fe nanoparticles. Pyrolysis at different temperatures (400, 600, and 900 °C) produced various Fe carbide phases, with smaller epsilon carbides forming at lower temperatures and larger carbides at higher temperatures.^[^ ^175^ ^]^

In situ techniques have provided invaluable real‐time insights into the roles of pre‐nucleation clusters, intermediates, and amorphous phases, which are critical for optimizing material synthesis. Despite these advancements, there is a need for further development of faster and more spatially resolved measurements. Such approaches will enable the tracking of both temporal and spatial dynamics during the formation process, offering a more complete picture of how reticular frameworks assemble and evolve over time and space.

### 3.2 Structural Dynamics and Stability

In this section, we explore techniques that directly measure the motion of structural components within the framework of reticular materials. These methods allow us to observe how external triggers, such as temperature, light, electric and magnetic fields, and pressure, influence the material's structure and behavior, providing insights into the mechanisms behind flexibility and phase transitions. The flexibility of MOFs is a prominent topic in the field and has been extensively reviewed, particularly from the perspective of the framework itself.^[^ ^12^, ^13^, ^176^, ^177^, ^178^ ^]^ Within this field, gas sorption coupled with in situ NMR spectroscopy, EXAFS and EPR spectroscopy have been proven useful to probe changes of the geometry of the metal node or pore environment. While our focus is on the framework's response to these stimuli, the chemical and structural changes experienced by the guest molecules due to confinement are outside the scope of this review. For further details on guest molecule behavior, we refer readers to the relevant literature.^[^ ^5^, ^24^, ^179^, ^180^ ^]^

**Figure 6:**
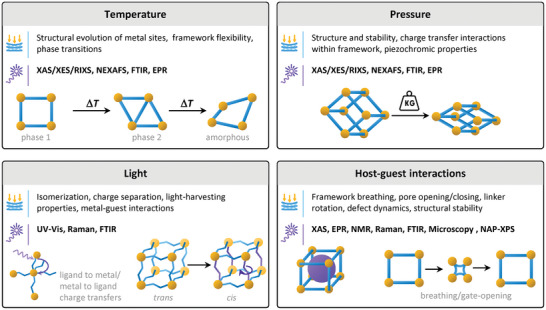
Spectroscopic techniques used to investigate dynamic processes in reticular materials under various external stimuli.

Organized by the type of external trigger, we discuss key in situ techniques that capture these structural responses in time (**Figure** **6**).

#### 3.2.1 Pressure‐Induced Changes

In situ and operando techniques are indispensable for monitoring structural changes in reticular materials under varying pressure conditions. These methods enable real‐time tracking of how frameworks respond to external forces, providing critical insights into their stability, flexibility, and phase transitions. In situ vibrational spectroscopy and spectroelectrochemistry are particularly suited to study pressure‐induced transformations, revealing both reversible and irreversible structural changes, and shedding light on the role of guest molecules in stabilizing frameworks under compression. In situ Raman and FTIR spectroscopy were used to study the effects of high external pressure on the structure and stability of the MOF α‐Mg_3_(HCOO)_6_.^[^ ^181^ ^]^ The framework remained chemically stable under compression, but an irreversible crystal‐to‐crystal structural transition occurred above 2 GPa. Interestingly, when loaded with guests like DMF or benzene, no such transition was observed. In another study, the pressure‐induced shifts in the fingerprint region of ZIF‐8 were examined using IR spectroscopy.^[^ ^182^ ^]^ While the structural changes up to 39 GPa were found to be reversible, higher pressures led to irreversible transitions into an amorphous phase. In situ solid‐state Raman spectroelectrochemistry was employed to investigate a redox‐active MOF [(Zn(DMF))_2_(ttftc)(dpni)] (ttftc = tetrathiafulvalene tetracarboxylate and dpni =  *N*,*N*′‐di(4‐pyridyl)‐1,4,5,8‐naphthalenetetracarboxydiimide), revealing changes in the vibrational features of redox‐active ligands under applied potential, indicative of local structural modifications.^[^ ^183^ ^]^ Additionally, variable‐pressure Raman spectroscopy, using a diamond anvil cell, provided insights into how pressure influences charge transfer interactions within the framework. Band structure calculations further elucidated the mechanism of charge transport in this donor–acceptor MOF.

Recent studies showed that 3D COFs also exhibit unique pressure‐induced emission behaviors with reversible properties.^[^ ^184^ ^]^ For instance, bicarbazole‐based COFs demonstrated a remarkable pressure‐induced emission enhancement under hydrostatic pressure, with a 16‐fold increase in fluorescence intensity and blue‐shifted emission as 3D COF. High‐pressure in situ FTIR spectroscopy revealed small shifts of the deformation vibrations of C‐H bonds at 5 GPa attributed to complex intramolecular interaction within the frameworks. Flexible frameworks with dia topology turned out to be readily activatable by pressure compared to more rigid qtz ones. In contrast, the introduction of more rigidity and conjugation in 2D COFs, like JUC‐655, led to red‐shifted emission and fluorescence quenching due to π‐π stacking interactions between pyrene and olefin structures, as probed by photoluminescence and FTIR spectroscopy. This study underscores the potential of in situ techniques in exploring the piezochromic properties of COFs and advancing their application in photonic and pressure‐sensing devices.

#### 3.2.2 Light‐Induced Changes

The ability of reticular materials to undergo light‐induced transformations has opened new avenues for applications in fields like sensors, data storage, and energy conversion.^[^ ^44^, ^185^ ^]^ Studies have demonstrated how structural changes, like *trans*‐to‐*cis* isomerization or charge separation, can be induced and reversed by light exposure, altering the material's properties. Techniques such as UV‐Vis, Raman, and FTIR spectroscopy have been instrumental in tracking these dynamic transformations in real‐time. For instance, the photoresponsive behavior of a two‐fold interpenetrated MOF ([Zn_2_(ndc)_2_(azo‐bpy)] using 3‐azo‐phenyl‐4,4′‐bipyridine (azo‐bpy), 2,6‐naphthalenedicarboxylic acid (ndc), and Zn(NO_3_)_2_·6H_2_O; CAU‐5) was demonstrated through the reversible *trans*‐to‐*cis* isomerization of azo‐functionalized linkers upon UV light exposure.^[^ ^44^ ^]^ The structural changes were tracked using UV–vis spectroscopy. The process is reversible with thermal treatment or visible light, showcasing the potential for light‐driven control of MOF properties. A new Hofmann‐type Fe(II) SCO‐MOF ([Fe(bpn){Ag(CN)_2_}_2_]⋅azobenzene (bpn = 1,4‐bis(4‐pyridyl)naphthalene)) incorporating azobenzene as a photoactive guest exhibited a reversible *trans*–*cis* photoisomerization under UV–vis light, which led to significant changes in the material's magnetic properties. DUT‐163, a MOF featuring tetra‐connective carbazole‐based ligands with an azo‐bridge linked to copper(II) dimers, undergoes structural contraction triggered by light irradiation and guest molecule adsorption stress. Unlike the trans‐to‐cis transformations via E‐Z isomerization observed in other MOFs, DUT‐163 exhibits a unique buckling mechanism of the linker, as revealed by in situ UV–vis and Raman spectroscopy.^[^ ^186^ ^]^ Another study demonstrated that copper‐based MOFs incorporating photoresponsive diarylethene‐ and spiropyran‐containing linkers undergo reversible light‐induced switching between Cu(II) and Cu(I) oxidation states. This phenomenon facilitates enabled cyclic optical and electronic responses, which were monitored using UV–vis and EPR spectroscopy.^[^ ^187^ ^]^

A study utilizing UV–vis–NIR spectroscopy explored the enhanced light harvesting and photoelectric conversion of a pyrene MOF upon encapsulation of a D–π–A cyanine dye.^[^ ^188^ ^]^ The MOF's interaction with the dye extended its light‐harvesting range from visible to NIR, indicating strong metal‐guest interactions and the tunability of the MOF's photophysical properties. A combined Vis‐NIR and Raman spectroscopic study demonstrated strongly anisotropic absorption behavior in MOF‐74(Co) crystals when illuminated with polarized light.^[^ ^189^ ^]^ The study detected different peak shifts in the Vis‐NIR spectra, which were correlated with metal‐guest interactions. Guests like propene, CO_2_, methanol, and water exhibited stronger interactions with Co(II) centers than other guests like propane and argon. MOF‐5 was found to behave as a semiconductor upon light excitation, undergoing charge separation that results in electrons and holes with microsecond lifetimes.^[^ ^185^, ^190^ ^]^ This phenomenon was observed using time‐resolved diffuse reflectance UV–vis spectroscopy. A study utilizing UV–vis–NIR spectroscopy explored the enhanced light harvesting and photoelectric conversion of a pyrene MOF upon encapsulation of a D–π–A cyanine dye.^[^ ^188^ ^]^ The MOF's interaction with the dye extended its light‐harvesting range from visible to NIR, indicating strong metal‐guest interactions and the tunability of the MOF's photophysical properties. A combined Vis‐NIR and Raman spectroscopic study demonstrated strongly anisotropic absorption behavior in MOF‐74(Co) crystals when illuminated with polarized light.^[^ ^189^ ^]^ The study detected different peak shifts in the Vis‐NIR spectra, which were correlated with metal‐guest interactions. Guests like propene, CO_2_, methanol, and water exhibited stronger interactions with Co(II) centers than other guests like propane and argon. MOF‐5 was found to behave as a semiconductor upon light excitation, undergoing charge separation that results in electrons and holes with microsecond lifetimes.^[^ ^185^, ^190^ ^]^ This phenomenon was observed using time‐resolved diffuse reflectance UV–vis spectroscopy.

Recently, COFs incorporating light‐driven molecular motors and photoresponsive anthracene units were synthesized, with their structural dynamics and transformations monitored using in situ FTIR, Raman, and UV–vis spectroscopy. These studies revealed that the COFs can undergo reversible structural changes, such as [4π+4π] cycloaddition, when exposed to light.^[^ ^191^ ^]^ Due to these capabilities, COFs are emerging as an ideal class of smart materials for photomechanical applications such as information storage, sensors, artificial muscles, and self‐healing materials.

#### 3.2.3 Temperature‐Induced Changes

Several spectroscopic studies have demonstrated that heating MOFs can induce significant structural and electronic modifications. For instance, the thermal activation of MIL‐100(Fe) revealed changes in the iron sites, with detailed insights provided by High‐Energy Resolution Fluorescence Detected XAS (HERFD‐XAS), EXAFS, XES, and Resonant Inelastic X‐ray Scattering (RIXS), showing how the framework evolves under temperature stress.^[^ ^192^ ^]^ A study using Cu L_3_‐edge XAS and ambient‐pressure NEXAFS spectroscopy examined HKUST‐1 from room temperature to 160 °C under various gas environments.^[^ ^193^ ^]^ The results showed that mild annealing at 160 °C led to dehydration of Cu(II) sites, forming square‐planar‐coordinated clusters and partially reduced Cu(I)/Cu(II) dimers, particularly on the material's surface. These surface defects were more abundant compared to the bulk defects. Additionally, decarboxylation of some paddlewheel units contributed to the formation of these dimers. Furthermore, this study also revealed that CO_2_ interacts with HKUST‐1 through a redox‐active transition at these Cu(I) defective sites. Certain MOFs experience temperature‐induced structural changes, such as the reversible breathing transition observed in MIL‐53(Al). This transition, which involves a shift between narrow‐pore and large‐pore phases, was monitored using in situ EPR spectroscopy with V(V)═O molecular ions as probes.^[^ ^194^ ^]^ In another example, ZIF‐4 was observed to undergo thermally induced amorphization, followed by recrystallization into the densest ZIF‐zni structure upon further heating.^[^ ^195^ ^]^ This was tracked using in situ synchrotron far‐infrared spectroscopy, which highlighted the connection between thermal stimulus, framework flexibility, and the likelihood of amorphization. Certain MOFs experience temperature‐induced structural changes, such as the reversible breathing transition observed in MIL‐53(Al). This transition, which involves a shift between narrow‐pore and large‐pore phases, was monitored using in situ EPR spectroscopy with V(V)═O molecular ions as probes.^[^ ^194^ ^]^ In another example, ZIF‐4 was observed to undergo thermally induced amorphization, followed by recrystallization into the densest ZIF‐zni structure upon further heating.^[^ ^195^ ^]^ This was tracked using in situ synchrotron far‐infrared spectroscopy, which highlighted the connection between thermal stimulus, framework flexibility, and the likelihood of amorphization.

In a recent study, in situ cryogenic FTIR spectroscopy was used to observe temperature‐dependent structural dynamics in a COF series, including COF‐300, COF‐300‐amine, and COF‐V.^[^ ^196^ ^]^ This technique revealed reversible peak shifts in the IR spectrum as the temperature dropped from 298 K to 30 K and back, indicating conformational changes in the frameworks. Several modes, in particular the C‐C‐H and imine stretch vibrations experienced significant blue shifts. These shifts were linked to pedal motion within the framework, where molecular components rotate or shift under cooling. This study represents an excellent example showcasing how in situ techniques can track molecular rearrangements with precision and, hence, probe mechanical properties of reticular materials.

#### 3.2.4 Guest–Molecule‐Induced Changes

Guest molecules can induce significant structural changes in reticular materials, including framework breathing, pore opening/closing, and linker rotations under varying environmental conditions such as pressure, temperature, and gas composition. In situ techniques like EXAFS, EPR, NMR, or Raman spectroscopy can probe how these guest‐induced transformations occur at the atomic and molecular levels. Recent studies have also demonstrated the effectiveness of advanced imaging techniques, such as infrared nanospectroscopy and correlative Raman microscopy, in visualizing guest interactions and defect dynamics within MOFs at the single‐crystal level.

##### Flexibility and Breathing of the Framework

The flexibility of MOFs upon guest adsorption has been extensively explored using techniques like in situ EXAFS and EPR spectroscopy. For instance, in situ EXAFS (Ni K‐edge) was used during nitrogen adsorption to track changes in the local coordination geometry of metal atoms during structural transformations in flexible MOFs like DUT‐8(Ni), revealing significant local deformations in the nickel‐based paddle‐wheel nodes.^[^ ^197^ ^]^ The findings illustrate the reversible nature of these structural changes. Additionally, the framework exhibited varying degrees of structural transformations when exposed to different probe molecules, such as nitrogen, carbon dioxide, and n‐butane, each interacting uniquely with the MOF.^[^ ^197^ ^]^ MAS‐SS NMR was applied to DUT‐8(Ni), revealing guest‐induced changes in the local coordination environment of the nickel paddle‐wheel nodes during nitrogen adsorption.^[^ ^59^ ^]^ The study identified unique local deformations of the nickel nodes, emphasizing the importance of combining spectroscopic and structural techniques to fully understand the dynamics of flexible MOFs. In situ X‐ray spectroscopy techniques, including EXAFS and HERFD‐XANES, were employed to study the gate‐opening behavior in the ZIF‐8 framework during nitrogen gas adsorption.^[^ ^198^ ^]^ EXAFS analysis revealed that small shifts in the Zn‐C_1_ distance and Zn‐Zn_1_ distances occurred, corresponding to ligand movement and lattice expansion, while the ZnN_4_ tetrahedron remains rigid. In situ HERFD‐XANES provided detailed spectral insights, showing that the structural changes involved the rotation of the methylimidazole ligand and bending of the methyl group around the Zn atom.

In situ MAS‐SS‐NMR combined with XRD was used to study the breathing behavior of MIL‐53 (terephthalate linker) and MIL‐53‐ADP (adipate linker) upon water adsorption and desorption.^[^ ^199^ ^]^ The original MIL‐53 exhibited a single‐step breathing process, while MIL‐53‐ADP displayed a two‐step behavior, reflecting the impact of linker flexibility on the structural dynamics of the MOF. An emerging technique, Dynamic Nuclear Polarization (DNP)‐assisted SS‐NMR spectroscopy, has demonstrated significant potential for enhancing sensitivity in the study of reticular materials. By amplifying weak NMR signals through polarization transfer, this method holds promise to enhance insights into guest molecule dynamics within frameworks.^[^ ^200^ ^]^

For DUT‐49, in situ^[^ ^124^ ^]^ Xe NMR spectroscopy was used to probe adsorption‐induced structural transitions, showing that higher defect concentrations can impede structural contractions typically observed in defect‐free MOFs.^[^ ^201^, ^202^ ^]^ It was found that smaller crystal sizes reduce solid‐fluid interactions, minimizing the driving force for structural transitions, which highlights the intricate role of defects and crystal size in the adsorption behavior and flexibility of MOFs.^[^ ^201^ ^]^

EPR spectroscopy has also proven valuable in studying the breathing behavior of MOFs. For example, in DUT‐49(Cu), EPR spectroscopy was used to monitor the phase transitions and adsorption/desorption kinetics, showing changes in the zero‐field splitting of intrinsic paddle‐wheel units during these processes.^[^ ^55^ ^]^ This approach is advantageous due to its high sensitivity and ability to perform in situ measurements across a range of temperatures. In another study, EPR spectroscopy was used to investigate flexible mixed‐metal MOFs (Zn_1.9_Cu_0.1_(bme‐bdc)_2_(dabco) (bme‐bdc^2–^  = 2,5‐bis(2‐methoxyethoxy)‐1,4‐benzene‐dicarboxylate)) with Cu(II) dopants during CO_2_ adsorption and desorption. The study found that Cu(II) dopants did not alter the phase transition behavior significantly but provided detailed information on the local structural changes during the transition.^[^ ^203^ ^]^

Breathing is not restricted to MOFs alone. In situ XRD combined with gas adsorption studies and in situ IR spectroscopy, allowed for a comprehensive analysis, for instance, of COF‐300′s breathing behavior.^[^ ^204^ ^]^ XRD tracked structural changes during guest adsorption, while IR spectroscopy probed molecular interactions and bond shifts, such as those in the imine groups. Gas adsorption isotherms revealed a stepwise behavior correlating with pore transitions (opening/closing) captured in real‐time by in situ XRD. This combination of techniques elucidated the flexible pore structure and selective adsorption properties of COF‐300 for benzene over cyclohexane.

##### Reticular Materials and CO_2_

CO_2_ interaction with MOFs has been a key area of study, particularly in understanding how it affects structural stability and adsorption capacity. In ZIF‐8, in situ FTIR spectroscopy demonstrated that CO_2_ could be inserted into the framework at high pressures around 0.8 GPa, differentiating CO_2_ molecules inside the framework from those in the bulk medium. The interaction was shown to significantly enhance CO_2_ storage capacity, with IR features of the framework revealing interaction sites.^[^ ^205^ ^]^ Similarly, in Mg_2_(dobdc) (H_4_dobdc = 2,5‐dihydroxyterephthalic acid; MOF‐74(Mg), CPO‐27(Mg), a prominent MOF known for exceptional CO_2_ capture properties, in situ ^13^C NMR spectroscopy provided detailed insights into the binding and rotational motion of CO_2_ within the material. This study revealed that CO_2_ interacts strongly with the open metal sites.^[^ ^206^ ^]^

COFs have recently gained attention for their applications in CO_2_ capture and electrochemical reduction, since their structures can be precisely tuned to enable efficient CO_2_ adsorption and activation. In CO_2_ electroreduction, COFs can modulate the catalytic microenvironment, improving selectivity for products like methane by optimizing intermediate adsorption and proton availability. A recent study demonstrates the potential of COFs in steering the CO_2_ electroreduction pathway using Cu electrodes.^[^ ^207^ ^]^ In this work, in situ attenuated total reflection surface‐enhanced infrared absorption spectroscopy (ATR‐SEIRAS) combined with X‐ray photoelectron spectroscopy (XPS) were key techniques. In situ ATR‐SEIRAS allowed real‐time tracking of CO adsorption on Cu surfaces during CO_2_ electroreduction, revealing lower *CO coverage in COF‐modified systems. XPS depth profiling further confirmed the enrichment of potassium ions within the COF framework, illustrating how the COF modulates the local microenvironment.

##### Reticular Materials and Other Guests

NAP‐XPS was employed to explore the interaction of HKUST‐1 with vapors of water, pyridine, and methanol. The study revealed that exposure to water vapor and pyridine led to the reduction of copper sites, while methanol did not cause such changes, illustrating the technique's ability to probe surface chemistry and the influence of different gases on MOF stability.^[^ ^208^ ^]^ The uptake of D_2_O into ZIF‐8 and HKUST‐1 was studied using IR nanospectroscopy, which provided site‐specific insights into D_2_O‐induced defects. This approach allowed real‐time visualization of sorption mechanisms and defect dynamics, highlighting the interaction between D_2_O and the framework's hydrophilic and hydrophobic domains.^[^ ^108^ ^]^ Similarly, correlative Raman spectroscopy in combination with coherent anti‐Stokes Raman scattering microscopy and Four‐Wave Mixing microscopy could follow water uptake in situ at the single crystal level^.[^ ^209^ ^]^ In addition, correlative Raman/fluorescence/nonlinear imaging combined with Raman spectroscopy probed the influence of particle heterogeneities influence the intrinsic and extrinsic guest uptake properties of UiO‐67 and MIL‐88A.^[^ ^210^ ^]^

COFs exhibit guest‐induced structural changes beyond CO_2_, which have been studied using techniques like fluorescence spectroscopy and dark‐field microscopy. In dynaCOF‐330, the interaction with organic vapors led to structural transformations that were accompanied by changes in fluorescence intensity and emission wavelength, depending on the type and concentration of the vapor.^[^ ^211^ ^]^ The study utilized in situ fluorescence spectroscopy and other complementary techniques to correlate these changes with the flexibility of the framework and the adaptive inclusion of guest molecules. In another study, the flexible deformation behaviors of COF‐300 and its reduced form, COF‐300‐AR, were tracked using dark‐field microscopy. The findings showed that despite having more flexible C─N single bonds, COF‐300‐AR exhibited less flexibility than COF‐300, suggesting that the rigid C═N double bonds play a crucial role in the framework's structural adaptability.^[^ ^212^ ^]^

In summary, in situ and operando techniques have provided valuable insights into how external factors like pressure, temperature, light, and guest molecules affect the structure and stability of reticular materials. However, it remains challenging to fully understand these complex changes at both large and small scales. While current techniques can track important processes like defect formation or structural changes, they still struggle to capture small, localized events across entire materials. Future research on guest‐induced changes in reticular materials could benefit from in situ imaging techniques like Scanning Transmission Electron Microscopy (STEM), which has been effective in visualizing sorption‐induced topological changes in zeolites.^[^ ^213^ ^]^ Applying in situ STEM to MOFs and COFs would allow for atomic‐level observation of interactions with organic guest molecules, revealing subtle structural rearrangements during sorption.

### 3.3 Defects in Reticular Materials

**Figure 7:**
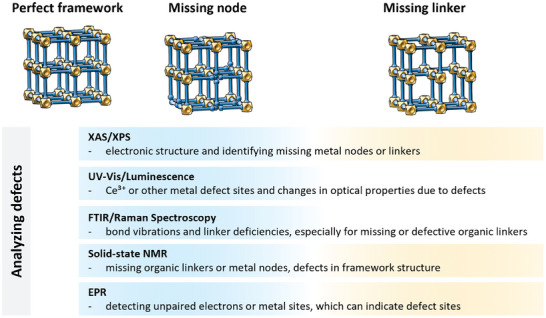
Types of defects and spectroscopic methods to analyze them in reticular materials.

Defects in MOFs and COFs play a critical role in influencing a wide range of material properties, from stability and adsorption capacity to catalytic performance and thermal behavior.^[^ ^214^, ^215^, ^216^ ^]^ These structural imperfections, which deviate from the idealized crystal lattice, can significantly alter the functionality of the frameworks. While defects are integral for tuning these materials, their identification and detailed analysis are still emerging areas of research.^[^ ^10^ ^]^ The application of in situ and operando characterization techniques can provide insights into how defects develop and change under various conditions, enabling more effective manipulation and optimization of framework properties. As defects are often missing linkers and thus open coordination sites on the metal node (**Figure** **7**), which both can be identified with vibrational spectroscopy—in situ IR spectroscopy is predominantly used in this field, while Raman imaging has provided spatial resolution. Recent advancements, such as the use of Fluorescence‐Lifetime Imaging Microscopy (FLIM), have further enhanced our ability to resolve and analyze nanoscale defects within MOFs.

Using FTIR spectroscopy, the crystallization process of Zr‐fum MOF (fum = fumarate) in both water‐ and DMF‐based modulated synthesis systems was studied.^[^ ^217^ ^]^ The study revealed that in the DMF‐based system, the acid modulator HCOOH was consumed during the crystallization process. In contrast, the water‐based system did not show such consumption. The findings suggest that the consumption of HCOOH in the DMF‐based synthesis leads to the formation of structural defects in the resulting Zr‐fum MOF. Solid‐state NMR further supported the participation of HCOOH in the structure formation, highlighting the impact of the synthesis environment on defect formation. The presence of Ce(III) as a defective site in UiO‐66(Ce) was qualitatively verified using UV‐Vis and FTIR spectroscopy. This study demonstrated that these spectroscopic techniques could effectively identify and characterize defect sites within MOFs, providing a valuable tool for assessing the impact of defects on the material's properties.^[^ ^218^ ^]^ In situ temperature‐programmed IR spectroscopy and MS, combined with computational studies, were employed to identify NH_3_ binding sites in UiO‐67 MOFs, including those associated with missing linker defects. This study monitored the evolution of these binding sites as a function of the activation temperature of the MOF.^[^ ^219^ ^]^ The research provided insights into the relationship between activation temperature and the availability of defect sites within the framework, emphasizing the role of these defects in the material's performance. A study on MIL‐100(Fe) investigated the presence and amount of Fe(II) and Fe(III) coordinatively unsaturated sites (CUS) and their role in separating a propane/propene mixture^.[^ ^220^ ^]^ Using an operando IR methodology, the research highlighted the influence of these defect sites on the gas separation efficiency of MIL‐100(Fe), showing that the affinity of unsaturated Fe(II) sites to C═C bonds drives the separation process

Further research on defects in MOFs has employed advanced, spatially resolved techniques to gain deeper insights into defect characteristics. For example, Raman imaging allows for spatial mapping of defects in HKUST‐1‐like MOFs to monitor their three‐dimensional distribution.^[^ ^221^ ^]^ Similarly, two‐photon fluorescence imaging and Raman spectroscopy combined with nonlinear microscopy identified and characterized structural defects in MOF‐801(Zr).^[^ ^222^ ^]^ The defects in mesoporous Cu(II)‐MOF MFM‐100 were characterized using fluorescence microscopy together with Inelastic Neutron Scattering (INS), EPR, and FTIR spectroscopy, revealing uncoupled Cu(II) centers as catalytically active sites during aerobic oxidation of alcohols.^[^ ^223^ ^]^ With fluorescence imaging, defects can be visualized in single crystals of carboxylate‐based MOFs (HKUST‐1 and MOF‐5), using the catalytic oligomerization of furfuryl alcohol as a probe to generate a fluorescence signal that maps the reactivity of acidic sites resulting from defect formation.^[^ ^224^ ^]^ Using, FLIM, the chemical diversity and structural features of UiO‐67 could be even analyzed in three dimensions.^[^ ^225^ ^]^ The technique revealed nanoscale defects and heterogeneity within single crystals and across samples. FLIM proved effective in identifying variations in defects and functional groups, offering a powerful, non‐invasive tool for probing the chemical complexity of MOFs.

Defects also play an important role in COFs, where defect sites are quantified using vibrational spectroscopy of the solid material or ^1^H NMR spectroscopy after dissolving or degrading the COF.^[^ ^214^ ^]^ In situ studies on the formation of defects are yet to be explored.

While characterization techniques have advanced significantly, precisely identifying and controlling defects remains a challenge. Spatially resolved methods like Raman^[^ ^79^, ^226^, ^227^ ^]^ and fluorescence^[^ ^224^, ^225^ ^]^ microscopy as well as scanning electron diffraction^[^ ^228^ ^]^ have greatly enhanced our understanding of defects through in situ and operando measurements. While these techniques have achieved significant breakthroughs in imaging porous materials,^[^ ^111^, ^210^, ^213^ ^]^ capturing such detailed insights under operando conditions remains a challenge. This represents a growing area of research, as further advancements are needed to improve real‐time imaging of defects and structural changes in dynamic environments.

### 3.4 Catalytic Processes

In situ and operando spectroscopy has become essential tools for studying reticular materials in catalysis.^[^ ^2^, ^42^, ^46^, ^230^ ^]^ These techniques allow for observing real‐time changes in the structure and chemistry of the catalyst and provide insights into the active sites, reaction intermediates, and mechanisms at play during catalytic reactions. By directly correlating the structural dynamics of the framework with its catalytic activity, in situ and operando methods offer a deeper understanding of how reticular materials can be optimized for specific catalytic applications (**Table** **2**), as we will discuss in detail below.

**Table 2:** Overview catalytic reactions with reticular materials studied in situ or operando.

	Reaction	Materials	Used in situ/operando technique	Conditions	Refs.
Single‐site/single‐atom catalysis	CO oxidation	Pt SACs supported UiO‐67‐Pt	XAS	Hydrogen treatment up to 623 K	[242]
		Cu/UiO‐66	XANES, EPR, DRIFTS		[257]
		Cu‐UiO‐67(Ce)	XAS, DRIFTS	Reaction at atmospheric pressure and 200 °C in CO/O_2_ flow	[243]
	Gas‐phase hydrogenation	Ni‐AIM supported on NU‐1000	XANES	3% H_2_/N_2_ at 200 °C	[244]
	Reductive activation and subsequent PROX reaction	Cu‐SACs supported on UiO‐66	NAP‐XPS, DR‐UV‐Vis, XANES	Ar and H_2_ pretreatment at 250 °C; reaction at 120 °C in gas mixtures (1% CO, 1% O_2_, 80% H_2_, and balance of Ar)	[245]
	NO_2_ reduction	Cu supported on UiO‐66 and NH_2_‐UiO‐66	EPR, MAS NMR, DRIFTS	1 bar NO_2_, room temperature	[256]
Heterogeneous thermocatalysis	Propylene hydrogenation	Bimetallic (Cu_x_Rh_1−x_)_3_(btc)_2_	NAP‐XPS	150 °C pretreatment in UHV; expose to 32 mtorr H_2_/8 mtorr propylene at room temperature	[246]
	Ethylene hydrogenation	Pd nanoparticles within UiO‐67	PXRD, operando XAS, DRIFTS	300 °C H_2_ reduction, 25–80 °C in H_2_/C_2_H_4_ flow during reaction	[247]
	CO_2_ to methanol hydrogenation	Pt‐loaded UiO‐67	FTIR, SSITKA	350 °C activation in 10% H_2_/He, reaction at 170 °C in CO_2_/H_2_ (1/6) flow	[248]
	Methane to methanol conversion	MIL‐100(Fe)	XAS, XES, RIXS, PXRD	He or O_2_ activation, and CH_4_/O_2_ or CH_4_/N_2_ reaction at 200–250 °C	[192]
	CO oxidation	Pt supported on UiO‐67 and ZrO_2_	NAP‐XPS	At 260 °C in CO/O_2_ (total pressure = 3 mbar)	[41]
		PtNi supported on UiO‐67	NAP‐XPS, ex situ HR‐STEM	1 mbar H_2_ reduction (360 °C); 300 °C reaction in CO/O_2_ (3 mbar in total)	[249]
	NO reduction	Cu(I)‐ZrTpmC* MOFs	UV‐Vis, FTIR, EPR	in situ FTIR, UV‐Vis: Ar, NO/Ar flow; in situ EPR: 5% NO, at 4.4 to 55 K	[250]
	Heck C‐C coupling reaction	Pd@MOF (Pd(II)@MIL‐101‐NH_2_(Cr), Pd(0)@MIL‐101‐ NH_2_(Cr), Pd(II)@MIL‐88B‐NH_2_(Cr), and Pd(0)@MIL‐88B‐ NH_2_(Cr))	XAS, PXRD	RT to 90 °C in solvents	[251]
Electrocatalysis	Oxygen Evolution Reaction (OER)	ZIF‐67 doped with Co	UV‐Vis coupled with Raman	1.0 M KOH electrolyte, 0.925 ‐1.705 V	[252]
	OER and Oxygen Reduction Reaction (ORR)	NiFe MOFs	FTIR, XAS	0.1 M KOH, in O_2_/N_2_ flow, 1.0–0.6 V (ORR) and 1.2–1.6 V (OER)	[253]
	CO_2_ Reduction Reaction (CO_2_RR)	Copper(II) phthalocyanine (Cupc), HKUST‐1, and copper(II) 1,4,8,11‐tetraazacyclotetradecane chloride ([Cu(cyclam)]Cl_2_)	XAS	0.5 M KHCO_3_ with CO_2_ flow, ‐1.0 – 0.64 eV	[40]
		HKUST‐1	XAS	1 M KOH, in CO_2_, −2.4 V vs Ag/AgCl	[254]
		Polyoxometalate‐based MOFs	FTIR, XAS	0.5 M KHCO_3_, in CO_2_, −0.5 to ‐1.2 V (versus RHE)	[255]
		Ni‐doped ZIF‐8	NAP‐XPS, ATR‐FTIR		[256]
		MOF‐functionalized GDE	XAS	1 M KOH electrolyte, current density of 300 mA cm^−2^	[257]
		Au nanoneedles embedded in PCN‐222 MOF	XAS, IR	CO_2_‐saturated 0.1 M KHCO_3_, 0 to −1.2 V vs RHE	[258]
		Bipyridyl‐based COF	ATR‐FTIR	0.5 M NaHCO_3_ under Ar at room temperature, 0 to −1.6 V vs SCE	[259]
		Redox‐active COF (tapa‐ope) with Co(II) grafted sites	XAS, FTIR	−0.67 V vs RHE in 0.2 M KHCO_3_	[260]
		Cobalt porphyrin‐based COF	UV‐Vis	pH 7.2 aqueous potassium phosphate buffer (0.2 M) with additives: 0.5 M KHCO_3_ under CO_2_ atmosphere, 0.23 to –0.57 V vs RHE	[261]
	Hydrogen Evolution Reaction (HER)	UU‐100(Co)	UV‐Vis	DMF containing 0.1 M LiClO_4_, ‐0.05 to –1.5 V (vs Fc^+/0^)	[262]
Photocatalysis	Formic acid dehydrogenation	UiO‐66‐(COO)_2_‐Cu	FTIR	*T* = 25 °C; 150 W Xe lamp with a visible‐light‐pass filter (λ > 390 nm); irradiance = 71 mW·cm^−2^;	[254]
	HER	Co/Fe mixed‐node MOF‐74 with [Ru(bpy)_3_]Cl_2_	XAS	450 nm LED, 1.02 mW, in acetonitrile, in presence of TEOA	[264]
		Co‐[Ru(bpy)_3_]was incorporated into the linker of UiO‐67	XAS, optical transient absorption spectroscopy	447 nm LED, 9 mW	[265]
	CO_2_ reduction	[Re(bpy)(CO)_3_]Cl incorporated into 2D triazine COF	UV‐Vis	225 W Xe lamp (420 nm cut off)	[266]
	CO_2_ hydrogenation	UiO‐66‐NH_2_ (Cu)	XAS, EPR	500 W Xe lamp	[267]
	CO_2_ reduction	Porphyrin‐based Zr‐MOF	NAP‐XPS, EPR	In situ EPR: NIR‐light irradiation (λ ≥ 730 nm) at 140 K	[268]
	gas‐phase photooxidation of propylene	Isoreticular MOFs with different linkers	FTIR	100 W Hg lamp	[269]
	reactive oxygen species (ROS) generation	ZIF‐67, ZIF‐8, and UiO‐66	Raman	70 W halide lamp with wavelength: 315–800 nm	[270]
	Photopolymerization	Donor‐acceptor COF	EPR	EPR measurement at 300 K, 300 W Xe lamp, output power 1.5 W	[56]

This chapter is structured into four main sections: single‐site/single‐atom catalysis, thermocatalysis, electrocatalysis, and photocatalysis. Each section will focus on the techniques employed, key findings, and the implications for advancing the design and functionality of these materials in catalysis.

**Figure 8:**
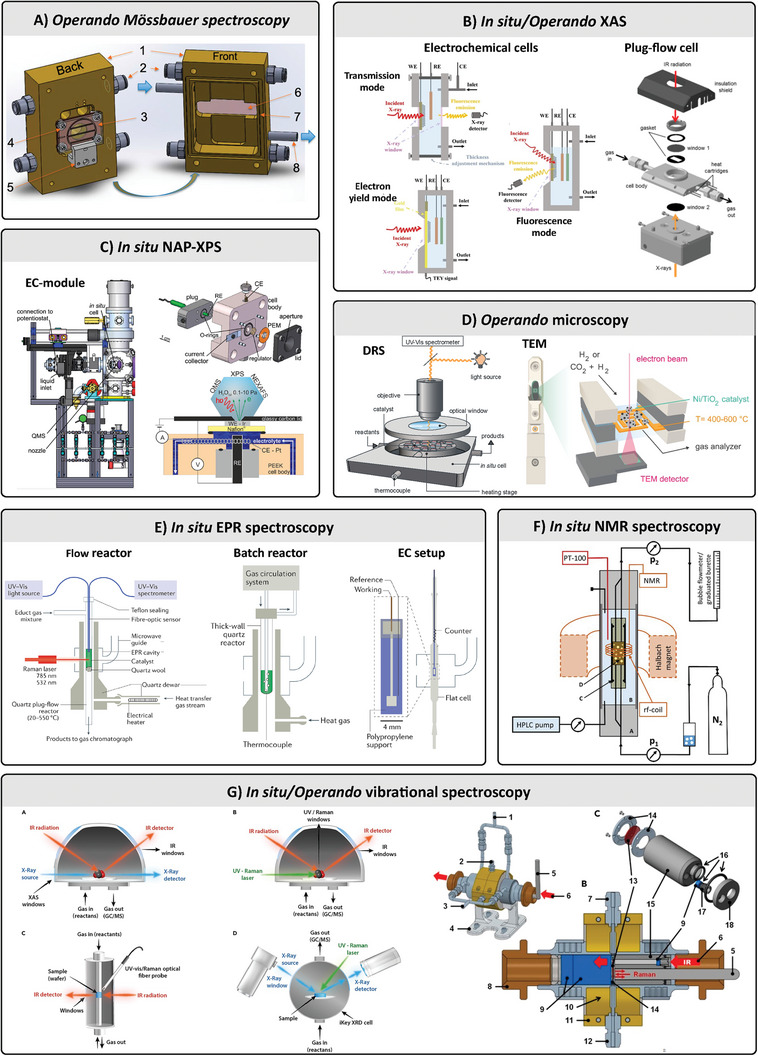
Overview of experimental setups for in situ and operando studies. A) Operando Mössbauer spectroscopy cell, reproduced with permission.^[^ ^234^ ^]^ Copyright 2023, AIP Publishing. B) In situ/operando X‐ray absorption spectroscopy cells for electrocatalytic and thermocatalytic studies: (left) Schematic illustrating the integration of XAS with electrochemical cells for different detection modes, reproduced under the terms of CC BY 4.0 license.^[^ ^235^ ^]^ Copyright 2023, Wiley; (right) plug‐flow cell for combined in situ/operando XAS‐DRIFT measurements, reproduced with permission.^[^ ^236^ ^]^ Copyright 2014, Review of Scientific Instruments. C) NAP‐XPS electrochemistry module and in situ electrochemical cell with continuous liquid flow, reproduced under the terms of the CC BY 4.0 license.^[^ ^237^ ^]^ Copyright 2018, Springer Nature. D) In situ/operando cells for optical imaging: (left) DRS (Diffuse Reflectance Spectroscopy) setup for analyzing solid catalysts during catalytic reaction using reflected light, reproduced with permission.^[^ ^238^ ^]^ Copyright 2023, Springer Nature; (right) schematic of the operando TEM setup and windowed gas cell, reproduced with permission.^[^ ^239^ ^]^ Copyright 2023, American Association for the Advancement of Science. E) In situ EPR probes for catalytic and electrocatalytic reactions, reproduced with permission.^[^ ^93^ ^]^ Copyright 2021, Springer Nature. F) In situ NMR flow cell, reproduced under the terms of the CC BY 4.0 license.^[^ ^240^ ^]^ Copyright 2020, EDP Sciences. G) Vibrational spectroscopy: (left) Combination of vibrational (IR/Raman)spectroscopies with other techniques using commercially available cells for in situ/operando studies. Reproduced under the terms of the CC BY 4.0 license.^[^ ^232^ ^]^ Copyright 2023, Elsevier. (right) Operando reactor‐cell with simultaneous transmission FTIR and Raman characterization (IRRaman) for catalytic studies. Reproduced with permission.^[^ ^241^ ^]^ Copyright 2020, American Chemical Society.

When studying catalytic reactions in real‐time, however, it is imperative to have full control about the ambient conditions the materials are exposed to, including temperature, pressure, electric/magnetic fields, guest molecules, to name a few. Here, the design of in situ and operando cells is crucial for accurate and reliable spectroscopic studies of reticular materials and an ongoing field of research and engineering. These cells must be carefully designed to replicate real‐world operational conditions while maintaining compatibility with various spectroscopic techniques, such as IR, XAS, or Raman. Key considerations include ensuring uniform temperature and pressure control, allowing for precise gas or liquid flow, and minimizing interference from the cell materials themselves. Additionally, the cell design should facilitate the integration of multiple techniques at once to simultaneously monitor structural, kinetic, and dynamic changes within the material. Achieving these criteria allows for both qualitative and quantitative insights into the catalytic processes, enabling a comprehensive understanding of material behavior under working conditions. In **Figure** **8** displays various in situ and operando cell designs used to study catalytic processes. As this review does not attempt to give a comprehensive overview, we refer for more detailed information on cell design and modeling to the following references.^[^ ^231^, ^232^, ^233^ ^]^

#### 3.4.1 Single‐Atom and Single‐Site Catalysts

Single‐atom catalysts (SACs) feature isolated active sites, typically individual metal atoms, dispersed on support or within a reticular framework. In reticular materials, these atoms are anchored to the nodes, coordinated to organic linkers, or stabilized at defect sites within the framework (MOFs^[^ ^18^, ^271^ ^]^ and COFs^[^ ^3^ ^]^). While SACs are characterized by isolated atoms visualized using advanced microscopic techniques, the exact coordination environment and bonding between the metal and the support are often not fully understood. This differentiates SACs from single‐site catalysts (SSC), which possess uniform, well‐defined active sites but are not restricted to single atoms.^[^ ^272^ ^]^ The concept of single‐site catalysis originates from molecular organometallic chemistry, where precisely designed coordination environments around each metal center produce nearly identical active sites with uniform reactivity, spectroscopic signatures, and catalytic performance.^[^ ^3^, ^18^, ^271^, ^272^ ^]^ SACs and SSC offer unique opportunities for precise catalytic control and enhanced selectivity. Studies using vibrational spectroscopy and XAS revealed whether SACs remain intact during reactions and identified the oxidation states and local environments of active sites. Notably, UiO‐67 with its bipyridyl linker offering ion coordination, and UiO‐66, with the potential for anchoring on Zr‐clusters, are often used as substrate for SAC.

For instance, Braglia et al. utilized operando quick‐XAS to study the structural and electronic evolution of Pt‐SACs within UiO‐67 during H_2_ temperature programmed reduction (TPR).^[^ ^242^ ^]^ The study revealed that under specific conditions, two types of catalytically active sites could be formed: isolated Pt complexes and Pt nanoparticles (Pt‐NPs). This finding underscores the importance of controlling synthesis and activation conditions to tune the nature of active catalytic sites. Rojas‐Buzo et al. investigated the catalytic activity of Cu single‐site catalysts supported on defective UiO‐67(Ce) for CO oxidation.^[^ ^243^ ^]^ In situ DRIFTS and operando XAS revealed the presence of both Ce(III) and Cu(I) sites during the reaction. The defect‐engineered Cu‐UiO‐67(Ce) catalyst demonstrated enhanced catalytic activity. In addition, the electronic and structural properties of Cu‐SACs supported on UiO‐66 during the preferential CO oxidation (PROX) reaction were studied.^[^ ^245^ ^]^ The increase in reaction temperature led to the formation of Cu(I)‐like states and partial reduction of ZrO_x_ nodes, which correlated with decreased PROX selectivity. In situ NAP‐XPS, UV–vis DRS, and XANES data suggested an interface‐mediated charge transfer from ZrO_x_ to Cu sites, influencing catalytic performance. Ma et al. elucidated the local structure of atomically dispersed Cu sites and structural defects in UiO‐66 during NO_2_ reduction.^[^ ^273^ ^]^ In situ EPR spectroscopy and other complementary techniques proved that monomeric Cu(II) is reduced to Cu(I) and provided insights into the Cu‐substrate interactions, with insights into the catalytic performance of both Cu/UiO‐66 and its NH_2_‐functionalized analog. A mechanistic study of CO oxidation using UiO‐66(Cu) revealed the active site and reaction intermediates, identifying the rate‐limiting steps and the changes in oxidation/spin states during the reaction using a combination of XANES, EPR, and DRIFT spectroscopy.^[^ ^274^ ^]^ The study highlighted the continuous dissociation of adsorbed O_2_, with a specific O atom connecting the Cu center to a neighboring Zr(IV) ion as a critical part of the reaction cycle. The insights provided by in situ and operando spectroscopies were crucial for understanding the catalytic process at a molecular level.

A new method, termed Ni‐AIM, was developed for the high‐density incorporation of Ni ions into the nodes of NU‐1000 MOFs using atomic layer deposition.^[^ ^244^ ^]^ In situ XAS studies confirmed that the Ni(II) oxidation state was retained after H_2_ activation at 200 °C. The Ni‐AIM catalyst demonstrated high efficiency in gas‐phase hydrogenation reactions. Moreover, in situ XAS provided detailed insights into the electronic and geometric structure of the Ni sites during activation.

#### 3.4.2 Thermocatalysis

In situ and operando techniques act as windows into the dynamic world of thermocatalytic reactions in reticular materials by allowing real‐time observation of structural and electronic changes during reactions. In particular, UiO‐67 (with its ability to coordinate metal ions to its linker that can further form into nanoparticles) is popular as thermocatalyst. Methods like XAS and NAP‐XPS allow researchers to track the evolution of these metal sites and nanoparticle formation, revealing their ionic or metallic character as well as their dynamic shifts in oxidation states and coordination environments, giving insights into the active sites.

##### Hydrogenations

The first study on gas‐phase catalytic activity occurring at the metal node was performed using in situ NAP‐XPS and DFT calculations on bimetallic (Cu_x_Rh_1−x_)_3_(btc)_2_ during propylene hydrogenation.^[^ ^246^ ^]^ The results demonstrated that the Rh sites within the MOF maintained their oxidation state during catalysis, contributing to the material's stability and catalytic performance. Additionally, the Cu ions played a crucial role in stabilizing the MOF framework, preventing the incorporated Rh(II) from being reduced to metallic Rh under reaction conditions. In situ PXRD and XAS were used to monitor the formation of Pd nanoparticles within a functionalized UiO‐67 MOF for ethylene hydrogenation into ethane.^[^ ^247^ ^]^ The activation process involved reducing Pd(II) ions in hydrogen, which led to their detachment from the MOF linkers and subsequent growth into nanoparticles within the MOF cavities. Operando XAS and IR spectroscopy revealed the formation of palladium carbide species during the reaction, particularly in the presence of ethylene. This study highlighted the UiO‐67 MOF's ability to maintain the small size of Pd NPs, preventing sintering and ensuring stable catalytic performance. The role of the UiO‐67 framework in the hydrogenation of CO_2_ to methanol was elucidated using a combination of steady‐state kinetic measurements, H/D‐ and ^13^C/^12^C Steady‐State Isotopic Transient Kinetic Analysis (SSITKA), and operando FTIR spectroscopy.^[^ ^248^ ^]^ These methods, supported by DFT calculations, revealed the interactions between Pt–Zr–MOF, which are crucial for methanol formation. The findings provided unprecedented insights into the interplay between the metal and MOF components.

##### Oxidations

A comprehensive operando study using XAS and XES, Resonant Inelastic X‐ray Scattering (RIXS), and PXRD revealed the structural and electronic dynamics of MIL‐100(Fe) during the direct methane to methanol conversion using O_2_ as an oxidant.^[^ ^192^ ^]^ This study highlighted the role of open Fe(II) sites in facilitating the reaction. In addition, it was shown that activity of MIL‐100(Fe) may be regenerated by thermal treatment, as demonstrated for two consecutive catalytic cycles. Operando NAP‐XPS studies compared the performance of Pt catalysts supported on UiO‐67 MOFs and ZrO_2_ in CO oxidation.^[^ ^41^ ^]^ The findings revealed that the Pt metal phase, rather than its oxides, was the active phase for catalysis. The study also highlighted the superior sinter‐resistance of Pd nanoparticles supported on the UiO‐67 MOF.^[^ ^41^ ^]^ A combined NAP‐XPS and HR‐STEM study examined PtNi BNPs supported on UiO‐67 catalysts for CO oxidation.^[^ ^249^ ^]^ The findings indicated that the active phase responsible for the observed catalytic activity was a NiO_x_‐Pt structure, where subsurface Ni oxides segregated to form a thin NiO_x_ layer on the Pt surface. The discovery of this core‐shell structure stabilized in UiO‐67 provides new insights into the compositional and structural effects of such bimetallic nanoparticles on catalytic oxidation.

##### Other Conversions

In situ UV–Vis, FTIR and EPR spectroscopy, combined with DFT simulations, identified a chelating N_2_O^2−^ intermediate during the gas‐solid reaction of nitric oxide with Cu(I)‐ZrTpmC* MOFs (TpmC* = 1,1′,1′′‐methanetriyltris(3,5‐dimethyl‐1*H*‐pyrazole‐4‐carboxylic acid)).^[^ ^250^ ^]^ This intermediate is crucial for understanding the reductive coupling of nitric oxide to nitrous oxide at bimetallic sites, offering mechanistic insights that are compatible with enzymatic processes in heme‐copper oxidases and nitric oxide reductases.

Chemical conversions using reticular materials are not limited to gas‐solid interactions, but in situ and operando studies have also been performed in solid–liquid phase.^[^ ^275^ ^]^ One example is the detailed study on the mechanism of the Heck C–C coupling reaction, facilitated by Pd@MOFs using operando XAS and PXRD.^[^ ^251^ ^]^ A custom reactor for operando XAS and PXRD enabled real‐time analysis of chemical reactions and crystallographic changes under solvothermal conditions using synchrotron‐based techniques. These techniques, alongside TEM and kinetic studies using ^1^H NMR, enabled tracking of the transformation of palladium species from their active state to deactivation. This data supported the development of a flexible reaction mechanism model.

Expanding the use of advanced operando techniques, such as NAP‐XPS, will be essential for uncovering the mechanisms that govern metal‐support interactions. Recent advancements in NAP‐XPS are particularly remarkable and have achieved nanosecond time resolutions and ambient pressures up to 1 atm, making it more effective for investigating real‐world catalytic conditions.^[^ ^276^, ^277^ ^]^ Studying catalytic reactions under high‐resolution microscopy, as only recently on Ni nanoparticles, in beam‐sensitive reticular materials is a formidable challenge for microscopists.^[^ ^239^ ^]^ Additionally, investigating the application of these findings to other catalytic processes, including those involving solid‐liquid interfaces, could lead to broader applications of MOFs in chemical conversions. Moreover, operando Raman and infrared imaging can map the spatial distribution of active sites and intermediates, helping to understand heterogeneities in catalytic activity.^[^ ^108^, ^110^, ^210^, ^278^ ^]^

#### 3.4.3 Electrocatalysis

In situ and operando spectroscopy also come into play to investigate the structural, electronic, and catalytic behavior of MOFs and COFs in electrocatalytic applications.^[^ ^11^, ^279^, ^280^ ^]^ They have advanced our understanding of reactions like hydrogen evolution (HER), electrocatalytic CO_2_ reduction (CO_2_RR), oxygen reduction (ORR), and oxygen evolution (OER) have been investigated. They reveal oxidation states of active sites, identify reaction intermediates and pathways, track changes during catalytic reactions, and elucidate the coordination environment within MOFs, with particular attention towards porphyrin‐based MOFs. A considerable amount of research has focused on copper‐ and cobalt‐based systems.

##### State of the Active Site: OER

UV–vis spectroelectrochemistry studies on Mn‐MOFs revealed shifts in the absorption spectrum indicative of changes in coordination environments, which are directly related to the redox processes of Mn(II)/(III).^[^ ^43^ ^]^ UV–vis spectroelectrochemistry coupled with Raman spectroscopy, revealed that cyclic voltammetry (CV) induces a dramatic and irreversible morphological transformation in ZIF‐67 from cubes to irregular spheres. This change is associated with the progressive disruption of Co(II)–organic linker coordination, leading to the formation of Co(OH)_2_ and its subsequent oxidation to CoOOH phases, which serve as active sites for OER.^[^ ^252^ ^]^ Operando Synchrotron Radiation‐FTIR (SR‐FTIR) and XAS studies on lattice‐strained NiFe MOFs identified high‐valence Ni(IV) species and crucial *OOH intermediates during ORR and OER. These findings, enabled by advanced synchrotron‐based techniques, suggest that lattice strain can be used to modulate the electronic structure of active sites.^[^ ^253^ ^]^

##### State of the Active Site: CO_2_RR

In situ and operando XAS studies on Cu‐complex materials, namely Cu(II) phthalocyanine (Cupc), HKUST‐1, and Cu(II) 1,4,8,11‐tetraazacyclotetradecane chloride ([Cu(cyclam)]Cl_2_), identified the active sites for CO_2_ reduction to CH_4_ and provided insights into the coordination environment of Cu clusters. The study showed that Cu(II) phthalocyanine undergoes reversible structural changes to form metallic Cu clusters, while HKUST‐1 and [Cu(cyclam)]Cl_2_ form larger, less active Cu nanostructures.^[^ ^40^ ^]^ Another study varied the asymmetric local atomic structure and oxidation state of Cu dimers (established by EPR spectroscopy) in HKUST‐1 and studied the formation of Cu‐clusters during CO_2_RR using in situ XAS.^[^ ^254^ ^]^ They found that the presence of undercoordinated Cu sites promoted higher Faradaic efficiency towards ethylene.

In situ FTIR and XPS studies elucidated the catalytic mechanism in polyoxometalate‐based MOFs (POMOFs) for photo‐ and CO_2_RR. The Fe‐POMOF exhibited high selectivity for methane during electrocatalytic CO_2_ reduction and superior Faradaic efficiency for CO. In situ spectroscopies identified the mechanism (which involved the transfer of photogenerated electrons) and the roles of single metal sites and clusters.^[^ ^255^ ^]^ Another study presented a microwave‐assisted synthesis of a Ni‐based single‐atom catalyst (SAC) for CO_2_ electroreduction, achieving a CO partial current density of 1.06 A/cm^2^ with a CO Faradaic efficiency of 96%. In situ NAP‐XPS and ATR‐FTIR studies on this Ni‐doped ZIF‐8 revealed that defect sites in the MOF play a critical role in CO_2_ adsorption and activation responsible for the remarkable CO Faradaic efficiency in CO_2_RR.^[^ ^256^ ^]^

A MOF‐functionalized gas diffusion electrode (GDE) design was investigated for its ability to produce C_2_H_4_ at high rates and with high selectivity during CO_2_RR.^[^ ^257^ ^]^ Combined electroanalysis and operando XAS demonstrated that the MOF‐induced organic layers increase the local CO_2_ concentration near the active sites of Cu catalysts, which in turn improves the efficiency of CO_2_ electroreduction. In another study, operando XAS and in situ IR studies on Au nanoneedles embedded in the PCN‐222 MOF demonstrated that Cu(II) centers in the MOF framework remained largely stable during CO_2_RR. The XANES and EXAFS spectra confirmed this stability, contrasting with the reduction of Cu centers in other related MOFs, and highlighted the importance of structural stability in maintaining catalytic performance.^[^ ^258^ ^]^

##### Electroactive Ligand

While the aforementioned examples focus on metal nodes as the electrocatalytic centers, other studies have stabilized active sites within the linkers, such as porphyrins or cobaloximes. Here, UV‐Vis studies on a MOF consisting of cobaloximes (UU‐100(Co))) coordinated to zirconium‐oxo clusters revealed that the molecular integrity of cobaloxime linkers remains intact after electrolysis, demonstrating potential for long‐term electrochemical HER.^[^ ^262^ ^]^ ATR‐IR spectroscopy was employed to study a CO_2_ reduction catalyst featuring single‐atom Mn centers within a bipyridyl‐based COF.^[^ ^259^ ^]^ The catalyst demonstrated a low CO_2_RR onset potential and high current densities, with the lack of Mn dimer formation linked to improved catalytic performance.

In situ XAS on a redox‐active COF (tapa‐ope) with Co(II) grafted sites, demonstrated that the Co(II) centers undergo a reversible reduction during CO_2_RR. This process was correlated with a high Faradaic efficiency for ethanol production, supported by in situ FTIR spectroscopy, which identified the CHO* intermediate as critical in forming C_2_ products.^[^ ^260^ ^]^ Similarly, UV‐Vis spectroscopy on a cobalt porphyrin‐based COF under electrochemical conditions showed changes associated with Co(II)/Co(I) reduction, resulting in high Faradaic efficiency and significant stability in CO_2_ reduction to CO. XAS data further emphasized the COF's role in preserving the electronic structure of the catalytic centers.^[^ ^261^ ^]^

##### Sodium‐ and Lithium‐Ion Batteries

Reticular materials including MOFs and COFs have generated great interest in energy storage fields especially batteries, because the ordered porous frameworks can offer a fast‐ionic transportation and storage path without large volume variation.^[^ ^281^ ^]^ The behavior of Fe_2_(dhbq)_3_ (dhbq = 2,5‐dihydroxybenzoquinone) as an electrode material in sodium‐ion batteries was investigated through a combination of operando and ex situ methods, including FTIR, Raman, XANES, and Mössbauer spectroscopy, supplemented by DFT calculations. This study provided a detailed understanding of the structural changes and redox mechanisms occurring during battery charge and discharge cycles.^[^ ^282^ ^]^

In situ PXRD and operando XAS were employed to study the electrochemical performance of MIL‐101(Fe) as a lithium‐ion battery electrode, revealing that the Fe(III)/Fe(II) redox couple is electrochemically active during lithiation and de‐lithiation cycles.^[^ ^283^ ^]^ The operando XAS data showed the reversible changes in the Fe K‐edge during cycling, although relaxation of Fe(II) to Fe(III) led to irreversible lithium accumulation that reduced the material's capacity retention. Ex situ EXAFS further supported the computational findings, suggesting that the replacement of Cl^−^ ions with solvent molecules during lithiation contributes to structural instability and poor cycling performance.

Tp‐Azo‐COF with dual active sites of C═O and N═N was proposed as a novel anode material and successfully applied in Li‐ion batteries.^[^ ^284^ ^]^ Zhao et al. explained the lithiation mechanism of a carbonyl‐rich Tp‐Azo‐COF applied in lithium‐ion batteries (LIBs) by combining the theoretical approach with operando XPS and in situ FTIR analysis. Vibrational spectroscopic results revealed the reversible interactions between Li(I) ions and the C═O and N═N active sites, that Li‐O and Li‐N bonds were formed during Li(I) insertion. XPS further confirmed the reversibility of these redox processes, highlighting the dual redox‐activity, stability, and high cycling performance of Tp‐Azo‐COF.

These studies revealed critical information about the oxidation states, coordination environments, and structural stability of active sites and highlights the importance of lattice strain, defect sites, and metal‐ligand interactions in enhancing catalytic performance. Inspiration for improved spatial or time‐resolved methods can be drawn from the Roldán Cuenya group employing soft X‐ray ptychography, time‐resolved electron and X‐ray microscopy, or Bragg coherent diffraction imaging.^[^ ^285^, ^286^, ^287^, ^288^ ^]^

#### 3.4.4 Photocatalysis

Reticular materials have demonstrated significant photocatalytic activity in applications such as water splitting and CO_2_ photoreduction.^[^ ^14^, ^289^, ^290^ ^]^ These materials can be categorized into intrinsically photoactive reticular frameworks, where the linker or cluster itself is photoactive, and materials that serve as hosts for incorporated photosensitizers. In situ and operando spectroscopy have been crucial in uncovering the mechanisms, identifying active sites, and tracking reaction intermediates in these processes, providing real‐time insights into the electronic states and coordination environments during photocatalysis.

##### Reticular Materials as Support with Incorporated Photosensitizer

Photosensitizer can be coordinated to/supported in reticular materials opening a new field for photochemistry. In situ XAS spectroscopy was utilized to investigate the photocatalytic behavior of a Co/Fe mixed‐node MOF‐74, incorporating the [Ru(bpy)_3_]Cl_2_ photosensitizer.^[^ ^264^ ^]^ The study revealed that both Co and Fe centers, under reduced states, serve as catalytic active sites for HER. The accumulation of these species causes an induction period before stable H_2_ production, with changes in Co−O bond distance and Fe center geometry playing a crucial role in the photocatalytic process. Co‐Ru‐bpy was incorporated into the linker of UiO‐67 and the catalyst demonstrated exceptional light‐driven H_2_ production.^[^ ^265^ ^]^ In situ XAS and optical transient absorption spectroscopy identified the formation of key Co(I) intermediate species after ultrafast charge transfer and pinpointed the rate‐limiting step in the catalytic cycle. Re(bpy)(CO)_3_Cl was incorporated into a 2D triazine COF, which exhibited superior CO_2_ reduction activity compared to its homogeneous counterpart.^[^ ^266^ ^]^ In situ UV‐Vis diffuse reflectance spectroscopy revealed three key intermediate species responsible for charge separation, induction period, and the rate‐limiting step in CO_2_ reduction.

##### Linker or Cluster as Photoactive Species

In situ XAS at the Zr and Cu K‐edges indicated that photocatalytic CO_2_ hydrogenation in Cu‐UiO‐66‐NH_2_ is facilitated by charge transfer between Zr and Cu centers.^[^ ^267^ ^]^ The reduction of Cu^2+^ to Cu^(2‐δ)+^ states and the corresponding changes in Zr valence provided evidence for charge transfer between Zr and Cu. Combined in situ EPR and NAP‐XPS studies revealed the photoinduced electron transfer pathway in porphyrin‐based Zr‐MOF during NIR‐light‐driven CO_2_ reduction.^[^ ^268^ ^]^

##### Investigation of Reaction Intermediates

Operando FTIR spectroscopy was applied to study isoreticular MOFs with different organic linkers, showing their potential as photocatalysts for selective gas‐phase photooxidation of propylene.^[^ ^269^ ^]^ The study demonstrated that tuning the organic linkers allowed for the adjustment of the band gap.

A 3D SHINERS platform was developed to monitor reactive oxygen species (ROS) generation in MOFs such as ZIF‐67, ZIF‐8, and UiO‐66 under sunlight irradiation.^[^ ^270^ ^]^ The study found that ZIF‐67, with the smallest band gap, exhibited the fastest superoxide anion generation rate and the best photocatalytic performance, which was confirmed through dynamic Raman spectroscopy. Operando EPR studies on two donor–acceptor COFs showed their application as photoinitiators for visible light‐induced free radical polymerization, demonstrating the versatility of COFs in photocatalytic processes.^[^ ^56^ ^]^

Future research in photocatalysis within reticular materials can be advanced through the integration of more sophisticated techniques: High‐resolution time‐resolved spectroscopy, such as ultrafast transient absorption can provide insights into charge dynamics at femtosecond timescales.^[^ ^36^, ^265^ ^]^ The arrival of X‐ray free‐electron lasers (XFELs) offered researchers sufficient time resolution to follow a catalytic reaction in real‐time, i.e., produce “movies” of a catalytic transformation. Femtosecond time‐resolved X‐ray spectroscopy measurements, using unique temporal and spectral characteristics of XFELs, can be used to probe short‐lived transient species, e.g., surface bond breaking mechanism.^[^ ^291^ ^]^ Typically, XFEL measurements employ a liquid jet to continuously refresh the sample and mitigate beam damage. However, the use of XFEL techniques for catalytic reactions, especially in gas‐solid interfaces, remains an area of active exploration with great potential for groundbreaking discoveries. Finally, combining correlative microscopy with in situ spectroscopy could enable the simultaneous observation of structural and electronic changes at specific sites, further enhancing our understanding of photocatalytic processes.

## 4 Challenges and Opportunities

In situ and operando spectroscopy have advanced our understanding of reticular materials through studies of material formation (Section 3.1), structural dynamics and stability (Section 3.2), defect engineering (Section 3.3), and catalytic processes (Section 3.4). Despite these achievements, important hurdles still exist, which must be addressed to unlock the full potential of reticular materials in practical applications. Challenges include the need for higher resolution and sensitivity in measurements, the difficulty of studying materials under operational conditions, and the complexity of interpreting data. We will also explore opportunities for integrating computational methods, expanding studies into liquid‐phase reactions, and combining advanced spectroscopic and imaging techniques to gain deeper insights into reticular material behavior.

### 4.1 Challenge 1: Resolution and Sensitivity

Significant strides have been made in improving the spatial, temporal and chemical/spectral resolution of in situ and operando techniques, essential for capturing reactions and responses of reticular materials. Unprecedent spatial resolution, for instance by High‐Angle Annular Dark‐Field STEM (HAADF‐STEM),^[^ ^111^ ^]^ has enabled observations of localized events, like defect formation and structural rearrangements at the atomic level. Likewise, advances in temporal resolution, through techniques like Transient Absorption Microscopy (TAM), offer new ways to monitor fast, transient phenomena such as electron transfer and carrier diffusion during catalytic processes.^[^ ^98^, ^292^ ^]^ Despite this progress, major challenges remain in fully capturing the functional dynamics across length and time scales during key processes like synthesis, disassembly, and catalysis.

For instance, tracking nucleation and growth at the molecular level during synthesis or observing charge recombination and diffusion during catalytic events requires all spatial, temporal, and chemical precision simultaneously, which current techniques struggle to achieve. The trade‐offs between spatial and temporal resolution in techniques like X‐ray Absorption spectroscopy (XAS) or vibrational spectroscopy (IR, Raman) leave gaps in our understanding of fast, localized processes. Furthermore, achieving the structural insights necessary during operation, such as under light or electric bias, is still difficult. To address these challenges, there is a need for hybrid techniques that combine high spatial, temporal, and chemical resolution, alongside advancements in instrumentation and theoretical models. This will allow for real‐time, multi‐scale monitoring of functional dynamics, including complex host‐guest interactions, catalytic processes, and molecular switching behaviors that govern material performance. By leveraging both experimental and computational tools, the potential of reticular materials can be more fully realized in applications such as catalysis and energy conversion.

### 4.2 Challenge 2: Accessibility of Techniques and Comparability of Results

Access to advanced instrumentation remains limited, especially for synchrotron‐based techniques, which are expensive and require specialized equipment. Custom‐built experimental cells are also often needed, tailored to specific materials or applications, further complicating the standardization of these techniques. The lack of standardized methods across facilities makes it difficult to compare results, highlighting the need for more structured reporting including a high level of detail on the employed sample interfaces and instrumentation.

Adopting standardized approaches could help improve the characterization of adsorption sites and catalytic behaviors in MOFs and COFs.^[^ ^293^ ^]^ Enhancing the precision of site characterization through advanced vibrational spectroscopy,^[^ ^39^, ^293^ ^]^ as is commonly done for probing acid sites in zeolites, could lead to significant improvements in the study of reticular materials. Standardized reporting of cell design and experimental setups would further facilitate more reproducible and comparable studies.

### 4.3 Challenge 3: Sample Compatibility with Measurement

The compatibility of MOFs and COFs with in situ and operando setups is another obstacle. These materials can sometimes degrade or behave differently when exposed to probing conditions, such as X‐ray radiation or elevated temperatures, leading to inaccurate data. For instance, in NAP‐XPS studies, the reduction of Cu(II) to Cu(I) in some MOFs is induced by the combination of X‐ray irradiation and heating.^[^ ^246^ ^]^ Furthermore, short high‐power pulses used in pump‐probe techniques are often prone to beam damage as well. This phenomenon underscores how external stimuli used for characterization, such as radiation and temperature, can inadvertently alter the system being studied, especially in reactive materials or systems that undergo dynamic processes. Similarly, optical spectroscopy techniques, which are crucial for studying photoactive processes in MOFs and COFs, can induce significant changes in the material's behavior. For instance, UV‐Vis absorption spectroscopy, widely used to monitor electronic transitions, may trigger photochemical reactions unrelated to the processes under investigation. These effects can lead to excited states or initiate catalytic cycles, altering the material's structural or electronic properties in ways that are not representative of operational conditions. In photocatalytic systems, the irradiation needed for measurement can promote electron transfer or induce other photochemical processes, which could interfere with the accuracy of real‐time analysis and highlight the need of blank measurements.

To mitigate these effects, several measures need to be taken depending on the employed spectroscopic technique and sample system: For X‐ray‐based assays, techniques like the use of graphene layers have shown promise in reducing the degradation caused by X‐ray exposure,^[^ ^294^ ^]^ as graphene layers can act as a protective barrier. In the case of light‐sensitive materials, pulsed or modulated light sources are effective. For instance, two‐photon excitation microscopy relies on fs‐pulsed lasers in the IR that deliver energy in brief, controlled bursts, reducing continuous exposure and thereby mitigating potential damage during measurements.^[^ ^295^, ^296^ ^]^ Future in situ setups should prioritize the integration of complementary experimental techniques to obtain accurate, real‐time insights while minimizing measurement‐induced effects. For example, combining non‐invasive optical methods with time‐resolved X‐ray and spectroscopic techniques could help differentiate the inherent dynamic behavior of these materials from artifacts introduced by the characterization process.

### 4.4 Challenge 4: Sustainability and Scalability

As the need for sustainable materials grows, addressing the scalability of MOFs and COFs, particularly for green technologies, is critical. While in situ and operando techniques have advanced our understanding of these materials, their potential for scalable production and environmentally friendly synthesis remains underexplored. Developing sustainable synthesis routes that reduce hazardous solvents and energy use is essential for scaling up these materials for industrial applications. In situ and operando techniques could help optimize the design, making, and performance of reticular materials employed for CO_2_ adsorption or light‐driven catalysis. However, many current in situ and operando techniques are costly, not widespread, and impractical for large‐scale use. To fully unlock their potential, we must innovate scalable, cost‐effective methods that maintain the high performance of small‐scale studies, fostering MOFs and COFs to contribute to carbon capture and renewable energy systems on large scales.

### 4.5 Challenge 5: Limitations in Computational Methods for Scalable Synthesis

While computational methods hold great potential for optimizing synthesis pathways and material properties, a significant challenge remains when interpreting the obtained experimental data by in situ and operando techniques. Current computational approaches lack reliable theoretical models that can effectively predict the behavior of MOFs and COFs on larger length and time scales. They often focus on simplified systems or individual dynamic molecules, and struggle to account for the complex, multi‐scale dynamics involved in synthesis, catalytic processes, and material stability. Translating basic thermodynamic and kinetic models to describe the complex landscapes of pressing applications like carbon capture, energy storage, and solar fuel generation remains a critical hurdle.

While these challenges—ranging from limitations in resolution and sensitivity to the complexities of studying and modeling of materials under operational conditions—pose significant obstacles, they also present clear opportunities for innovation. Recent advancements in instrumentation, data analysis, and experimental design are helping to overcome these hurdles, offering new ways to explore the dynamic behaviors of MOFs and COFs. In the following sections, we will explore how these innovations are opening up exciting possibilities for deeper insights and more efficient characterization of reticular materials.

### 4.6 Opportunity 1: Data Complexity and Computational Methods

One of the major challenges in in situ and operando spectroscopy is the complexity of data gathered from dynamic systems. Short‐lived active species often coexist with spectator species, making it difficult to distinguish between the two and leading to potential misinterpretation.^[^ ^277^, ^297^, ^298^ ^]^ This challenge is particularly pronounced when multiple species and reactions are occurring simultaneously, as is frequently the case in heterogeneous catalysis. Traditional methods may struggle to isolate the signals of weak intermediates or transient species from background noise, creating obstacles for a clear understanding of reaction mechanisms. Advanced data analysis techniques, such as Multivariate Curve Resolution‐Alternating Least Squares (MCR‐ALS) and Modulation‐Excitation Spectroscopy (MES), offer solutions to these issues.^[^ ^126^, ^232^, ^299^, ^300^, ^301^ ^]^ MES, for example, applies a periodic modulation to a specific experimental parameter—such as gas concentration or temperature—and uses phase‐sensitive detection to extract dynamic information, helping to isolate meaningful and relevant signals.^[^ ^302^ ^]^ These techniques are valuable for deconvoluting complex data, particularly in systems where multiple active species are involved and we advocate to make more use of them. However, it is important to recognize that even the most sophisticated data analysis methods cannot extract information that simply is not present in the raw data. As the saying goes, “garbage in, garbage out”—the quality of the data input fundamentally determines the quality of the insights derived. Therefore, careful experimental design and control remain crucial to ensure the collected data is as clean and reliable as possible before applying these advanced analysis methods.

To address these growing challenges, computational methods, particularly Machine Learning (ML) and data‐driven approaches, present significant opportunities for interpreting complex datasets.^[^ ^303^ ^]^ ML algorithms can be trained to predict reaction pathways, identify active sites, and recognize distinct spectral signatures, which greatly aids in deconvoluting overlapping signals from in situ and operando studies. These tools are becoming increasingly effective at detecting small, subtle changes during complex reactions, such as minor shifts in XAS spectra,^[^ ^278^, ^304^ ^]^ and have already demonstrated success when combined with experimental methods like microscopy.^[^ ^305^ ^]^

### 4.7 Opportunity 2: Operando Techniques for Liquid‐Phase Reactions

Many crucial synthesis and catalytic processes occur in liquid‐phase environments, where the strong interaction of light with solvents poses a significant challenge for commonly used in situ and operando techniques, which are more frequently applied to gas‐solid interface s. Particularly, in the IR and soft X‐ray regions strong solvent absorption, which can obscure the detection of key reaction intermediates. However, advancements in vibrational spectroscopy, NMR, and soft X‐ray XAS, combined with specially designed liquid‐phase reaction cells, offer potential for real‐time monitoring of these reactions. These techniques mitigate background absorption by optimizing the path length of the cell to minimize solvent interference^[^ ^278^ ^]^ or applying phase‐sensitive detection methods to enhance the signal‐to‐noise ratio.^[^ ^302^ ^]^ IR spectroscopy in ATR configuration is an established technique for material characterization but has proven particularly powerful to study solid–liquid interactions.^[^ ^129^, ^133^, ^306^, ^307^, ^308^ ^]^ In NMR, solvent suppression techniques can be applied to reduce the signal from the solvent, allowing a clearer focus on the solute's behavior. Future developments in cell design and instrumentation will allow us to understand underlying processes during synthesis and defect formation, especially in liquid environments where diffusion, solvation, and catalyst‐solvent interactions significantly affect the overall reaction dynamics.

### 4.8 Opportunity 3: Advanced Microscopy Techniques

Advances in microscopy techniques, such as tip‐enhanced techniques like Raman spectroscopy (TERS) and Scanning Transmission electron Microscopy (STEM),^[^ ^213^ ^]^ offer new possibilities for achieving nanometer‐scale spatial resolution, allowing detailed observations of structural and electronic changes during dynamic or catalytic processes. Latest developments in HAADF‐STEM and integrated Differential Phase Contrast STEM (iDPC‐STEM) have provided significant breakthroughs in the studies of porous materials. iDPC‐STEM has been effective in visualizing sorption‐induced topological changes in zeolites,^[^ ^213^ ^]^ while HAADF‐STEM has been used to track the formation of metal‐organic layers.^[^ ^111^ ^]^ Applying in situ STEM to MOFs and COFs would allow for atomic‐level observation of interactions with organic guest molecules, revealing subtle structural rearrangements during sorption. Combining light‐based spectroscopy with electron microscopy could improve understanding how electronic/vibrational transitions correspond to structural changes at the atomic scale. However, capturing such detailed insights, particularly under operando conditions, remains a challenge, representing a growing area of research.

Defect characterization remains a crucial area of research because defects significantly influence the properties of MOFs and COFs. While current characterization techniques have advanced significantly, challenges remain in achieving precise identification and control of these defects. Advanced spatially resolved methods, like Raman and fluorescence microscopy allow for in situ and operando measurements and have greatly enhanced our understanding of defects. However, these techniques still face challenges in distinguishing or identifying defect types, such as missing linkers or metal clusters.

Additionally, techniques like s‐SNOM (Scattering‐type Scanning Near‐Field Optical Microscopy),^[^ ^309^ ^]^ AFM‐IR (Atomic Force Microscopy Infrared Spectroscopy),^[^ ^107^, ^310^, ^311^ ^]^ and THz‐STM (Terahertz Scanning Tunneling Microscopy)^[^ ^312^, ^313^ ^]^ are pushing the limits of spatial and temporal resolution, allowing researchers to capture structural and chemical changes at the atomic level in real‐time. Combining ultrafast and nonlinear optics, with spectroscopy and nano‐probe imaging has become a recent front in the field. This approach enables the simultaneous achievement of nanometer spatial and femtosecond temporal resolution,^[^ ^314^ ^]^ and even atomic resolution imaging with THz‐STM,^[^ ^315^ ^]^ which are yet to be explored in the field of reticular materials.

Lastly, recent advancements in microscopy techniques are not limited to small‐scale materials. Microscopy studies on large crystal studies can elucidate diffusion pathways or deactivation processes, such as those seen in zeolites,^[^ ^316^, ^317^, ^318^, ^319^ ^]^ could also benefit from these high‐resolution imaging techniques.

### 4.9 Opportunity 4: Structural Changes Induced with Non‐conventional Triggers

Exploring non‐conventional stimuli such as pH, electric, and magnetic fields represent another promising opportunity. For example, pH changes have been shown to induce structural and optical modifications in certain MOFs, making them promising candidates for applications in environmental monitoring and sensing.^[^ ^320^, ^321^, ^322^ ^]^ Similarly, electric fields can influence the synthesis, alignment, and functional properties of MOFs and COFs, opening new avenues for electrochromic devices and other advanced materials.^[^ ^21^, ^323^, ^324^, ^325^, ^326^ ^]^ Magnetic fields offer yet another means of manipulating the orientation, magnetic behavior, and adsorption characteristics of these frameworks, further expanding their functional potential.^[^ ^20^, ^327^, ^328^, ^329^ ^]^

### 4.10 Opportunity 5: Studies on COFs and Amorphous MOFs

Expanding in situ and operando studies to COFs and amorphous MOFs^[^ ^330^, ^331^, ^332^ ^]^ which have been barely investigated in comparison to crystalline MOFs, offers rich opportunities. Despite the growing interest in amorphous MOFs, their formation processes remain poorly understood, presenting opportunities for techniques such as XAS to probe short‐range order and vibrational spectroscopy to explore linker‐cluster connectivity. The lower volume of literature on COFs is primarily due to their relative novelty, as well as their complexity in their synthesis and stability. By learning from MOFs and already existing COF structures, ML approaches will push the field and help in predicting structures with specific properties. Yet, their actual synthesis pathways are not well understood requiring more sophisticated in situ and operando techniques, that allow monitoring in liquid environment. Equally, they are required for benchmarking and quality assessment, as COFs can be more fragile under operational environments. Hence, COFs often require more precise conditions, making them challenging for in situ studies.

In summary, while several challenges remain in the application of in situ and operando spectroscopy to MOFs and COFs, advances in both experimental and computational methods offer exciting opportunities. The integration of new techniques, computational tools, and stimuli promises to further unlock the potential of these materials for catalysis, sensing, and other advanced applications.

## 5 Conclusion

This review highlights the remarkable advancements in in situ and operando spectroscopic techniques for studying reticular materials, such as metal‐organic frameworks and covalent organic frameworks. These methods have significantly enhanced our understanding of material formation, structural dynamics, defect formation, and catalytic processes. In situ and operando techniques have provided real‐time insights into the molecular‐level transformations and have enabled researchers to observe dynamic processes under operational conditions, bridging the gap between theoretical predictions and practical applications.

Despite these advancements, several challenges remain. Current limitations in spatial and temporal resolution, as well as sensitivity, hinder the ability to fully capture the rapid and localized events that define the behavior of reticular materials. Furthermore, many of the operando studies to date have been conducted under simplified or mild conditions that do not fully replicate the demanding environments of industrial processes, particularly for high‐pressure, high‐temperature, and liquid‐phase reactions. The development of more robust experimental setups, including advanced liquid‐phase reaction cells and high‐pressure reactors, will be essential for moving the field forward.

To move forward, developing higher‐resolution techniques capable of capturing fast and localized events is critical. In particular, new liquid‐phase reaction cells that minimize solvent interference and advanced high‐temperature, high‐pressure reactors are needed to replicate more industrially relevant conditions. Moreover, the integration of computational tools, especially machine learning, will be essential for managing the vast and complex datasets produced by in situ and operando studies. This combination of enhanced experimental setups and data analysis will allow for a deeper, more precise understanding of how reticular materials behave in real‐world applications.

In conclusion, while in situ and operando spectroscopy have revolutionized the study of reticular materials, future research must focus on overcoming technical limitations and extending these techniques to more extreme and complex environments. By addressing these challenges, we can unlock the full potential of reticular materials for industrial catalysis, energy storage, and beyond.

## Conflict of Interest

The authors declare no conflict of interest.

## Supporting information



Supporting Information
