# Supplementary material for: Advancements in Understanding the Physicochemical Properties of Reticular Materials: An In Situ and Operando Spectroscopic Perspective
Source: Adv Mater. 2025 Feb 24;37(52):2415135. doi: 10.1002/adma.202415135 (PMC12747497; doi:10.1002/adma.202415135)
Supplement: Supplementary file 1 — Supporting Information [file ADMA-37-2415135-s001.pdf]

ADVANCED MATERIALS

Supporting Information

for *Adv. Mater.*, DOI 10.1002/adma.202415135

Advancements in Understanding the Physicochemical Properties of Reticular Materials: An In Situ and Operando Spectroscopic Perspective

Bettina Baumgartner, Anna Wach, Xinwei Ye, Evelyn Ploetz and Bert M. Weckhuysen*

Supporting information for

Advancements in Understanding the Physicochemical Properties of Reticular Materials: An *In situ* and *Operando* Spectroscopic Perspective

Bettina Baumgartner[1,*], Anna Wach[2,3], Xinwei Ye[4], Evelyn Ploetz[5], and Bert M. Weckhuysen[4]

1 Van't Hoff Institute for Molecular Sciences, University of Amsterdam, Science Park 904, 1098 XH Amsterdam, the Netherlands, b.baumgartner@uva.nl

2 SOLARIS National Synchrotron Radiation Centre, Jagiellonian University, Krakow, Poland, anna.wach@uj.edu.pl

3 Institute of Physical Chemistry, Polish Academy of Sciences, 01-224 Warsaw, Poland

4 Debye Institute for Nanomaterials Science and Institute for Sustainable and Circular Chemistry, Utrecht University, Universiteitsweg 99, 3584 CG Utrecht, The Netherlands; x.ye1@uu.nl, b.m.weckhuysen@uu.nl

5 Department of Chemistry and Center of NanoScience (CeNS), Ludwig-Maximilians-Universität München, Butenandtstr. 5-13, 81377 Munich, Germany, evelyn.ploetz@lmu.de;

Content

1 Spatially resolved techniques 2

2 Supplemental table for Figure 2: Literature statistics 9

3 Abbreviations 10

4 References 13

1 Spatially resolved techniques

Table S1 Overview spatially resolved *in situ* and *operando* techniques used to study reticular materials

<table>
<tr><th>Technique</th><th>Wavelength</th><th>Available information</th><th>Experimental configurations</th><th>Experimental limitations</th><th>Time resolution</th><th>In situ application</th><th>Advantages</th><th>Useful textbooks reviews steady state, and in situ</th></tr>
<tr><td>Fluorescence microscopy</td><td>UV, Vis; typically 300-800 nm</td><td>• Spatial distribution of fluorescent molecules
• Localization of specific groups/ components
• Dynamics of framework motion and molecular interactions
• Sensing of local environment with nm resolution via spectra, lifetime and anisotropy</td><td>• Widefield
• TIRF: total-internal reflection
• Lattice light-sheet microscopy
• CLSM: Confocal laser scanning microscopy</td><td>• Diffraction-limited
• Photobleaching
• Phototoxicity
• Requires labeling or autofluorescence
• Potential background from autofluorescence
• Limited penetration depth by thick samples</td><td>• Millisecond to second for Widefield / TIRF and light-sheet
• ns to µs with TCSPC and APD for CLSM</td><td>• Real-time imaging of reticular materials
• Molecular interactions and framework changes
• Diffusion and adsorption of guest-molecules
• Study of defects confinement or catalytic reactions</td><td>• Noninvasive
• Little disruptive to sample, if linker or IBU are luminescent
• High specificity, high SNR and sensitivity
• Wide range of SR imaging available
• Suitable for dynamic and live-cell imaging</td><td>Books:
• Advanced Fluorescence Microscopy[1]
• Super-Resolution, [2,3]
• Light-sheet[4]

Reviews:
• Confinement[5]
• Defects[6]
• Sensing[7–9]</td></tr>
<tr><td>Infrared microimaging</td><td>Mid-infrared; typically 2.5-25 µm</td><td rowspan="2">• Probes IR or Raman spectrum, respectively, of materials and guest molecules
• Concentration gradient determination
• Phase identification, crystallinity, stress /strain
• Structural heterogeneity</td><td>• Fourier-Transform
• Transmission, reflection, attenuated total reflectance modes</td><td>• Reduced spatial resolution (3-10 µm) compared to Raman
• Low sensitivity for thin or weakly absorbing samples
• Operates under ambient conditions</td><td>seconds to min</td><td rowspan="2">• Real-time imaging of reticular materials
• Monitoring of chemical changes, phase transitions, thermal properties at the microscale
• Spatial mapping of chemical species</td><td rowspan="2">• Simultaneous read-out of chemical and spatial information
• Non-destructive analysis
• Suitable for wide range of materials
• Fast mapping of large areas
• IR: sensitive to monitoring CO_2
• Raman: sensitive to monitoring water</td><td>Books[10]
Reviews[11]</td></tr>
<tr><td>Raman mapping</td><td>Visible to Near-Infrared (typically 400-1000 nm, depending on the laser used)</td><td>• Mapping with confocal setup with point and line scan
• Wide-field Raman
• TERS microscope
• Combination with AFM, IR or fluorescence</td><td>• Diffraction-limited
• Weak signal
• Longer acquisition time / high laser power
• Potential Fluorescence background</td><td>• Spectra: s to min
• Images: up to hours</td><td>Books[10,12,13]
Reviews[14–18]
Gas sorption[19]</td></tr>
<tr><td></td><td></td><td></td><td></td><td></td><td></td><td></td><td></td><td></td></tr>
</table>

Interferometric scattering microscopy (iSCAT)	Vis to NIR; 400-1000 nm	• High-sensitivity detection of nanoparticles or weakly scattering objects (subcellular structures, virions, nucleation seeds, proteins<br>• Sub-diffraction-limited resolution<br>• Information on particle size, position, dynamics, interaction at the nanometer scale	• Standard iSCAT setup based on WF system with laser illumination and interferometric detection<br>• Frequently combined with fluorescence and/or Raman modalities	• Diffraction-limited<br>• Highly sensitive to mechanical vibrational and environmental noise<br>• Requires precise alignment and calibration<br>• Complex interpretation of scattering objects	µs - ms	Real time tracking of nanoparticle dynamics, synthesis aggregation, and interactions in porous materials like MOFs, COFs, ZIFs	• Real-time monitoring of dynamic processes at the nanoscale<br>• label-free<br>• Tracking Resolution down to 10 nm<br>• ultrasensitive detection and high temporal resolution for observing fast processes	Books[20,21]<br>Reviews[22–25]
Dark-Field microscopy	Visible; typically 400-700 nm	• High contrast images of small features, edges, or boundaries<br>• Morphology and structural details of transparent specimen<br>• Detection of scattering centers, like nanoparticles, defects, material inclusions	• Standard optical microscopy with DF extension<br>• Frequently combined with Fluorescence and/or Raman modalities	• Diffraction-limited<br>• Lower resolution compared to confocal or SR microscopy<br>• Limited depth of field<br>• Nonspecific scattering contrast<br>• Not suitable for highly absorbent or thick samples that block light	ms to s depending on the light source and detector	• Label-free<br>• Noninvasive imaging with high contrast for transparent or light-scattering objects<br>• Simple, cost-effective setup<br>• Clear images of small particles and fine structures	• High spatiotemporal resolution<br>• Label-free	Books [26]<br>Reviews[27,28]
Stimulated Emission Depletion (STED) Microscopy	Visible to Near-Infrared (typically 500-800 nm)	• Confocal fluorescence imaging with high-spatial resolution beyond the diffraction limit	• STED with Confocal laser scanning microscope<br>• Dual-color or multi-color STED	• Limitations of fluorescence imaging<br>• Requires STED specific fluorophores<br>• Photobleaching or photodamage due to high-intensity depletion laser<br>• Complex setup and alignment	• ns - µs with TCSPC and APD	• Real-time imaging of processes with high spatial resolution<br>• Nanoscale mapping of guest-molecules, defects, catalytic sites including nano-confinement	• Super-resolved images down to 20-30 nm<br>• Noninvasive, live-cell compatible<br>• High specificity, high SNR and sensitivity	Books[29–32]<br>Reviews[33–36]

Structured Illumination Microscopy (SIM)	Visible to Near-Infrared (typically 400-700 nm)	• Widefield fluorescence imaging with high-spatial resolution beyond the diffraction limit	• SIM with Widefield fluorescence imaging	• Requires specific dyes compatible with SIM<br>• Lower depth penetration compared to confocal microscopy<br>• Computational reconstruction can be time-consuming and introduce artifacts	• Milliseconds to seconds per frame, depending on exposure time, scanning speed and re-construction	• Real-time imaging of dynamic processes<br>• High-resolution imaging of 2D/3D structures	• Super-resolved images down to 100-120 nm<br>• Suitable for live-cell imaging and dynamic studies with balance between resolution and imaging speed<br>• High specificity due to the use of fluorophores	Books[21]<br>Reviews[37–39]
Transient Absorption Microscopy (TAM)	Usually Near-Infrared (typically 200-1200 nm)	• Sensitive to changes in electronic states<br>• Monitoring of ultrafast processes, e.g. charge or energy transfer<br>• Dynamics of electronic states, incl. Exciton diffusion, charge separation and recombination<br>• Structural relaxation following photoexcitation	• Pump-probe setup<br>• Combined broad-band TA spectroscopy with microscopy<br>• Time-resolved TAM	• Diffraction-limited<br>• Limited penetration depth<br>• Photodamage	• fs to ps time resolution to monitor ultrafast dynamics<br>• ns to µs regime accessible on specialized setups	• Potential real-time tracking of photoinduced processes, like CT in photo-catalysis, guest sorption dynamics and changes in framework structure<br>• Combinable with operando conditions	• Super-resolved images down to 50 nm<br>• Ultrafast time resolution<br>• Label free<br>• Sensitive to materials with electronic transitions	Books[40]<br>Reviews[41–44]<br>Articles[45]
Single-Molecule Localization Microscopy (SMLM)	UV; Vis, typically 400-700 nm depending on the used dye	• Precise localization of dyes with nm precision<br>• Molecular organization<br>• Tracking of dynamic processes	• Widefield setups with stochastic binding/ switch-ing/ activation of dyes<br>• TIRF for near-field imaging<br>• emCCD or sCMOS cameras for single-molecule detection	• Photobleaching of dyes<br>• Requires dens labeling, probe specificity<br>• Requires compatible dyes<br>• Long acquisition times due to sequential data recording<br>• Limited penetration depth	Typically ms to s<br><br>Can reach µs for fast photo-switching and appropriate detectors	• Tracking of NPs / molecules during diffusion or sorption<br>• Real-time imaging of interactions, catalysis, defect formation<br>• High resolution for probing structural changes with nm precision	• Spatial resolution down to 10 nm<br>• Non-invasive<br>• Sensitive to rare or transient events<br>• High spatial precision<br>• Tracking of individual guests	Books[46,47]<br>Reviews[48–53]

Atomic-Force Microscopy (AFM)	Not applicable	• Surface topography and roughness<br>• Mechanical properties like stiffness, elasticity, friction, adhesion, magnetic forces and electric properties<br>• Material compos-ition at nanoscale	• Contact mode AFM for high-resolution topography<br>• Tapping mode<br>• Non-contact mode<br>• AFM in combination with Raman, IR and electrostatic force microscopy	• Limited to surface analysis<br>• Slow for large-scale scans<br>• Requires flat and clean surface<br>• Tip wear and contamination can affect resolution and accuracy	Millisecond to seconds per line scan, depending on scan speed and resolution<br><br>Typically steady-state imaging	• Real-time monitoring of surface reactions, mechanical property changes<br>• Studying the effects of external stimuli	• Nanoscale resolution<br>• Provides both topographical and material property information<br>• Non-destructive analysis, versatile	Books[54–56]<br>Reviews[57,58]
Secondary Ion Mass Spectrometry (SIMS)	Not applicable.	• Elemental composition<br>• Isotopic ratios<br>• Molecular fragments<br>• Depth profiling<br>• Lateral resolution<br>• Species mapping	• Different ions as excitation source<br>• Static / Dynamic<br>• Time-of-Flight-SIMS<br>• Magnetic Sector SIMS<br>• In situ liquid SIMS	• Surface damage<br>• Little quantitative<br>• Limited molecular analysis<br>• Vacuum required	Mainly static technique.	• Real-time monitoring of reactions, incl. nucleation, growth, catalysis<br>• Study of diffusion or ion exchange<br>• Surface monitoring	• Nanometer resolution<br>• High sensitivity (ppm/ppb level)<br>• Elemental / Isotope resolution with nm depth	Reviews[59,60]
Atom Probe Tomography (ATP)	Not applicable	• Composition and distribution of atoms, isotopes<br>• Distribution of grain boundaries, defects, interfaces	• Pulsed-laser APT	• Sophisticated sample design, tip<br>• Vacuum required for ionized atoms<br>• Limited to small volumes<br>• Destructive<br>• Low compatibility: organic material	• Mainly static technique<br>• Detection of ions on the µs timescale	• Probing of changes in reticular materials post-reaction, i.e. structural and compositional changes	• 3D atomic resolution<br>• Quantitative<br>• Sensitivity to light elements	Books[61,62]<br>Reviews[63,64]
Scanning Tunneling Microscopy (STM)	Not applicable.	• Atomic scale topography, electronic structure, surface morphologies<br>• Atomic, molecular arrangements, defect sites and surface reconstruction.	• STM in ultra-high vacuum (UHV) for atomic resolution<br>• Low-Temperature STM<br>• In situ STM for observing reactions	• Limited to conductive or semi-conductive samples<br>• Requires extremely stable and clean surfaces<br>• Highly sensitive to vibrations and environmental noise<br>• Typically operates under UHV or controlled environment	Milliseconds to seconds per image, depending on scan speed and area<br><br>Primarily used for steady-state imaging and spectroscopy	• Real-time observation of surface or cataly-tic reactions, sorption processes at the atomic scale<br>• Monitoring changes in electronic structure / surface morphology under varying conditions	• Atomic scale resolution<br>• Direct visualization of atomic and molecular structures<br>• Ability to study electronic properties	Books[54]<br>Reviews[65–67]

Infrared Scattering-type Scanning Near-Field Optical Microscopy (IR s-SNOM)	Infrared Scattering-type Scanning Near-Field Optical Microscopy (IR s-SNOM)	• Chemical composition, molecular vibrations, and identification of functional groups with nanoscale spatial resolution<br>• Localized absorption spectra<br>• Mapping of chemical heterogeneity at the nanoscale	• IR s-SNOM with AFM<br>• Combined FTIR and SNOM for spectra<br>• Tip-enhanced configuration for increased sensitivity	• Requires precise alignment and calibration of the light source and the tip / cantilever<br>• High sensitivity to experimental noise and tip-sample distance variations<br>• Surface information with limited penetration depth<br>• Limited to samples with flat or non-rough surfaces<br>• Challenging interpretation of near-field spectra	Millisecond to seconds, depending on scanning speed, laser modulation rate and detector sensitivity<br><br>IR s-SNOM: Primarily Steady-State imaging	• Real-time monitoring of chemical changes, surface reactions and phase transitions<br>• Mapping of functional groups and chemical distributions at the nanoscale<br>• Observation of sorption processes, reactions and interactions at material surface and interfaces	• Nanoscale spatial resolution with chemical specificity<br>• Non-destructive analysis for a wide range of matter<br>• Simultaneous topographic and chemical information<br>• Working under ambient and various environmental conditions.	Reviews[68,69]
Atomic Force– Infrared Microscopy (AFMIR)	Infrared (typically mid-IR, 2.5-25 µm or 4000-400 cm^{-1})		• AFM-IR with tunable IR laser<br>• Contact mode AFM combined with pulsed IR laser<br>• AFM-IR in tapping mode					Reviews[70–72]
Scanning Electron Microscopy (SEM)	Electron beam (0.5-10 nm; depending on acceleration voltage)	• Surface morphology, topography and composition<br>• High-resolution imaging of surfaces and near-surface features<br>• Elemental analysis with EDS<br>• Qualitative/semi-quantitative analysis of surface structure and composition	• Conventional SEM<br>• High-resolution SEM for improved spatial resolution<br>• SEM combined with EDS for. Elemental mapping<br>• Variable pressure or environmental SEM for imaging in low vacuum or *in situ* studies	• Limited to surface and near-surface information<br>• Requires conductive samples or coating of non-conductive samples<br>• Potential electron beam damage<br>• Requires vacuum that can impact the sample structure	Milliseconds to seconds depending on the detector and scanning speed	• Real-time monitoring of surface reactions, structural changes and degradation processes in porous materials<br>• Observation of sorption processes and their impact on material surfaces	• High spatial resolution for surface imaging<br>• Ability to combine imaging with elemental analysis<br>• Non-destructive surface imaging in most cases	Books[73,74]<br>Reviews[75–77]

Transmission Electron Microscopy (TEM)	Electron beam (0.005-0.1 nm; depending on acceleration voltage)	• High-res. Images of internal structures, crystal structure, phase identification, atomic structure, defects, grain boundaries and interfaces<br>• Electron diffraction patterns<br>• Chemical composition with EDS and EELS	• Conventional TEM<br>• High-resolution TEM for atomic-level imaging<br>• Combined with EDS for elemental mapping<br>• Aberration-corrected TEM<br>• *In situ* TEM with heating, cooling and gas/liquid cells for observing dynamic processes	• Requires thin samples < 100 nm<br>• Electron beam damage<br>• Complex interpretation of spectra<br>• High vacuum required<br>• Limited by speed of beam scanning, detector response and potential beam damage of the sample	Milliseconds to seconds depending on the detector and imaging mode	• Real-time observation of structural changes, phase transitions and chemical reactions and other processes<br>• Stability assessment<br>• Monitoring diffusion and sorption behavior of guest molecules.	• Atomic-level resolution for detailed structural analysis<br>• Ability for *in situ* studies under controlled environment	Books[78,79]<br>Reviews [75–77,80]
Electron Energy Loss Spectroscopy (EELS)	Electron beam (0.005-0.1 nm; depending on acceleration voltage)	• Chemical composition of sub-micron volumes<br>• Electronic structure, bonding information, oxidation states, elemental mapping	• EELS integrated with STEM<br>• EELS combined with EDS for comprehensive elemental and chemical analysis<br>• Aberration-corrected STEM-EELS for sub-Å resolution<br>• *In situ* measurements with heating, cooling or gas/liquid cells	• Requires thin samples < 100 nm<br>• Electron beam damage<br>• Complex interpretation of spectra<br>• High vacuum required<br>• Limited by speed of beam scanning, detector response and potential beam damage of the sample	Milliseconds to seconds in scanning mode	• Real-time analysis of changes in chemical composition, oxidation state, electronic structure during catalytic reactions, phase transitions and other processes<br>• Monitoring diffusion and adsorption of guest molecules.	• High spatial and energy resolution<br>• Ability to detect light elements (C/N/O) and differentiation between similar elements<br>• Information about electronic structure and bonding	Books[81,82]<br>Reviews[83]
Scanning Transmission Electron Microscopy (STEM)	Electron beam (0.005-0.1 nm; depending on acceleration voltage)	• High-res. Images of atomic structure, chemical composition, phase distribution<br>• Elemental mapping through EDS and EELS<br>• Atomic-level imaging of defects, interfaces and grain boundaries		• Sample preparation can be challenging, requiring thin sections < 100 nm<br>• Electron beam damage for MOFs and COFs<br>• Limited FOV<br>• High vacuum required	Milliseconds to seconds depending on the detector and imaging mode	• Real-time observation of structural changes, phase transitions and chemical reactions in reticular materials<br>• Monitoring of diffusion and	• Atomic-level resolution<br>• Ability to combine imaging with spectroscopy	Books[73,79]<br>Reviews [75–77,80,83]

						sorption of guest molecules		
Scanning Transmission X-ray Microscopy (STXM)	Soft X-Ray (typically 0.1-4nm, corresponding to photon energies in the range of 0.3-4 keV)	• Elemental composition, chemical state, binding environment, oxidation states<br>• Nanoscale mapping of chemical and structural heterogeneity<br>• Spatially resolved XANES<br>• NEXAFS spectra	• STXM with synchrotron radiation<br>• Combination with CAS<br>• Cryo-STXM<br>• *In situ* cells for studying reactions under controlled atmospheres	• Requires Synchrotron facility<br>• Limited to thin samples (< 100 nm)<br>• Low absorption contrast for certain elements<br>• Artificial environment due to high vacuum or selected atmosphere	• Seconds to minutes for full spectral imaging<br>• Milliseconds for fast scans with limited spectral range<br>• Limited scan speed, low detector sensitivity and synchrotron beamline availability	• Real-time monitoring of chemical reactions, phase transitions, and oxidation states at the nanoscale<br>• Mapping of heterogeneity in materials under various environmental conditions (e.g., pressure, temperature and gas flow)	• High spatial resolution down to 20-30 nm<br>• Elemental and chemical sensitivity with the ability to map oxidation states and chemical environment<br>• Suitable for wide range of materials	Books[84]<br>Reviews [85]
Transmission X-ray Microscopy (TXM)	Soft to Hard X-rays (typically 0.1-10 nm, corresponding to photon energies from ~0.1 keV to ~10 keV)	• High-resolution 3D imaging, spatially resolved tomography, nanoscale mapping<br>• morphological details, structural and chemical heterogeneity,<br>• elemental composition, chemical state, oxidation states, and density variations in complex materials.	• TXM with synchrotron radiation<br>• Cryo-TXM<br>• XAS-TXM for chemical states<br>• *In situ* TXM in cells for studying reactions under controlled conditions	• Requires Synchrotron facility<br>• Limited to thin samples (< 100 nm)<br>• Low absorption contrast for certain elements<br>• Artificial environment due to high vacuum or selected atmosphere	• Seconds to minutes for full 3D tomography<br>• Millisecond to second for single 2D projection images<br>• Limited scan speed, low detector sensitivity and synchrotron beamline availability		• High spatial resolution down to 30-50 nm in 2D<br>• 3D tomographic capabilities<br>• Detailed 3D structural information along with chemical and morphological data<br>• Suitable for wide range of materials	Books<br>Reviews [86,87]

2 Supplemental table for Figure 2: Literature statistics

Data was obtained by searching for "*operando*", "technique", "MOF" or "COF", "research article" in Web of Science and Scopus.

Search date: 16/09/2024

keywords: "in situ xx", research article (the results strictly contains "in situ xx")

		NMR	EPR	Raman	IR	UV-Vis	XAS	XPS
web of science	title	167	73	1040	869	110	104	160
	topic	797	324	5456	5054	961	492	881
Scopus	title	179	81	1092	957	111	100	169
	title, abstract, keywords	670	332	5312	4570	961	449	841

keywords: "operando xx", research article (the results strictly contains "operando xx")

		NMR	EPR	Raman	IR	UV-Vis	XAS	XPS
web of science	title	14	20	120	60	26	47	9
	topic	45	49	551	221	102	203	27
Scopus	title	13	18	117	59	26	47	7
	title, abstract, keywords	46	44	526	193	103	197	24

keywords: "in situ xx", COF, research article (the results strictly contains "in situ xx")

		NMR	EPR	Raman	IR	UV-Vis	XAS	XPS
web of science	topic	0	1	16	2	3	1	3
Scopus	title, abstract, keywords	0	1	16	2	3	1	4

keywords: "in situ xx", MOF, research article (the results strictly contains "in situ xx")

		NMR	EPR	Raman	IR	UV-Vis	XAS	XPS
web of science	topic	12	7	108	72	8	4	9
Scopus	title, abstract, keywords	8	7	95	62	9	3	11

keywords: "operando xx", MOF, research article (the results strictly contains "operando xx")

		NMR	EPR	Raman	IR	UV-Vis	XAS	XPS
web of science	topic	0	0	11	5	0	5	0
Scopus	title, abstract, keywords	0	0	11	4	0	4	0

3 Abbreviations

AC - Alternating Current
AFM - Atomic Force Microscopy
AFM-IR - Atomic Force Microscopy-based Infrared Spectroscopy
AP-HAXPES - Ambient Pressure Hard X-ray Photoelectron Spectroscopy
AP-XPS - Ambient Pressure X-ray Photoelectron Spectroscopy
ATP - Atom probe tomography
ATR - Attenuated Total Reflection
ATR-FTIR - Attenuated Total Reflection Fourier-Transform Infrared Spectroscopy
ATR-SEIRAS - attenuated total reflection surface-enhanced infrared absorption spectroscopy
azo-bpy - 3-azo-phenyl-4,4′-bipyridine
bdc - 1,4-benzene dicarboxylic acid
bme-bdc^{2-} - 2,5-bis(2-methoxyethoxy)-1,4-benzene-dicarboxylate
bpn - 1,4-bis(4-pyridyl)naphthalene
bpy - 4,4′-bipyridine
btc - benzene-1,3,5-tricarboxylate
CARS - Coherent Anti-Stokes Raman Scattering
CFM - Confocal Fluorescence Microscopy
COF - Covalent Organic Framework
CO2RR - electrocatalytic CO_2 reduction reaction
CUS - Coordinatively Unsaturated Sites
CV - Cyclic Voltammetry
dabco - 1,4-diazabicyclo-[2.2.2]octane
dhbq - 2,5-dihydroxybenzoquinone
dobdc - 2,5-dihydroxyterephthalic acid
dpni - *N*,*N*′-di(4-pyridyl)-1,4,5,8-naphthalenetetracarboxydiimide
DFT - Density Functional Theory
DR-UV-Vis - Diffuse Reflectance Ultraviolet-Visible Spectroscopy
DRIFTS - Diffuse Reflectance Infrared Fourier Transform Spectroscopy
DRS - Diffuse Reflectance Spectroscopy
DSC - Thermogravimetric-Differential Scanning Calorimetry
EELS - Electron Energy Loss Spectroscopy
EIS - Electrochemical Impedance
ePDF - Electron Pair Distribution Function
EPR - Electron Paramagnetic Resonance
EXAFS - Extended X-ray Absorption Fine Structure Spectroscopy
F_4bdc - tetrafluorobenzene-1,4-dicarboxylate
fum - fumarate
FLIM - Fluorescence Lifetime Imaging Microscopy
FTIR - Fourier-transform Infrared Spectroscopy
FTS - Fischer-Tropsch Synthesis
IBU - Inorganic building unit
ICP-AES - Inductively Coupled Plasma Atomic Emission Spectroscopy
INS - inelastic neutron scattering

iSCAT - Interferometric Scattering Microscopy
IR - Infrared Spectroscopy
IRRAS - Infrared Reflection Absorption Spectroscopy
H_3tpo tris-(4-carboxylphenyl)phosphineoxide
HAADF-STEM - high-angle annular dark-field STEM
HAXPES - Hard X-ray Photoelectron Spectroscopy
HER - Hydrogen Evolution Reaction
HERFD-XAS - High Energy Resolution Fluorescence Detected XAS
HERFD-XANES - High Energy Resolution Fluorescence Detected XANES
HR-STEM - High-Resolution STEM
iDPC-STEM - Integrated differential phase contrast STEM
LMCT - Ligand-to-Metal Charge Transfer
MAS-NMR - Magic Angle Spinning NMR
MAS-SS-NMR - Magic Angle Spinning Solid-State NMR
MOF - Metal-Organic Framework
ML - Machine Learning
MLCT - Metal-to-Ligand Charge Transfer
NAP-XPS - Near Ambient Pressure X-ray Photoelectron Spectroscopy
NASCA - Nanometer Accuracy by Stochastic Catalytic Reactions Microscopy
NEXAFS - Near-edge XAFS Spectroscopy
ndc 2,6-naphthalenedicarboxylic acid
NIR - Near Infrared
NMR - Nuclear Magnetic Resonance
NP – Nanoparticle
OER - Oxygen Evolution Reaction
ORR - Oxygen Reduction Reaction
PNBU - pre-nucleation building unit
PTIR - photothermal induced resonance
PXRD - Powder X-ray Diffraction
RIXS - Resonant Inelastic X-ray Scattering
ROS - Reactive Oxygen Species
RR - Resonance Raman
SAC - Single-Atom Catalyst
SALE - Solvent-Assisted Ligand Exchange
SAXS - Small-Angle X-ray Scattering
SEIRAS - Surface-enhanced Infrared Absorption Spectroscopy
SEM - Scanning Electron Microscopy
SIM - Structured Illumination Microscopy
SMLM - Single Molecule Localization Microscopy
SRS - Stimulated Raman Scattering
SS-ITKA - Steady-State Isotopic Transient Kinetic Analysis
SS-NMR - Solid-State NMR
s-SNOM - scattering-type Scanning Near-field Optical Microscopy
SR-FTIR – Synchrotron-Radiation FTIR
STED - Stimulated Emission Depletion Microscopy

**STEM** -	Scanning Transmission Electron Microscopy
**STEM-TEY** -	STXM-Total electron Yield
**STM** -	Scanning Tunneling Microscopy
**SURMOF** -	Surface-Mounted Metal-Organic Framework
**STED** -	Stimulated Emission Depletion Microscopy
**STM** -	Scanning Tunneling Microscopy
**STXM** -	Scanning Transmission X-ray Microscopy
**TCSPC** -	Time-Correlated Single Photon Counting
**TAM** -	Transient Absorption Microscopy
**tapa-ope** -	tris-(4-aminophenyl)amine and substituted oligo-(p- phenyleneethynylenes)
**TEM** -	Transmission Electron Microscopy
**TG-DSC** -	Thermogravimetric-Differential Scanning Calorimetry
**TPR** -	Temperature Programmed Reduction
**THz** -	Terahertz
**THz-TDS** -	Terahertz time-domain spectroscopy
**THz-STM –**	Terahertz STM
**TpmC***	1,1′,1′′-methanetriyltris(3,5-dimethyl-1*H*-pyrazole-4-carboxylic acid)).
**ttftc**	tetrathiafulvalene tetracarboxylate
**TXM** -	Transmission X-ray Microscopy
**UV-Vis** -	Ultraviolet-Visible Spectroscopy
**WAXS** -	Wide-Angle X-ray Scattering
**XAFS** -	X-ray Absorption Fine Structure Spectroscopy
**XAFS-CT** -	X-ray Absorption Fine Structure Computed Tomography
**XANES** -	X-ray Absorption Near Edge Structure
**XAS** -	X-ray Absorption Spectroscopy
**XES** -	X-ray Emission Spectroscopy
**XFEL** -	X-ray free-electron laser
**XPS** -	X-ray Photoelectron Spectroscopy
**XRD** -	X-Ray Diffraction
**ZIF** -	Zeolitic Imidazolate Framework

4 References

1. Enderlein, J. Advanced Fluorescence Microscopy. in *Comprehensive Biomedical Physics* (ed. Verveer, P. J.) **4**, 111–151 (Springer New York, 2014).
2. *Super-Resolution Microscopy*. (Wiley-VCH -).
3. *Fluorescent Microscopy*. *SpringerReference* **2440**, (Springer US, 2011).
4. *Light Sheet Fluorescence Microscopy*. (Wiley-VCH Verlag, 2024).
5. Dong, B., Mansour, N., Huang, T. X., Huang, W. & Fang, N. Single molecule fluorescence imaging of nanoconfinement in porous materials. *Chem Soc Rev* **50**, 6483–6506 (2021).
6. Shan, Y., Zhang, G., Shi, Y. & Pang, H. Synthesis and catalytic application of defective MOF materials. *Cell Rep Phys Sci* **4**, 101301 (2023).
7. Chen, L., Liu, D., Peng, J., Du, Q. & He, H. Ratiometric fluorescence sensing of metal-organic frameworks: Tactics and perspectives. *Coord Chem Rev* **404**, 213113 (2020).
8. Xu, X., Ma, M., Sun, T., Zhao, X. & Zhang, L. Luminescent Guests Encapsulated in Metal–Organic Frameworks for Portable Fluorescence Sensor and Visual Detection Applications: A Review. *Biosensors (Basel)* **13**, 435 (2023).
9. Wang, J. X., Yin, J., Shekhah, O., Bakr, O. M., Eddaoudi, M. & Mohammed, O. F. Energy Transfer in Metal-Organic Frameworks for Fluorescence Sensing. *ACS Appl Mater Interfaces* **14**, 9970–9986 (2022).
10. Salzer, Reiner. & Siesler, H. W. . *Infrared and Raman Spectroscopic Imaging: Second Edition*. *Infrared and Raman Spectroscopic Imaging: Second Edition* **9783527336**, (Wiley-VCH, 2014).
11. Cerasale, D. J., Ward, D. C. & Easun, T. L. MOFs in the time domain. *Nature Reviews Chemistry 2021 6:1* **6**, 9–30 (2021).
12. Menzies, A. C. The Raman effect. *Nature* **125**, 205–207 (1930).
13. *Infrared and Raman Characteristic Group Frequencies*.
14. Ma, L., Liu, M., Zhou, X., Li, C. & Wang, T. Metal-organic framework-based SERS sensing platforms for life and health detection. *Mater Chem Front* **7**, 4880–4899 (2023).
15. Zhang, Y., Xue, C., Xu, Y., Cui, S., Ganeev, A. A., Kistenev, Y. V., Gubal, A., Chuchina, V., Jin, H. & Cui, D. Metal-organic frameworks based surface-enhanced Raman spectroscopy technique for ultra-sensitive biomedical trace detection. *Nano Res* **16**, 2968–2979 (2023).
16. Chang, K., Zhao, Y., Wang, M., Xu, Z., Zhu, L., Xu, L. & Wang, Q. Advances in metal-organic framework-plasmonic metal composites based SERS platforms: Engineering strategies in chemical sensing, practical applications and future perspectives in food safety. *Chemical Engineering Journal* **459**, 141539 (2023).
17. Lai, H., Li, G., Xu, F. & Zhang, Z. Metal–organic frameworks: opportunities and challenges for surface-enhanced Raman scattering – a review. *J Mater Chem C Mater* **8**, 2952–2963 (2020).
18. Tittel, J., Knechtel, F. & Ploetz, E. Conquering Metal–Organic Frameworks by Raman Scattering Techniques. *Adv Funct Mater* 2307518 (2023). doi:10.1002/ADFM.202307518
19. Jeong, K., Arami-Niya, A., Yang, X., Xiao, G., Lipinski, G., Aman, Z. M., May, E. F., Richter, M. & Stanwix, P. L. Direct characterization of gas adsorption and phase transition of a metal organic framework using in-situ Raman spectroscopy. *Chemical Engineering Journal* **473**, 145240 (2023).
20. Taylor, R. W. & Sandoghdar, V. Interferometric Scattering (iSCAT) Microscopy and Related Techniques. in 25–65 (Springer, Cham, 2019). doi:10.1007/978-3-030-21722-8_2

21. *Label-Free Super-Resolution Microscopy*. (Springer International Publishing, 2019). doi:10.1007/978-3-030-21722-8
22. Gruszka, D. & Hundt, N. Label-free, mass-sensitive single-molecule imaging using interferometric scattering microscopy. *Essays Biochem* **65**, 81–91 (2021).
23. Hsieh, C. L. Label-free, ultrasensitive, ultrahigh-speed scattering-based interferometric imaging. *Opt Commun* **422**, 69–74 (2018).
24. Young, G. & Kukura, P. Interferometric Scattering Microscopy. *Annu Rev Phys Chem* **70**, 301–322 (2019).
25. Taylor, R. W. & Sandoghdar, V. Interferometric Scattering Microscopy: Seeing Single Nanoparticles and Molecules via Rayleigh Scattering. *Nano Lett* **19**, 4827–4835 (2019).
26. Murphy, D. B. & Davidson, M. W. *Fundamentals of Light Microscopy and Electronic Imaging: Second Edition*. *Fundamentals of Light Microscopy and Electronic Imaging: Second Edition* (John Wiley and Sons, 2012). doi:10.1002/9781118382905
27. Fakhrullin, R., Nigamatzyanova, L. & Fakhrullina, G. Dark-field/hyperspectral microscopy for detecting nanoscale particles in environmental nanotoxicology research. *Science of the Total Environment* **772**, 145478 (2021).
28. Gao, P. F., Lei, G. & Huang, C. Z. Dark-Field Microscopy: Recent Advances in Accurate Analysis and Emerging Applications. *Anal Chem* **93**, 4707–4726 (2021).
29. Gould, T. J., Schroeder, L. K., Pellett, P. A. & Bewersdorf, J. STED Microscopy. in *Fluorescence Microscopy: From Principles to Biological Applications: Second Edition* 321–338 (John Wiley & Sons, Ltd, 2017). doi:10.1002/9783527687732.ch10
30. Sahl, S. J. & Hell, S. W. High-Resolution 3D Light Microscopy with STED and RESOLFT. in *High Resolution Imaging in Microscopy and Ophthalmology* 3–32 (Springer, Cham, 2019). doi:10.1007/978-3-030-16638-0_1
31. Lanzano, L., Vicidomini, G., Scipioni, L., Castello, M. & Diaspro, A. STED microscopy: Exploring fluorescence lifetime gradients for super-resolution at reduced illumination intensities. in *Multiphoton Microscopy and Fluorescence Lifetime Imaging: Applications in Biology and Medicine* 85–102 (Walter de Gruyter GmbH, 2018). doi:10.1515/9783110429985-007
32. Egner, A., Geisler, C. & Siegmund, R. STED nanoscopy. in *Topics in Applied Physics* **134**, 3–34 (Springer, 2020).
33. Blom, H. & Widengren, J. Stimulated Emission Depletion Microscopy. *Chem Rev* **117**, 7377–7427 (2017).
34. Liu, Y., Peng, Z., Peng, X., Yan, W., Yang, Z. & Qu, J. Shedding New Lights Into STED Microscopy: Emerging Nanoprobes for Imaging. *Front Chem* **9**, 641330 (2021).
35. Wu, Z., Xu, X. & Xi, P. Stimulated emission depletion microscopy for biological imaging in four dimensions: A review. *Microsc Res Tech* **84**, 1947–1958 (2021).
36. Busko, D., Baluschev, S., Crespy, D., Turshatov, A. & Landfester, K. *New possibilities for materials science with STED microscopy*. *Micron* **43**, (Pergamon, 2012).
37. Ströhl, F. & Kaminski, C. F. *Frontiers in structured illumination microscopy*. *Optica* **3**, (Optica Publishing Group, 2016).
38. Ma, Y., Wen, K., Liu, M., Zheng, J., Chu, K., Smith, Z. J., Liu, L. & Gao, P. Recent advances in structured illumination microscopy. *JPhys Photonics* **3**, 024009 (2021).
39. Chen, X., Zhong, S., Hou, Y., Cao, R., Wang, W., Li, D., Dai, Q., Kim, D. & Xi, P. Superresolution structured illumination microscopy reconstruction algorithms: a review. *Light Sci Appl* **12**, 1–34 (2023).

40. *Metal-Organic Frameworks for Chemical Reactions*. *Metal-Organic Frameworks for Chemical Reactions* (Elsevier, 2021). doi:10.1016/c2019-0-04610-9
41. Navalón, S., Dhakshinamoorthy, A., Álvaro, M., Ferrer, B. & García, H. Metal-Organic Frameworks as Photocatalysts for Solar-Driven Overall Water Splitting. *Chem Rev* **123**, 445–490 (2023).
42. Ruckebusch, C., Sliwa, M., Pernot, P., de Juan, A. & Tauler, R. Comprehensive data analysis of femtosecond transient absorption spectra: A review. *Journal of Photochemistry and Photobiology C: Photochemistry Reviews* **13**, 1–27 (2012).
43. Li, N., Ma, Y. & Sun, W. Exploring the Dynamics of Charge Transfer in Photocatalysis: Applications of Femtosecond Transient Absorption Spectroscopy. *Molecules* **29**, 3995 (2024).
44. Qahtan, T. F., Owolabi, T. O., Olubi, O. E. & Hazam, A. Emerging tandem S-scheme heterojunction photocatalysts. *Coord Chem Rev* **514**, 215839 (2024).
45. Afrin, S., Yang, X., Morris, A. J. & Grumstrup, E. M. Rapid Exciton Transport and Structural Defects in Individual Porphyrinic Metal Organic Framework Microcrystals. *J Am Chem Soc* **146**, 4309–4313 (2024).
46. Demchenko, A. P. Frontiers for Future Research. Two-Photonic, Highly Excited and Single-Molecular Sensors. *Introduction to Fluorescence Sensing* 613–646 (2020). doi:10.1007/978-3-030-60155-3_16
47. Maris, J. J. E., Fu, D., Meirer, F. & Weckhuysen, B. M. Single-molecule observation of diffusion and catalysis in nanoporous solids. *Adsorption 2021 27:3* **27**, 423–452 (2021).
48. Messina, M. S. & Chang, C. J. Chemical Sensors and Imaging: Molecular, Materials, and Biological Platforms. *ACS Cent Sci* **9**, 1706–1711 (2023).
49. Ahn, Y., Park, M. & Seo, D. Observation of reactions in single molecules/nanoparticles using light microscopy. *Bull Korean Chem Soc* **44**, 35–44 (2023).
50. Liu, Y., Cui, S., Ma, W., Wu, Y., Xin, R., Bai, Y., Chen, Z., Xu, J. & Ge, J. Direct Imaging of Protein Clusters in Metal−Organic Frameworks. *J Am Chem Soc* **146**, 12565–12576 (2024).
51. Chen, Y., Li, Z., Huang, X., Lu, G. & Huang, W. Single-molecule mapping of catalytic reactions on heterostructures. *Nano Today* **34**, 100957 (2020).
52. Yan, R., Moon, S., Kenny, S. J. & Xu, K. Spectrally Resolved and Functional Super-resolution Microscopy via Ultrahigh-Throughput Single-Molecule Spectroscopy. *Acc Chem Res* **51**, 697–705 (2018).
53. Dong, B., Mansour, N., Huang, T. X., Huang, W. & Fang, N. Single molecule fluorescence imaging of nanoconfinement in porous materials. *Chem Soc Rev* **50**, 6483–6506 (2021).
54. Bian, K., Gerber, C., Heinrich, A. J., Müller, D. J., Scheuring, S. & Jiang, Y. Scanning probe microscopy. *Nature Reviews Methods Primers* **1**, (2021).
55. Haugstad, G. Atomic Force Microscopy: Understanding Basic Modes and Advanced Applications. *Atomic Force Microscopy: Understanding Basic Modes and Advanced Applications* (2012). doi:10.1002/9781118360668
56. Haugstad, G. Atomic Force Microscopy: Understanding Basic Modes and Advanced Applications. *Atomic Force Microscopy: Understanding Basic Modes and Advanced Applications* (2012). doi:10.1002/9781118360668
57. Nguyen-Tri, P., Ghassemi, P., Carriere, P., Nanda, S., Assadi, A. A. & Nguyen, D. D. Recent Applications of Advanced Atomic Force Microscopy in Polymer Science: A Review. *Polymers (Basel)* **12**, 1142 (2020).

58. Kosareva, E. K., Pivkina, A. N. & Muravyev, N. V. Atomic force microscopy in energetic materials research: A review. *Energetic Materials Frontiers* **3**, 290–302 (2022).
59. Pham, S. T., Tieu, A. K., Sun, C., Wan, S. & Collins, S. M. Direct Visualization of Chemical Transport in Solid-State Chemical Reactions by Time-of-Flight Secondary Ion Mass Spectrometry. *Nano Lett* **24**, 3702–3709 (2024).
60. Sabale, S., Barpaga, D., Yao, J., Kovarik, L., Zhu, Z., Chatterjee, S., McGrail, B. P., Motkuri, R. K. & Yu, X. Y. Understanding Time Dependence on Zinc Metal-Organic Framework Growth Using in Situ Liquid Secondary Ion Mass Spectrometry. *ACS Appl Mater Interfaces* **12**, 5090–5098 (2020).
61. Miller, M. K. & Forbes, R. G. Introduction to Atom-Probe Tomography. *Atom-Probe Tomography* 1–49 (2014). doi:10.1007/978-1-4899-7430-3_1
62. Gault, B., Moody, M. P., Cairney, J. M. & Ringer, S. P. Atom Probe Microscopy. **160**, (2012).
63. Gault, B., Chiaramonti, A., Cojocaru-Mirédin, O., Stender, P., Dubosq, R., Freysoldt, C., Makineni, S. K., Li, T., Moody, M. & Cairney, J. M. Atom probe tomography. *Nature Reviews Methods Primers 2021 1:1* **1**, 1–30 (2021).
64. Li, T., Devaraj, A. & Kruse, N. Atomic-scale characterization of (electro-)catalysts and battery materials by atom probe tomography. *Cell Rep Phys Sci* **3**, 101188 (2022).
65. Liang, K., Bi, L., Zhu, Q., Zhou, H. & Li, S. Ultrafast Dynamics Revealed with Time-Resolved Scanning Tunneling Microscopy: A Review. *ACS Applied Optical Materials* **1**, 924–938 (2023).
66. Binning, G., Rohrer, H., Gerber, C. & Weibel, E. Surface studies by scanning tunneling microscopy. *Phys Rev Lett* **49**, 57–61 (1982).
67. Binnig, G. & Rohrer, H. Scanning tunneling microscopy from birth to adolescence. *Rev Mod Phys* **59**, 615–625 (1987).
68. Bazylewski, P., Ezugwu, S. & Fanchini, G. A Review of Three-Dimensional Scanning Near-Field Optical Microscopy (3D-SNOM) and Its Applications in Nanoscale Light Management. *Applied Sciences* **7**, 973 (2017).
69. Raschke, M. B., Schubert, M., Narang, P. & Paarman, A. Optical nanoprobe imaging and spectroscopy. *Appl Phys Lett* **123**, (2023).
70. Dazzi, A. & Prater, C. B. AFM-IR: Technology and applications in nanoscale infrared spectroscopy and chemical imaging. *Chem Rev* **117**, 5146–5173 (2017).
71. Schwartz, J. J., Jakob, D. S. & Centrone, A. A guide to nanoscale IR spectroscopy: resonance enhanced transduction in contact and tapping mode AFM-IR. *Chem Soc Rev* **51**, 5248–5267 (2022).
72. Mathurin, J., Deniset-Besseau, A., Bazin, D., Dartois, E., Wagner, M. & Dazzi, A. Photothermal AFM-IR spectroscopy and imaging: Status, challenges, and trends. *J Appl Phys* **131**, 10901 (2022).
73. Spence, J. C. H. *High-Resolution Electron Microscopy*. (Oxford University Press).
74. Erdman, N., Bell, D. C. & Reichelt, R. *Scanning Electron Microscopy*. *Springer Handbooks* (Springer, Cham, 2019). doi:10.1007/978-3-030-00069-1_5
75. Zhou, Y., Dong, Z., Terasaki, O. & Ma, Y. Electron Microscopy of Nanoporous Crystals. *Acc Mater Res* **3**, 110–121 (2022).
76. Zhan, Z., Liu, Y., Wang, W., Du, G., Cai, S. & Wang, P. Atomic-level imaging of beam-sensitive COFs and MOFs by low-dose electron microscopy. *Nanoscale Horiz* **9**, 900–933 (2024).
77. Liu, L., Zhang, D., Zhu, Y. & Han, Y. Bulk and local structures of metal–organic frameworks unravelled by high-resolution electron microscopy. *Commun Chem* **3**, 1–14 (2020).

78. Williams, D. B. & Carter, C. B. *Transmission electron microscopy: A textbook for materials science*. *Transmission Electron Microscopy: A Textbook for Materials Science* (Springer US, 2009). doi:10.1007/978-0-387-76501-3
79. Nellist, P. D. *Scanning transmission electron microscopy*. *Springer Handbooks* (Springer, 2019). doi:10.1007/978-3-030-00069-1_2
80. Zhang, J., Cheng, N. & Ge, B. Characterization of metal-organic frameworks by transmission electron microscopy. *Adv Phys X* **7**, 2046157 (2022).
81. *Principles of Analytical Electron Microscopy*. *Principles of Analytical Electron Microscopy* (Springer US, 1986). doi:10.1007/978-1-4899-2037-9
82. Ahn, C. C. . *Transmission Electron Energy Loss Spectrometry in Materials Science and The EELS Atlas*. (Wiley, 2004). doi:10.1002/3527605495
83. Chao, H. Y., Venkatraman, K., Moniri, S., Jiang, Y., Tang, X., Dai, S., Gao, W., Miao, J. & Chi, M. In Situ and Emerging Transmission Electron Microscopy for Catalysis Research. *Chem Rev* **123**, 8347–8394 (2023).
84. Jacobsen, C. *X-ray microscopy*. *X-ray Microscopy* (Cambridge University Press, 2019). doi:10.1017/9781139924542
85. Petersen, H. & Weidenthaler, C. A review of recent developments for the in situ/operando characterization of nanoporous materials. *Inorg Chem Front* **9**, 4244–4271 (2022).
86. Zhao, X. & Miao, X. Surface-supported metal–organic frameworks with geometric topological diversity via scanning tunneling microscopy. *iScience* **27**, (2024).
87. Wang, M., Dong, R. & Feng, X. Two-dimensional conjugated metal-organic frameworks (2Dc-MOFs): chemistry and function for MOFtronics. *Chem Soc Rev* **50**, 2764–2793 (2021).
